# Flavin-Containing Monooxygenases Are Conserved Regulators of Stress Resistance and Metabolism

**DOI:** 10.3389/fcell.2021.630188

**Published:** 2021-02-12

**Authors:** Shijiao Huang, Marshall B. Howington, Craig J. Dobry, Charles R. Evans, Scott F. Leiser

**Affiliations:** ^1^Department of Molecular and Integrative Physiology, University of Michigan, Ann Arbor, MI, United States; ^2^Cellular and Molecular Biology Program, University of Michigan, Ann Arbor, MI, United States; ^3^Department of Internal Medicine, University of Michigan, Ann Arbor, MI, United States

**Keywords:** mammalian cells, stress, metabolism, FMO, *C. elegans*, longevity

## Abstract

Flavin-Containing Monooxygenases are conserved xenobiotic-detoxifying enzymes. Recent studies have revealed endogenous functions of FMOs in regulating longevity in *Caenorhabditis elegans* and in regulating aspects of metabolism in mice. To explore the cellular mechanisms of FMO’s endogenous function, here we demonstrate that all five functional mammalian FMOs may play similar endogenous roles to improve resistance to a wide range of toxic stresses in both kidney and liver cells. We further find that stress-activated c-Jun N-terminal kinase activity is enhanced in FMO-overexpressing cells, which may lead to increased survival under stress. Furthermore, FMO expression modulates cellular metabolic activity as measured by mitochondrial respiration, glycolysis, and metabolomics analyses. FMO expression augments mitochondrial respiration and significantly changes central carbon metabolism, including amino acid and energy metabolism pathways. Together, our findings demonstrate an important endogenous role for the FMO family in regulation of cellular stress resistance and major cellular metabolic activities including central carbon metabolism.

## Introduction

Flavin-containing monooxygenases (FMOs) are primarily studied as xenobiotic-metabolizing enzymes that oxygenate substrates by adding a molecule of oxygen to nitrogen, sulfur or other soft-nucleophilic atoms to increase the solubility and excretion of xenobiotics (Ziegler et al., 1971; Poulsen et al., 1976; Poulsen and Ziegler, 1979). The oxygenation activity of FMOs is highly efficient, since it does not require the presence of a substrate to start the catalytic cycle. Utilizing flavin adenine dinucleotide (FAD) as a prosthetic group, NADPH as a hydride donor, and oxygen as a co-substrate, a stable C4a-hydroperoxyflavin [(FADH-OOH) (NADP+)] is formed and is primed to oxygenate any soft nucleophile-containing substrate that can access the active site of FMO (Beaty and Ballou, 1981; Jones and Ballou, 1986; Poulsen and Ziegler, 1995). This uniquely efficient catalytic mechanism is in contrast to cytochrome P450 (CYP), the major xenobiotic-metabolizing monooxygenase, which requires the binding of substrate to activate oxygen for oxygenation (Capdevila et al., 1984; Lu et al., 1984).

Flavin-containing monooxygenases are ancient enzymes present in all phyla so far examined, and they are widely conserved from bacteria to vertebrates. There is a single ancestral FMO in yeast (*Saccharomyces cerevisiae*), while there are multiple FMOs in nematodes (*Caenorhabditis elegans*), fruit flies (*Drosophila melanogaster*), mice (*Mus musculus*), and humans (*Homo sapiens*). The additional *FMO* genes may exist due to gene duplication events in evolutionary response to new xenobiotics in the environment (Hao da et al., 2009). Human *FMO1*, *FMO2*, *FMO3*, *FMO4*, and *FMO6* are clustered on the region q24.3 on Chromosome 1 (Hernandez et al., 2004). Human *FMO6* does not encode a functional protein thus was identified as a pseudo gene (Hines et al., 2002; Hernandez et al., 2004). Human *FMO5* is about 26 Mb away and is located in the region 1q21.1 on Chromosome 1 (Hernandez et al., 2004). Similarly, mouse *Fmo1*, *Fmo2*, *Fmo3*, *Fmo4*, and *Fmo6* are clustered on Chromosome 1, and mouse *Fmo5* is located outside of the *Fmo* cluster on Chromosome 3 (Hernandez et al., 2004). Mouse *Fmo6* is homologous in sequence to human *FMO6* but needs further investigation to classify whether it is a pseudo gene (Hernandez et al., 2004). The *C. elegans* genome contains five *FMOs*, arbitrarily numbered as *fmo-1* to *fmo-5*, but they are not counterparts to individual *FMOs* with the same number in human and mouse (Petalcorin et al., 2005). Nematode *fmo-1 – fmo-5* are paralogous to each other, and homologous to all mouse and human FMOs, with mammalian *FMO5* containing the highest sequence identity (Petalcorin et al., 2005; Nicoll et al., 2019).

Flavin-containing monooxygenases show developmental and tissue-specific expression in different organisms. Human *FMO1* expression is silenced in the adult liver but present in the kidney and small intestine (Yeung et al., 2000). In contrast, mouse *Fmo1* is highly expressed in the adult liver with expression also detected in kidney, lung, adipose tissue, and brain (Janmohamed et al., 2004; Veeravalli et al., 2014). The majority of humans do not express functional *FMO2* because of a mutation that has introduced a premature stop codon (Dolphin et al., 1998; Veeramah et al., 2008). *Fmo2* is most expressed in the lungs of mice (Siddens et al., 2008). FMO3 and FMO5 are the major forms of FMOs in the liver of humans (Dolphin et al., 1996; Overby et al., 1997) and mice (Cherrington et al., 1998). FMO4 expression is very low in multiple tissues of both humans and mice, with relatively higher expression in the adult liver and kidney (Janmohamed et al., 2004; Zhang and Cashman, 2006). FMO5 is most highly expressed in the liver of humans and mice and also shows expression in the gastrointestinal tract in both organisms (Scott et al., 2017; Zhang et al., 2018). In *C. elegans*, *fmo-1*, *fmo-2*, and *fmo-5* are expressed in the intestine, which is thought to be equivalent to human kidney and small and large intestines; *fmo-3* and *fmo-4* are expressed in *C. elegans* hypodermis, which is equivalent to human liver and adipose tissue (Petalcorin et al., 2005; Kaletsky et al., 2018).

How FMOs act within physiological processes is largely unknown except for the role of FMO3 in the conversion of odorous trimethylamine to non-odorous trimethylamine N-oxide (Dolphin et al., 1997; Lang et al., 1998). Failure to oxygenate the volatile substrate trimethylamine (TMA) to the soluble product trimethylamine–N-oxide (TMAO) is caused by human FMO3 mutations and leads to trimethylaminuria, previously known as “Fish odor syndrome” (Dolphin et al., 1997). FMO1, 2, 4 knockouts also show that in male mice, where FMO3 expression in the liver is normally not detected, FMO1 also plays a key role in metabolizing TMA to TMAO (Veeravalli et al., 2018). However, the percentage of excreted TMAO was far less in male than in female mice regardless of *Fmo* genotype, supporting FMO3 as the major TMA oxygenating enzyme (Veeravalli et al., 2018). FMO3 liver-specific knockdown and transgenic overexpression also show that FMO3 is involved in cholesterol balance and glucose and lipid metabolism (Shih et al., 2015; Warrier et al., 2015). Studies from the Shephard group utilizing FMO knockout mice also reveal that two knockout mouse models (FMO1, 2, and 4 knockout and FMO5 knockout) are each leaner with higher whole-body energy expenditure, indicating that FMO1 and FMO5 regulate energy balance and promote metabolic efficiency (Veeravalli et al., 2014; Gonzalez Malagon et al., 2015; Scott et al., 2017). Additionally, in FMO5 knockout mice, key enzymes for carbohydrate, fatty acid metabolism, and glycolysis are down-regulated, leading the authors to report FMO5 as a regulator of metabolic aging (Gonzalez Malagon et al., 2015; Scott et al., 2017). These reports suggest that FMOs are key regulators of metabolism and physiology in mice.

Recent reports reveal that FMOs also play important roles in aging in *C. elegans* and possibly in mice. *C. elegans fmo-2* is a longevity gene that is induced and required in the worm intestine by at least two longevity signaling pathways, hypoxia and dietary restriction, for lifespan extension. Furthermore, overexpression of *fmo-2* is sufficient to extend lifespan, improve healthspan, and increase stress resistance in *C. elegans* (Leiser et al., 2015). All five mouse FMOs are reported to be upregulated in long-lived mouse models (Swindell, 2009; Steinbaugh et al., 2012). This evidence supports a role for FMOs as pro-longevity and beneficial for health in both lower organisms and vertebrates, potentially through a conserved endogenous function. In contrast, FMO5 knockout mice have shown improved glucose homeostasis, generally a positive long-term health measure (Scott et al., 2017).

Earlier studies have shown that long-lived *C. elegans* and *Drosophila* mutant strains are more resistant to multiple stresses, including oxidative stress, heat, and UV light (Larsen, 1993; Lithgow et al., 1995; Murakami and Johnson, 1996; Lin et al., 1998). More recently, the Miller group showed that skin-derived fibroblasts from long-lived mice are resistant to lethal effects of multiple stressors, including cadmium, hydrogen peroxide (H_2_O_2_), and UV-radiation (Murakami et al., 2003; Salmon et al., 2005; Harper et al., 2006). They went on to show that cells from a variety of rodents and birds possess a strong correlation between the maximum lifespan of the organism and the stress resistance of their cells (Harper et al., 2011). These reports support the hypothesis that increased stress resistance to various environmental insults contributes to longer lifespan in different species.

We hypothesized that, like in *C. elegans*, one or more mammalian FMOs would improve resistance to stress and would likely do so by modifying endogenous metabolism. To test this, we overexpressed mouse FMO1, FMO2, FMO3, FMO4, and FMO5 in two cell types in which FMOs are most highly expressed. We examine cellular stress resistance and metabolic changes, with the aim to demonstrate the endogenous metabolic functions of FMOs and to determine whether there are differential effects when each FMO is expressed individually in the same cell line.

We demonstrate that increased FMO expression renders cells resistant to multiple stressors, including the heavy metals cadmium and arsenite, the free radical generator paraquat, UV-radiation, and the mitochondrial toxin rotenone. These results indicate that vertebrates with higher cellular levels of FMOs may be protected from damage. Through cellular metabolic activities and metabolomics analyses, we also show that FMOs significantly change cellular metabolism. It is imperative to test in the future whether regulation of metabolism by FMOs contribute to the stress resistance and potential benefits of FMOs in human health.

## Results

### FMOs Improve Stress Resistance to Oxidative Stress in *C. elegans* and in Mammalian Cells

**FIGURE 1:**
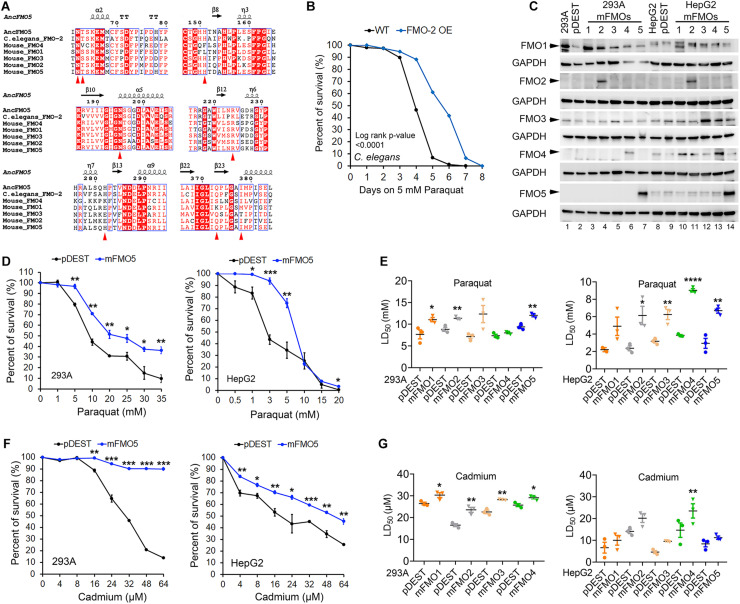
*C. elegans* FMO-2 and mammalian FMOs improve stress resistance to oxidative stress. **(A)** Amino acid sequence alignment of FMOs across species with the regions containing the eight essential residues denoted as red arrowheads in the catalytic active site among reconstructed ancestral mammalian FMO5, *C. elegans* FMO-2, and mouse FMO1–5. **(B)** Wild-Type and FMO-2 overexpressing (FMO-2 OE) worm survival curves on paraquat stress. Each strain was placed on NGM (Nematode Growth Medium) containing 5 mM paraquat from the fourth larvae stage (L4) at Day 0. Survival was quantified every day until all worms were dead. The difference between the survival curves is denoted with the log-rank test *p*-value. **(C)** FMO1–5 protein levels in FMO1–5 OE and control cells of both HEK293A and HepG2. **(D)** FMO5 OE and control cell survival curves on paraquat stress in HEK293A and HepG2. HEK293A cells or HepG2 cells stably expressing FMO5 or empty vector pDEST were subjected to indicated increasing doses of paraquat. **(E)** LD_50_ values of FMO1–5 OE cells compared to the control cells on paraquat stress in HEK293A and HepG2. HEK293A cells or HepG2 cells stably expressing FMO1–5 or empty vector pDEST were subjected to indicated increasing doses of paraquat. **(F)** FMO5 OE and control cell survival curves on cadmium stress in HEK 293A and HepG2. HEK293A cells or HepG2 cells stably expressing FMO5 or empty vector pDEST were subjected to indicated increasing doses of cadmium. **(G)** LD_50_ values of FMO-OE cells compared to the control cells on cadmium stress in HEK293A and HepG2. HEK293A cells or HepG2 cells stably expressing FMO or empty vector pDEST were subjected to indicated increasing doses of paraquat. Data represent mean ± SEM. *p < 0.05, **p < 0.01, ***p < 0.001, and ****p < 0.0001.

Recently, the crystal structure of three reconstructed, predicted ancestral FMO sequences were resolved, revealing that eight amino acids in the catalytic site, NADPH binding region, FAD binding region and the stabilization of the C4a-hydroperoxyflavin [(FADH-OOH) (NADP+)] intermediate are highly conserved among different FMOs (Nicoll et al., 2019). To explore the conservation of the catalytic active residues between *C. elegans* and mammalian FMOs, we aligned the regions containing these eight active residues (Asn62, Thr63, His151, Asn195, Arg224, His282, Gln373, and Ile378) among reconstructed ancestral mammalian FMO5, *C. elegans* FMO-2, and mouse FMO1–5 (Figure 1A). Full length amino acid alignment and sequence identities among *C. elegans* FMO-2 and mouse FMO1–5 are shown in Supplementary Figures 1A,B. Although the percent sequence identity is lower between *C. elegans* FMO-2 and mouse FMOs than within mouse FMOs, the catalytic residues of these proteins are much more highly conserved with only one catalytic residue difference (Supplementary Figures 1A,B and Figure 1A). This indicates that the enzymatic activity and function of *C. elegans* and mouse FMO could be conserved.

To test this possibility, we measured stress resistance in *C. elegans* and mammalian cell lines with FMO overexpression (Figures 1B,D–G). We previously reported that *C. elegans fmo-2* overexpression is sufficient to extend lifespan and improve resistance to proteotoxic stress induced by tunicamycin, heat, or dithiothreitol (DTT) (Leiser et al., 2015). To test whether this stress resistance is limited to just proteotoxic stress, we asked whether *fmo-2* expression also confers resistance to mitochondrial oxidative stress through the reactive oxygen species (ROS) inducer paraquat. The resulting data (Figure 1B) show that worms overexpressing *fmo-2* are more resistant to paraquat stress, consistent with the stress resistance phenotype being broad, and not specific to proteotoxic stress. This result, together with our previous findings, led to our interest in two primary questions: (1) whether mammalian FMOs confer similar benefits to mammals as nematode FMOs do to worms, and (2) which (if any) mammalian FMO is most likely functionally equivalent to nematode FMO-2?

To answer these questions, we utilized tissue culture to overexpress each of the five functional mouse FMOs individually in HEK293A kidney and HepG2 liver cells. The overexpression of different FMOs was confirmed by western blotting (Figure 1C) and quantitative PCR (Supplementary Figures 2A,B). Each FMO is overexpressed in its overexpressing cell lines (hereafter referred to as FMO-OE cells) in both HEK293A and HepG2. The expression pattern of endogenous FMO is consistent with the literature in that FMO1 is not expressed in adult liver cells (HepG2) and can be detected in kidney cells (HEK293A); FMO2 is not expressed both in liver and kidney cells; FMO3 is present in both kidney cells (HEK293A) and liver cells (HepG2); FMO4 is very low in kidney cells (HEK293A) but can be detected in liver cells (HepG2); FMO5 is highly expressed in liver cells (HepG2) but not detectable in kidney cells (HEK293A) (Figure 1C). With increasing doses of paraquat applied to FMO-OE cells or empty vector control cells, both HEK293A cells and HepG2 cells show the expected decreased survival rates (Figure 1D and Supplementary Figures 3A,B). Surprisingly, our results show that overexpression of FMO1, FMO2, FMO3, FMO4, and FMO5 each significantly and consistently increases cell survival in the tested dose responses to paraquat in both cell types (Figure 1D and Supplementary Figures 3A,B). We also compared the LD_50_ (Lethal Dose 50%) values, the dose of stress agent that lead to survival of 50% of the cells, for the FMO-OE and control cells (Figure 1E). FMO-OE cells were significantly more resistant to paraquat compared with control cells in both HEK293A and HepG2 (Figure 1E). The LD_50_, *t*-test *P* values, and percentage of LD_50_ increases of each FMO-OE cell line compared to the control cell line are summarized in Table 1A for HEK293A and Table 1B for HepG2.

**TABLE 1:** FMOs improve stress resistance to oxidative stress in HEK 293A and HepG2 cells.

A				

Stress	Cell lines 293A	LD_50_ (mM) Mean ± SED	*t*-Test *P* Value	Increase, %
Paraquat	pDEST	7.66 ± 1.03	–	–
	mFMO1	11.1 ± 0.48	0.040	44.3%
Paraquat	pDEST	8.83 ± 0.46	–	–
	mFMO2	11.4 ± 0.22	0.008	28.6%
Paraquat	pDEST	7.15 ± 0.47	–	–
	mFMO3	12.3 ± 1.97	0.063	n.s.
Paraquat	pDEST	7.35 ± 0.34	–	–
	mFMO4	8.00 ± 0.18	0.165	n.s.
Paraquat	pDEST	9.38 ± 0.38	–	–
	mFMO5	12.0 ± 0.32	0.006	28.1%

**B**				

**Stress**	**Cell lines HepG2**	**LD_50_ (mM) Mean ± SED**	***t*-Test *P* Value**	**Increase, %**

Paraquat	pDEST	2.20 ± 0.24	–	–
	mFMO1	4.89 ± 1.05	0.145	n.s.
Paraquat	pDEST	2.36 ± 0.29	–	–
	mFMO2	6.13 ± 1.06	0.026	159.8%
Paraquat	pDEST	3.17 ± 0.20	–	–
	mFMO3	6.24 ± 0.59	0.008	97.2%
Paraquat	pDEST	3.85 ± 0.08	–	–
	mFMO4	9.03 ± 0.19	<0.0001	134.2%
Paraquat	pDEST	2.93 ± 0.57	–	–
	mFMO5	6.69 ± 0.29	0.004	128.6%

**C**				

**Stress**	**Cell lines 293A**	**LD_50_ (μM) Mean ± SED**	***t*-Test *P* Value**	**Increase, %**

Cadmium	pDEST	26.5 ± 0.61	–	–
	mFMO1	30.3 ± 1.11	0.038	14.6%
Cadmium	pDEST	16.5 ± 0.67	–	–
	mFMO2	23.6 ± 1.15	0.006	42.5%
Cadmium	pDEST	22.6 ± 0.82	–	–
	mFMO3	28.2 ± 0.12	0.002	24.9%
Cadmium	pDEST	25.7 ± 0.82	–	–
	mFMO4	29.1 ± 0.59	0.019	13.2%
Cadmium	pDEST	26.6 ± 0.60	–	–
	mFMO5	>64.0	–	>140.6%

**D**				

**Stress**	**Cell lines HepG2**	**LD_50_ (μM) Mean ± SED**	***t*-Test *P* Value**	**Increase, %**

Cadmium	pDEST	6.59 ± 2.49	–	–
	mFMO1	9.99 ± 2.24	0.367	n.s.
Cadmium	pDEST	14.1 ± 1.01	–	–
	mFMO2	20.1 ± 1.98	0.052	n.s.
Cadmium	pDEST	4.58 ± 0.67	–	–
	mFMO3	9.50 ± 0.15	0.002	107.4%
Cadmium	pDEST	14.7 ± 3.33	–	–
	mFMO4	23.5 ± 3.28	0.132	n.s.
Cadmium	pDEST	8.35 ± 1.37	–	–
	mFMO5	11.2 ± 0.75	0.142	n.s.

***(A,B)** LD_50_, t-test P values, and percentage of LD50 increase of FMO1–5 OE cell line compared to the control cell line on paraquat stress in HEK293A and HepG2. HEK293A cells **(A)** or HepG2 cells **(B)** stably expressing FMO1–5 or empty vector pDEST were subjected to indicated increasing doses of paraquat. **(C,D)** LD_50_, t-test P values, and percentage of LD_50_ increases of FMO1–5 OE cell line compared to the control cell line on cadmium stress in HEK293A and HepG2. HEK293A cells **(C)** or HepG2 cells **(D)** stably expressing FMO1–5 or empty vector pDEST were subjected to indicated increasing doses of cadmium.*

Next, we tested cell survival under cadmium, which indirectly produces ROS and induces oxidative stress. Cadmium is a heavy metal with extensive use in industry and has become a ubiquitous environmental toxicant. Cadmium accumulates mainly in the liver and kidney and causes lasting damage, in part due to its long half-life (Satarug et al., 2010). Similar to paraquat, we find that all five FMO-OE cell lines show improved stress resistance to cadmium in both HEK293A kidney cells and HepG2 liver cells (Figure 1F and Supplementary Figures 3C,D). LD_50_ values of FMO1–4 OE in HEK293A cells are significantly higher than the control cells (Figure 1G). FMO5-OE increases resistance so much that the survival rate of FMO5-OE cells is greater than 50% even at the highest dose of 64 μM, meaning LD_50_ cannot be accurately calculated (Figure 1F). In HepG2 cells, FMO-OE cells are significantly more resistant than the control cells in response to cadmium at individual doses (Figure 1F and Supplementary Figure 3D). The cadmium LD_50_ of FMO1, 2, 3, and 5 OE cells is increased compared to the control cells but did not reach statistical significance, while FMO4-OE is significant (Figure 1G). The values of LD_50_, significance, and percentage of LD_50_ increases for FMO-OE and control cells in response to cadmium are listed in Tables 1C,D. We note that while each FMO-OE cell line exhibits improved resistance to cadmium and paraquat, we observed variability between the lines (e.g., FMO5 improved the cadmium stress resistance the most compared to other FMOs in HEK293A cells by more than 140.6% as shown in Tables 1C,D). We next applied a mitochondrial respiration chain complex I inhibitor, rotenone, and found that FMO-OE cells also exhibited increased survival in both cell types (Supplementary Figures 5A,B), albeit to a lesser extent. These results demonstrate that the role of FMOs in increasing resistance to oxidative stress is likely conserved from *C. elegans* to mammals. These data also show that, contrary to our initial hypothesis, all five mouse FMO-OE cell lines are similar to *C. elegans fmo-2* overexpressing worms in resisting stresses, and any mouse FMO could be functionally equivalent to *C. elegans fmo-2* in stress resistance.

### FMOs Confer Resistance to Broad Stressors Including Arsenite and UV-Radiation

**FIGURE 2:**
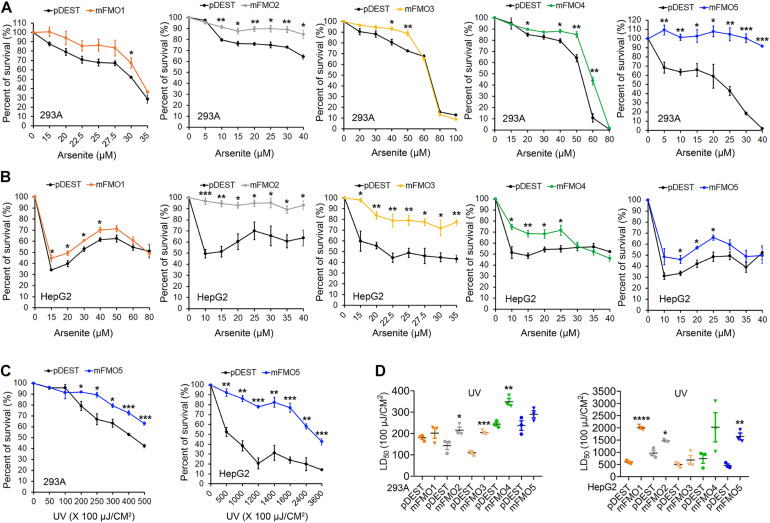
FMOs improve stress resistance to broader stressors in mammalian cells. **(A,B)** FMO1–5 OE and control cell survival curves on arsenite stress in HEK293A and HepG2. HEK293A cells **(A)** or HepG2 cells **(B)** stably expressing FMO1–5 or empty vector pDEST were subjected to indicated increasing doses of arsenite. **(C)** FMO5 OE and control cell survival curves on UV-radiation in HEK 293A and HepG2. HEK293A cells or HepG2 cells stably expressing FMO5 or empty vector pDEST were subjected to indicated increasing energies of UV-radiation. **(D)** LD_50_ values of FMO1–5 OE cells compared to the control cells on UV-radiation in HEK293A and HepG2. HEK293A cells or HepG2 cells stably expressing FMO1–5 or empty vector pDEST were subjected to indicated increasing energies of UV-radiation. Data represent mean ± SEM. *p < 0.05, **p < 0.01, ***p < 0.001, and ****p < 0.0001.

Fibroblasts isolated from long-lived organisms are resistant to multiple stressors compared to their shorter-lived evolutionary closely related species (Murakami et al., 2003; Salmon et al., 2005; Harper et al., 2006; Wang and Miller, 2012; Ozkurede and Miller, 2019). This broad stress resistance is a frequent marker of longevity, while resistance to specific stressors often marks more narrow evolutionary adaptations. Since FMOs are known to detoxify xenobiotics, we posited that while paraquat has not been ruled out as an FMO substrate, FMOs are unlikely to detoxify heavy metals, such as cadmium and arsenite, and could not reasonably block UV-radiation. Thus, to further test the hypothesis that FMOs have a conserved and broad role in stress resistance, we asked whether FMO-OE cells demonstrate resistance to the broad stressors arsenite and UV-radiation. Arsenite causes a variety of stress responses, including oxidative stress, heat-stress response, and cytoplasmic stress granule formation (Bernstam and Nriagu, 2000; Chen and Liu, 2017). Our data show that increased expression of each FMO significantly improves cell survival rates for at least one dose of arsenite in both HEK293A (Figure 2A and Supplementary Figure 4A) and HepG2 cells (Figure 2B), with FMO5 resisting arsenite most in HEK293A cells by more than 130.1% (Table 2A). We next tested cell survival under UV-radiation, which causes both protein and DNA damage through cross-linking. Consistently, each of the FMO-OE cell lines shows increased resistance to UV light compared to the control in both cell types (Supplementary Figures 4B,C and Figures 2C,D), with FMO5 expression again increasing resistance most, this time in HepG2 cells by 261% (Tables 2B,C). Together, our results indicate that exogenously expressing FMOs in kidney and liver cells render their stress resistance signatures similar to fibroblasts from long-lived organisms.

**TABLE 2:** FMOs improve stress resistance to broader stressors in mammalian cells.

A				

Stress	Cell lines 293A	LD_50_ (μM) Mean ± SED	*t*-Test *P* Value	Increase, %
Arsenite	pDEST	25.2 ± 0.13	–	–
	mFMO1	28.3 ± 0.98	0.033	12.6%
Arsenite	pDEST	>40	–	–
	mFMO2	>40	–	–
Arsenite	pDEST	58.5 ± 1.02	–	–
	mFMO3	62.4 ± 0.53	0.026	6.80%
Arsenite	pDEST	49.7 ± 1.65	–	–
	mFMO4	58.2 ± 0.70	0.009	17.1%
Arsenite	pDEST	17.4 ± 3.72	–	–
	mFMO5	>40	–	>130.1%

**B**				

**Stress**	**Cell lines 293A**	**LD_50_ (100 μJ/CM^2^) Mean ± SED**	***t*-Test *P* Value**	**Increase, %**

UV	pDEST	180.0 ± 7.87	–	–
	mFMO1	201.9 ± 23.8	0.431	n.s.
UV	pDEST	143.1 ± 18.1	–	–
	mFMO2	215.4 ± 15.2	0.038	50.5%
UV	pDEST	109.6 ± 6.54	–	–
	mFMO3	204.1 ± 6.37	0.0005	86.2%
UV	pDEST	244.0 ± 8.81	–	–
	mFMO4	348.7 ± 15.4	0.004	42.9%
UV	pDEST	237.2 ± 22.4	–	–
	mFMO5	289.8 ± 16.9	0.134	n.s.

**C**				

**Stress**	**Cell lines HepG2**	**LD_50_ (100 μJ/CM^2^) Mean ± SED**	***t*-Test *P* Value**	**Increase, %**

UV	pDEST	602.7 ± 37.6	–	–
	mFMO1	2006 ± 52.9	<0.0001	233%
UV	pDEST	966.6 ± 111	–	–
	mFMO2	1466 ± 28.5	0.012	51.7%
UV	pDEST	498.3 ± 55.4	–	–
	mFMO3	681.4 ± 188	0.403	n.s.
UV	pDEST	740.5 ± 193	–	–
	mFMO4	2027 ± 599	0.110	n.s.
UV	pDEST	456.9 ± 59.8	–	–
	mFMO5	1650 ± 140	0.001	261%

***(A)** LD_50_, t-test P values, and percentage of LD_50_ increases of FMO1–5 OE cell line compared to the control cell line on arsenite stress in HEK293A. HEK293A cells stably expressing FMO1–5 or empty vector pDEST were subjected to indicated increasing doses of arsenite. **(B,C)** LD_50_, t-test P values, and percentage of LD_50_ increases of FMO1–5 OE cell line compared to the control cell line on UV-radiation in HEK293A and HepG2. HEK293A cells **(B)** or HepG2 cells **(C)** stably expressing FMO1–5 or empty vector pDEST were subjected to indicated increasing energies of UV-radiation.*

### JNK Kinase Activity Is Increased in FMO-Overexpressing Cells Under Cadmium-Induced Oxidative Stress

We were next interested in the mechanism that FMOs act through to resist stress. A subfamily of mitogen-activated protein kinases (MAPKs) activated specifically by stress are the stress-activated protein kinases (SAPKs). c-Jun N-terminal kinases (JNKs), the enzymes that phosphorylate the transcriptional factor AP-1(c-Jun), and p38 kinases are the two best characterized SAPKs. These enzymes are responsive to multiple stressors including inflammatory cytokines, UV-radiation, and oxidative stress. The extracellular signal-regulated kinase (ERK), another member of MAPK, also responds to stress but is more commonly linked to growth factor stimuli and cell proliferation regulation (Cicenas et al., 2017; Corre et al., 2017). JNK, p38, and ERK are reportedly activated by cadmium (Chuang et al., 2000; Escobar Mdel et al., 2009; Zhao et al., 2015; Tsai et al., 2016). Interestingly, JNK and p38 are also reportedly activated by paraquat, arsenite, UV, and rotenone (Derijard et al., 1994; Cavigelli et al., 1996; Newhouse et al., 2004; Peng et al., 2004; Klintworth et al., 2007) each of which FMO-OE cell lines resist as shown in Figures 1, 2 and Supplementary Figures 3–5.

**FIGURE 3:**
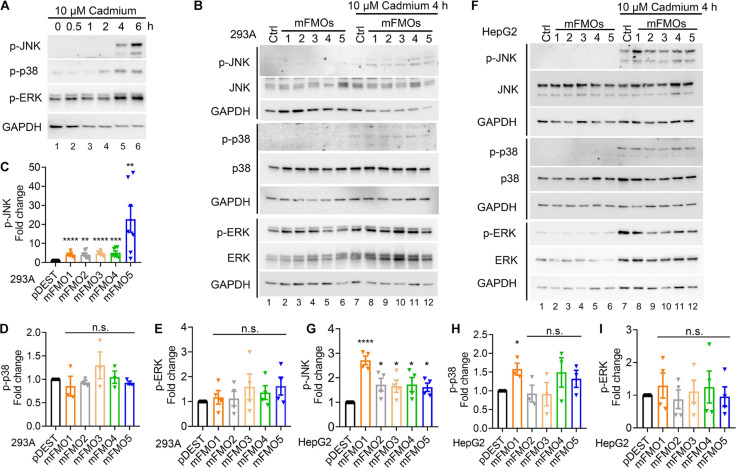
JNK kinase activity is increased in FMO-overexpressing cells under cadmium-induced oxidative stress. **(A)** SAPK levels and phosphorylation including JNK, p38, and ERK after 10 μM cadmium treatment of indicated time. HEK293A cells were treated with cadmium, and the SAPK activities were measured with antibodies against phosphorylated SAPKs. **(B)** SAPKs levels and phosphorylation including JNK, p38, and ERK in FMO1–5 OE HEK293A cells and empty vector control cells after 10 μM Cadmium treatment for 4 h. Quantitation of the phosphorylated JNK **(C)**, p38 **(D)**, and ERK **(E)** after 10 μM Cadmium treatment for 4 h in FMO 1–5 OE HEK293A cells [lane 8–12 in **(B)**] compared to empty vector control HEK293A cells [lane 7 in **(B)**]. **(F)** SAPK levels and phosphorylation including JNK, p38, and ERK in FMO1–5 OE HepG2 cells and empty vector control cells after 10 μM Cadmium treatment for 4 h. Quantitation of the phosphorylated JNK **(G)**, p38 **(H)**, and ERK **(I)** after 10 μM Cadmium treatment for 4 h in FMO1–5 OE HepG2 cells [lane 8–12 in **(F)**] compared to empty vector control HepG2 cells [lane 7 in **(F)**]. Western blot band intensities were quantified by Image J. The phosphorylated bands were normalized to the corresponding unphosphorylated bands of each SAPK, and then were compared to the empty vector pDEST control as fold changes. Data represent mean ± SEM. *p < 0.05, **p < 0.01, ***p < 0.001, and ****p < 0.0001.

To explore whether FMO-OE cells resist stress through SAPKs, we utilized cadmium as a tool, largely because it is the most consistent and reproducible stressor among those we have tested. First, we determined the time point and dosage under which SAPKs are activated by cadmium in our system. The results show that 4 and 6 h treatments with 10 μM cadmium activated SAPKs, including JNK, p38, and ERK (Figure 3A). We used 4 h as the time point to assess cadmium-induced kinase activities because we wanted the earliest time point before damage also becomes a factor. We then asked whether JNK, p38, and ERK activities are changed in FMO-overexpressing cells compared to control cells under stress. The results show that only JNK activity is consistently increased in all five FMO-OE cell lines of HEK293A (Figures 3B,C and Supplementary Figure 6A) and HepG2 (Figures 3F,G and Supplementary Figure 6B) under cadmium stress. p38 activity is significantly increased in HepG2 FMO1-OE cells (Figure 3H) but not in other FMO-OE cells (Figures 3D,H). ERK activity is largely not changed in all FMO-OE cells (Figures 3E,I). These data are consistent with JNK better responding and exerting protective mechanisms of cell survival when FMOs are overexpressed. Since we have shown that FMO-OE cells have similar stress resistance profiles to the fibroblasts from long-lived mice, the activation of JNKs in the FMO-OE cells is reminiscent of previous findings showing that increased JNK activity leads to increase tolerance for oxidative stress and increases lifespan dramatically in both *C. elegans* and *Drosophila* (Wang et al., 2003; Oh et al., 2005).

### FMO Expression Modulates the Balance Between Mitochondrial Respiration and Glycolysis

**FIGURE 4:**
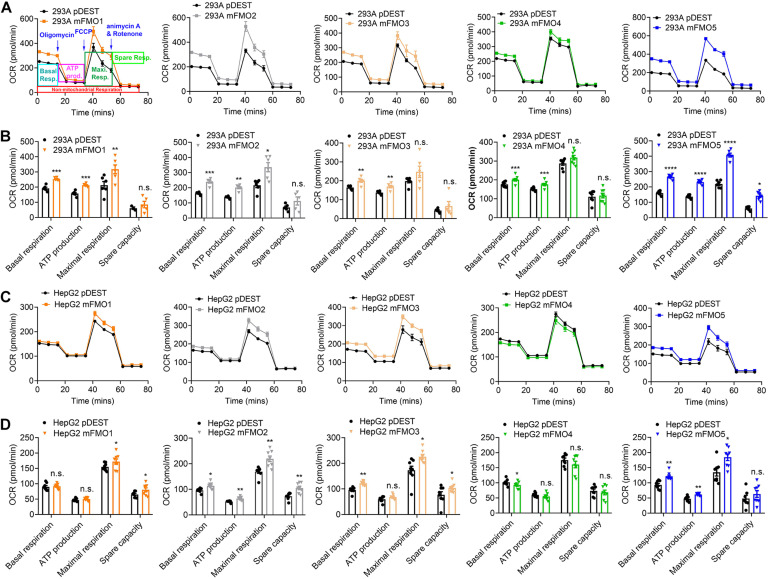
FMO expression increases mitochondrial respiration. **(A)** Mitochondrial respiration measured by OCR (oxygen consumption rate) in FMO1–5 OE HEK293A cells and empty vector control cells. Mitochondrial respiration chain complex inhibitors Oligomycin, Carbonyl cyanide-4 (trifluoromethoxy) phenylhydrazone (FCCP), and rotenone/antimycin A were injected stepwise as indicated. As denoted in the first panel, basal respiration, ATP production, maximal respiration, and spare respiration can be calculated by the OCR level changes in response to the inhibitor injections. **(B)** Basal respiration, ATP production, maximal respiration, and spare capacity in FMO1–5 OE HEK293A cells and empty vector control cells. **(C)** Mitochondrial respiration in FMO1–5 OE HepG2 cells. **(D)** Basal respiration, ATP production, maximal respiration, and spare capacity in FMO1–5 OE HepG2 cells. Data represent mean ± SEM. *p < 0.05, **p < 0.01, ***p < 0.001, and ****p < 0.0001.

**FIGURE 5:**
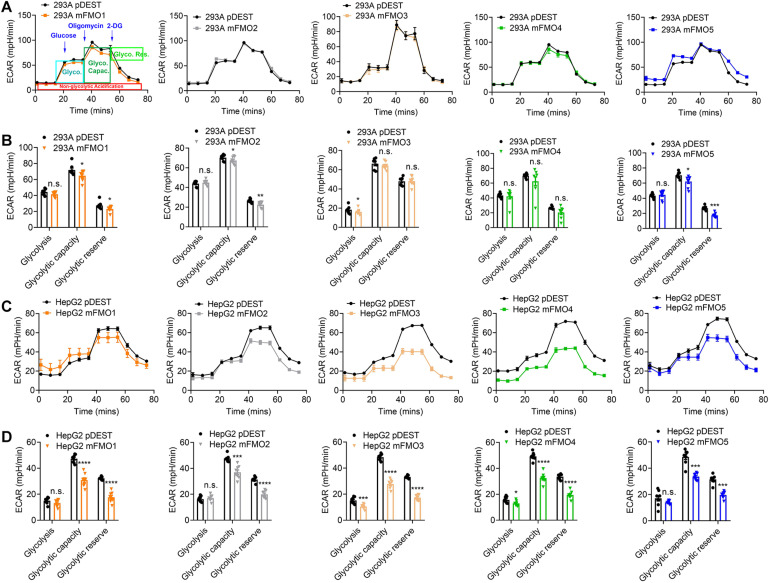
FMO overexpression decreases overall glycolytic activity. **(A)** Overall glycolytic activity measured by ECAR (extracellular acidification rates) in HEK293A FMO1–5 OE cells and empty vector control cells. Sequential injections of glucose, oligomycin, and 2-DG were applied over time as indicated. As denoted in the first panel, glycolysis, glycolytic capacity, and glycolytic reserve can be calculated by the ECAR level changes in response to individual injections. **(B)** Glycolysis, glycolytic capacity, and glycolytic reserve in HEK293A FMO1–5 OE cells and empty vector control cells. **(C)** Overall glycolytic activity in FMO1–5 OE HepG2 cells. **(D)** Glycolysis, glycolytic capacity, and glycolytic reserve in FMO1–5 OE HepG2 cells and empty vector control cells. Data represent mean ± SEM. *p < 0.05, **p < 0.01, ***p < 0.001, and ****p < 0.0001.

Previous reports using FMO knockout mice suggest that mouse FMOs play physiological roles in endogenous metabolism (Veeravalli et al., 2014; Gonzalez Malagon et al., 2015; Shih et al., 2015; Warrier et al., 2015), including decreased expression of glycolytic enzymes in the liver of FMO5 KO mice (Gonzalez Malagon et al., 2015). However, implications are complicated due to the tissue specific, developmental and sex-differential expression of the different FMOs. In our stable FMO-OE cell lines, we can assess metabolic changes without the *in vivo* tissue-specific complications. Mitochondrial respiration and glycolysis are the two major energy producing pathways of the cell. Mitochondrial respiration can be roughly measured by the oxygen consumption rate (OCR) for ATP production. During glycolysis, glucose is broken down to lactic acid, and protons produced are exported to the extracellular media and can be measured as extracellular acidification rate (ECAR). Using the Seahorse XFe96 Analyzer, we tested the mitochondrial respiration and glycolytic activities of FMO-OE and control empty vector cell lines. As shown in Figure 4A, mitochondrial respiration is increased in all HEK293A FMO-OE cells. Basal respiration, ATP production, and maximal respiration are all increased in these kidney FMO-OE cells (Figure 4B). Similarly, mitochondrial respiration is increased in FMO1, FMO2, FMO3, and FMO5 but not FMO4-OE HepG2 cells (Figure 4C). Maximal respiration is significantly improved in FMO1, FMO2, FMO3, and FMO5-OE HepG2 cells (Figure 4D). FMO2, FMO3, and FMO5 increase basal mitochondrial respiration (Figure 4D). ATP production is increased by FMO2 and FMO5 in HepG2 cells (Figure 4D). These results suggest that oxygen consumption through mitochondrial respiration is increased by FMO overexpression. In agreement with an increase in respiration, overall glycolytic activity as measured by ECAR shows a decreasing trend in HEK293A FMO-OE cells (Figures 5A,B). As quantified in Figure 5B, FMO3 significantly decreases glycolysis. Glycolytic capacity and glycolytic reserve are significantly decreased in FMO1, FMO2, and FMO5-OE HEK293A cells, while FMO3 and FMO4 show an insignificant decrease in glycolytic capacity and reserve (Figure 5B). In HepG2 cells, glycolytic activity is significantly decreased in all five HepG2 FMO-OE cells compared with vector controls (Figure 5C). As quantified in Figure 5D, glycolysis is decreased most significantly by FMO3 or FMO4 overexpression. Glycolytic capacity and glycolytic reserve are both significantly decreased in all five FMO-OE HepG2 cell lines (Figure 5D). These data are consistent with overexpression of FMOs shifting energy production to mitochondrial metabolism and away from carbohydrate metabolism.

### FMOs Regulate Essential Amino Acid, Carbohydrate, and Energetic Pathways

**FIGURE 6:**
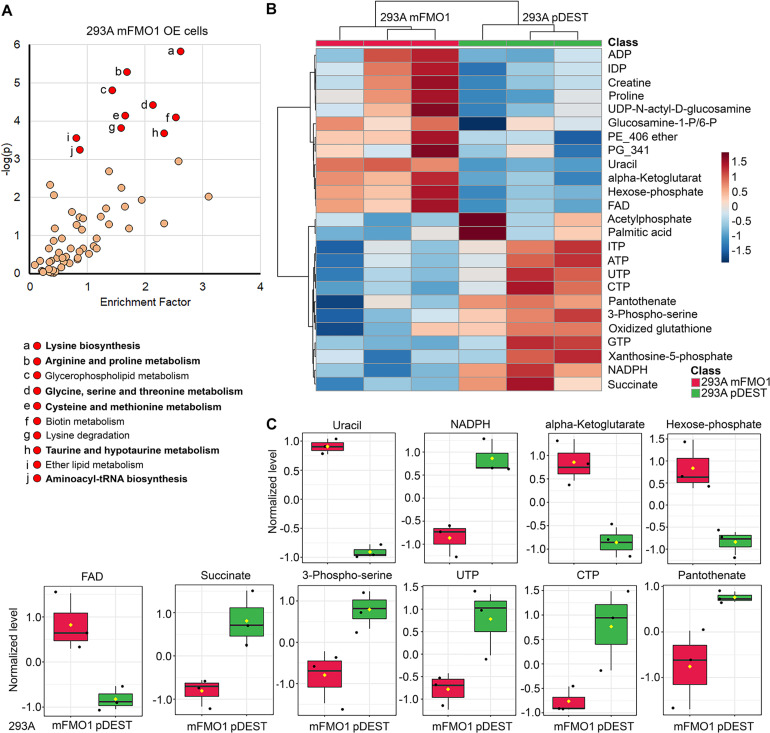
FMOs regulate amino acid and energetic metabolic pathways. Untargeted metabolomics in FMO1–5 OE cells (**A**, see also Supplementary Figures 7–15) compared to empty vector control cells, and the significantly regulated metabolic pathways that are enriched. The abundance analyses of metabolites in central carbon metabolism in FMO1–5 OE cells compared to empty vector control cells (**B,C**, see also Supplementary Figures 16–24). **(A)** Metabolic pathways regulated by FMO1 are plotted by the enrichment factor (obtained by dividing “significant hits” by “expected hits” for each pathway) on the *x*-axis and –log of the *p*-value on the *y*-axis. Red indicates significantly changed pathways with *p* < 0.001. Shared significantly regulated metabolic pathways by more than three FMOs in FMO1–5 are indicated in bold text, including amino acid metabolism (Glycine, serine, and threonine metabolism; Cyanoamino acid metabolism; Aminoacyl-tRNA biosynthesis; Selenoamino acid metabolism; Taurine and hypotaurine metabolism; Cysteine and methionine metabolism; Arginine and proline metabolism; Lysine biosynthesis; Alanine, aspartate, and glutamate metabolism) and metabolism of cofactors and vitamins (Pantothenate and CoA biosynthesis; Vitamin B6 metabolism) (see also Supplementary Figures 7–15). **(B)** The levels of the top 25 changed metabolites between FMO1-OE cells and empty vector control cells are shown in heat map. **(C)** The levels of metabolites significantly regulated by FMO1 are shown in FMO1-OE cells and empty vector control cells.

To investigate perturbations in metabolism in the stably transfected cell lines, we employed untargeted metabolomics analysis to our FMO-OE cell lines and compared them to empty vector control cell lines. The results show differences in fundamental metabolism pathways, including amino acid, carbohydrate, and energetic pathways (Figure 6A and Supplementary Figures 7–15). Through human KEGG (Kyoto Encyclopedia of Genes and Genomes) pathway analyses, we find that there are no clear discriminations between FMOs, and the metabolic pathways are significantly shared by almost all FMO-OE cells. From the shared metabolic pathways, amino acid metabolism, and protein biosynthesis are major pathways modulated by FMOs. “Glycine, serine, and threonine metabolism; Cyanoamino acid metabolism; Aminoacyl-tRNA biosynthesis; Selenoamino acid metabolism; Taurine and hypotaurine metabolism; Cysteine and methionine metabolism; Arginine and proline metabolism; Lysine biosynthesis; Alanine, aspartate, and glutamate metabolism; Pantothenate and CoA biosynthesis; Vitamin B6 metabolism” are the 11 pathways that are most significantly modified and shared by three or more FMOs in FMO1–5 (Highlighted by bold text in Figure 6A and Supplementary Figures 7–15). The enrichment of metabolites and the significance of the top pathways in HEK293A FMO1-OE cells are shown in Figure 6A as representative results. The enriched metabolic pathways in HEK293A FMO1, FMO2, FMO3, FMO4, and FMO5-OE cells are shown in Supplementary Figures 7–10 and Table 3.

**TABLE 3:** FMOs regulate amino acid, carbohydrate, and energetic metabolic pathways.

	293A	HepG2
mFMO1	Lysine biosynthesis	Cyanoamino acid metabolism
	Arginine and proline metabolism	Pantothenate and CoA biosynthesis
	Glycerophospholipid metabolism	Pentose phosphate pathway
	Glycine, serine, and threonine metabolism	Selenoamino acid metabolism
	Cysteine and methionine metabolism	Vitamin B6 metabolism
mFMO2	Alanine, aspartate, and glutamate metabolism	Vitamin B6 metabolism
	Cyanoamino acid metabolism	Valine, leucine, and isoleucine biosynthesis
	Lysine biosynthesis	Aminoacyl-tRNA biosynthesis
	Pantothenate and CoA biosynthesis	Pentose phosphate pathway
	Vitamin B6 metabolism	Biotin metabolism
mFMO3	Pyrimidine metabolism	Alanine, aspartate and glutamate metabolism
	Synthesis and degradation of ketone bodies	Arginine and proline metabolism
	Glycine, serine, and threonine metabolism	Pantothenate and CoA biosynthesis
	Alanine, aspartate, and glutamate metabolism	Butanoate metabolism
	Taurine and hypotaurine metabolism	Cyanoamino acid metabolism
mFMO4	Ascorbate and aldarate metabolism	Ether lipid metabolism
	Vitamin B6 metabolism	Glycerophospholipid metabolism
	Porphyrin and chlorophyll metabolism	D-Glutamine and D-glutamate metabolism
	Alanine, aspartate, and glutamate metabolism	Arachidonic acid metabolism
	Butanoate metabolism	Citrate cycle (TCA cycle)
mFMO5	Glycine, serine, and threonine metabolism	Methane metabolism
	Cyanoamino acid metabolism	Cyanoamino acid metabolism
	Pantothenate and CoA biosynthesis	Glutathione metabolism
	Aminoacyl-tRNA biosynthesis	Pyrimidine metabolism
	Sulfur metabolism	Glycine, serine, and threonine metabolism

*The top five metabolic pathways of FMO1–5 in HEK293A and HepG2 cells. Untargeted metabolomics in FMO1–5 OE cells compared to empty vector control cells, and the significantly regulated metabolic pathways that are enriched.*

The metabolic pathways significantly changed by FMO1–5 in HepG2 cells are shown in Supplementary Figures 11–15 and Table 3. Taurine and hypotaurine metabolism is detected as modified by FMO1, FMO3 and FMO5 in HEK293A cells (Figure 6A and Supplementary Figures 8, 10 and Supplementary Table 1). This is consistent with recent findings that FMO1 is a key enzyme for taurine biosynthesis through catalyzing the S-oxygenation of hypotaurine (Veeravalli et al., 2020). In addition to the common pathways shared by almost all FMOs, there are several pathways in the categories of carbohydrate, energy, lipid, and vitamin metabolisms shared by multiple FMOs (Supplementary Table 1). Interestingly, and consistently with our findings from seahorse metabolic measurements in Figures 4, 5 that FMOs shift energy production to mitochondrial respiration from glycolysis, pathways in carbohydrate metabolism, and energy metabolism are significantly differentially regulated in FMO-OE cells (Supplementary Table 1). Carbohydrate metabolism pathways, including the pentose phosphate pathway (by FMO1), TCA cycle (by FMO3), ascorbate and aldarate metabolism (by FMO4), and butanoate metabolism (by FMO3 and FMO4), are all significantly modified (Supplementary Table 1). Energy metabolism pathways, including methane metabolism (by FMO3 and FMO5), sulfur metabolism (by FMO5), and nitrogen metabolism (by FMO5), are also altered in these cell lines (Supplementary Table 1). Metabolism of cofactors and vitamins is another aspect differentially regulated in FMO-OE cells, with significant changes in vitamin B6 metabolism (by FMO1, FMO2, FMO3, and FMO4), pantothenate and CoA biosynthesis (by FMO1, FMO2, FMO3, and FMO5), biotin metabolism (by FMO1 and FMO3), nicotinate and nicotinamide metabolism (by FMO3), and porphyrin and chlorophyll metabolism (by FMO4 and FMO5) (Supplementary Table 1). Lipid metabolism is also differentially regulated in FMO-OE cells, including glycerophospholipid metabolism (by FMO1 and FMO4), ether lipid metabolism (by FMO1 and FMO4), alpha-Linolenic acid metabolism (by FMO3), and the synthesis and degradation of ketone bodies pathway (by FMO3) (Supplementary Table 1). Together, these results from untargeted metabolomics analyses suggest that FMO expression significantly changes endogenous metabolism and regulates amino acid, carbohydrate, energy, vitamin, and lipid metabolism.

### Central Carbon Metabolism Is Regulated by FMOs

Since carbon metabolism is a common feature of the metabolic pathways regulated by FMOs (Figure 6A and Supplementary Figures 7–15), and mitochondrial respiration and glycolytic activities are also modulated (Figures 4, 5), we next asked whether FMOs regulate central carbon metabolism. We targeted the abundance of 102 central carbon metabolites in FMO-OE cells compared to empty vector control cells. As shown by Principle Component Analysis (PCA), the clusters of three replicates of FMO-overexpressing or empty vector control HEK293A cells are distinctly separated for FMO1 (Supplementary Figure 16A), FMO2 (Supplementary Figure 16B), FMO3 (Supplementary Figure 17A), FMO4 (Supplementary Figure 18A), and FMO5 (Supplementary Figure 19A), suggesting FMO overexpression results in dramatic changes in central carbon metabolism in HEK293A cells. In HepG2 cells, the differences in central carbon metabolites regulations between FMO-OE cells and empty vector control cells are not as distinct as in HEK293A cells, with overlapping clusters between FMO1, FMO3, or FMO5 OE cells and empty vector control cells (Supplementary Figures 20A–24A). The levels of the top 25 changed metabolites between FMO-OE cells and empty vector control cells are shown in a heat map for FMO1 (Figure 6B), FMO2 (Supplementary Figure 16C), FMO3 (Supplementary Figure 17B), FMO4 (Supplementary Figure 18B), and FMO5 (Supplementary Figure 19B) in HEK293A cells, and FMO1–5 OE in HepG2 cells (Supplementary Figures 20B–24B). Among the individual metabolites that are significantly regulated by FMOs shown in Figure 6C and Supplementary Figures 16D, 17C–24C and Supplementary Tables 2–11, amino acids, including arginine (Supplementary Figures 17C, 19C, 22C), glutamine (Supplementary Figures 21C, 24C), methionine (Supplementary Figures 21C, 23C), tryptophan (Supplementary Figures 21C, 23C), citrulline (Supplementary Figures 21C, 23C), and phenylalanine (Supplementary Figures 21C, 23C, 24C), are downregulated by FMOs, consistent with the results from untargeted metabolomics that amino acid metabolism is the most significantly regulated pathway. The metabolites in energy production metabolism, including FAD, NADP, NADPH, ADP, AMP, ATP, phosphocreatine, succinate, and glycerol-3-phosphate, are also significantly regulated by multiple FMOs, with upregulation of FAD (Figure 6C and Supplementary Figures 18C, 19C), NADP (Supplementary Figure 18C), ADP (Supplementary Figure 24C) and AMP (Supplementary Figure 18C) and downregulation of NADPH (Figure 6C and Supplementary Figure 19C), ATP (Supplementary Figure 19C), phosphocreatine (Supplementary Figures 19C–23C), succinate (Figure 6C and Supplementary Figure 24C), and glycerol-3-phosphate (Supplementary Figures 17C, 18C, 20C, 21C). To summarize, our untargeted and targeted metabolomics analyses demonstrate that FMO expression significantly modifies endogenous metabolism, primarily in amino acid and energy metabolism.

## Discussion

In this study, we evaluated the possible benefits of mammalian FMOs by exploring and comparing the stress resistance and metabolic impacts of all FMOs in the same platform. Our results demonstrate that FMOs may all play similar endogenous functions to improve resistance to a broad range of stressors. Our metabolic analyses demonstrate that FMOs balance the cellular energy producing pathways between mitochondrial respiration and glycolysis, with enhanced activity of mitochondrial respiration and depressed glycolytic activity. Through metabolomics analyses, we reveal that amino acid, carbohydrate, and energy metabolism are the major regulated pathways by FMOs, which is further confirmed by the significant changes in the central carbon metabolites of amino acids and energy metabolism cofactors. Our study for the first time elucidates the cellular functions and metabolic pathways regulated by all five FMOs in a cell culture system.

Mice are a useful model to study FMOs because they allow the examination of mammalian physiology in respect to the endogenous functions of FMOs, with all the intricacy and interactions of an intact animal. However, the tissue-specific, developmental, and gender differential expressions of different FMOs complicate the interpretation of results in mammals. For example, FMO1 is not expressed after birth in the human liver; FMO4 expression is low in both human and mouse; and FMO3 is not normally expressed in male mice liver but is highly expressed in female mice liver. The concern of gender differential expression of FMO3 also applies to mouse liver cells as it matters which gender the hepatocytes were originally isolated from Houseman et al. (2015). Taking these potential confounders into consideration, we focus here on the endogenous role of FMOs by overexpressing FMOs in mammalian cells. By overexpressing mouse FMO1, FMO2, FMO3, FMO4, or FMO5 in both liver and kidney cells, we successfully examined the cellular stress resistance and metabolic changes resulting from this expression. This allowed us to compare whether there were significant and/or differential effects of different FMOs in the same platform, without the complication of tissue-specific, developmental, and gender differential expression of different FMOs *in vivo*.

Stress resistance assays show that all five FMOs help resist a wide range of stressors, including paraquat, cadmium, rotenone, arsenite, and UV-radiation, with variations in the extent of stress resistance by different FMOs in HEK293A and HepG2 cells (Figures 1, 2 and Supplementary Figures 3–5). Due to the tissue-specific expression of different FMOs as reported, the endogenous basal expression levels of different FMOs are different in the two cell types (HEK293A kidney cells and HepG2 liver cells) that we used (Figure 1C). Consistent with the report that human liver cells express high level of FMO5 (Zhang and Cashman, 2006), our data also show that endogenous FMO5 levels are lower in HEK293A than HepG2 cells (Figure 1C). This difference between these two cell types may contribute to some differences in their response to the same type of stress. For example, FMO5-OE HEK293A cells show a dramatic increase in stress resistance to cadmium and arsenite when compared to the control HEK293A cells where almost no endogenous FMO5 was detected (Figures 1C,F,2A and Tables 1C, 2A). Conversely, the increase in stress resistance of FMO5-OE HepG2 cells to cadmium and arsenite is not as dramatic as in HEK293A cells, plausibly due to high level of endogenous FMO5 in HepG2 control cells (Figures 1C,F,2B and Table 1D). All five FMOs increased stress resistance when overexpressed, regardless of whether they are normally expressed in the cell-type, consistent with FMOs sharing a common and redundant function in stress resistance. It is also interesting that FMO-OE cell lines resist such a wide range of stressors, resembling the stress resistance profile of fibroblasts from long-lived mice.

Further exploring the underlying mechanism of stress resistance through FMO expression, our results in Figure 3 show that JNK activity is upregulated in FMO-OE cell lines under cadmium stress. This result suggests that SAPKs, which are responsive to multiple stresses, are more readily activated in FMO-OE cells and could lead to their improved stress resistance. Interestingly, SAPK activity is reported to closely correlate with longevity. JNK acts in parallel with the insulin-like signaling pathway and directly phosphorylates DAF-16/FOXO, the forkhead transcription factor, leading to lifespan extension in *C. elegans* (Oh et al., 2005). Another SAPK, p38, also acts in the insulin-like signaling pathway for lifespan extension in *C. elegans* (Troemel et al., 2006). ERK also promotes longevity through two pro-longevity transcriptional factors, SKN-1 and DAF-16, in *C. elegans* (Okuyama et al., 2010). Similar to FMOs, increased levels of ERK activity are also found in long-lived mice models, including Snell dwarf mice and caloric restricted mice (Ikeyama et al., 2002; Madsen et al., 2004). Future studies will need to further examine whether JNK loss-of-function mutations diminish the increased stress resistance in mammalian FMO-OE cells or if JNK is necessary for the lifespan extension by *fmo-2* overexpression in *C. elegans*. It is also not clear how JNK activity is upregulated by FMO-overexpression and what substrates and metabolites produced by FMOs might lead to the activation of JNK.

The primary mechanism of FMO activity is to oxygenate substrates containing soft-nucleophiles, such as nitrogen and sulfur. Our metabolic data do not test what the key endogenous substrates may be, but they will be of great interest based on their potential benefits. Our untargeted (Figure 6A and Supplementary Figures 7–15) and targeted metabolomics (Figures 6B,C and Supplementary Figures 16–24) analyses show that FMO expression significantly changes endogenous metabolism. The most regulated pathways of amino acid, carbohydrate, and energy metabolism indicate that FMOs may regulate the fundamental metabolism of the cell. Additional work will be necessary to identify the endogenous FMO substrates that are responsible for direct and/or indirect changes to broader metabolic pathways. It will be interesting to test possible FMO substrates from the set of metabolites identified in our untargeted and targeted metabolomics, using stress resistance in FMO-OE cells or lifespan extension by *fmo-2* in worms as a readout.

Together, despite tissue-specific, developmental and gender differential expression in mice and humans, when overexpressed individually in kidney or liver cells, all five FMOs provide similar stress resistance (Figures 1,2 and Supplementary Figures 3–5) and share many primary pathways and metabolic changes (Figure 6 and Supplementary Figures 7–24 and Supplementary Tables 1–11). This redundancy and compensatory role of FMOs are reasonable because: (1) the recently resolved crystal structures of reconstructed ancestral FMO2, FMO3–6, and FMO5 showed that FMOs have a high degree of similarity in structure and also catalytic cavity (Nicoll et al., 2019); (2) this is in agreement with previous findings in mice showing that, although expressed in different tissues, FMO1, 2, and 4 knockout mice and FMO5 knockout mice display similar lean phenotypes and higher whole-body energy expenditure, while at different ages (Veeravalli et al., 2014; Gonzalez Malagon et al., 2015); and (3) resistance to multiple stressors conferred by overexpression of all five FMOs may indicate that FMOs share metabolic pathways to confer stress resistance. An interesting example of this is *FMO2*, which is non-functional in most humans due to a nonsense mutation producing a truncated inactive protein (Dolphin et al., 1998; Whetstine et al., 2000; Veeramah et al., 2008). However, in our FMO2-OE cell lines where a full-length FMO2 was expressed, FMO2-OE has a similar stress resistance and metabolic profile as with other FMO-OE cells. This further indicates the redundancy and compensatory roles of FMOs. In line with this, it will be interesting to test other mammalian FMOs. Human *FMO6–11* exhibit characteristics of pseudogenes; however, the highly homologous mouse *Fmo6*, *Fmo9*, *Fmo12*, and *Fmo13* are predicted to produce polypeptides and thus may not be pseudogenes in mice (Hernandez et al., 2004). Further experimental analyses are needed to characterize them as functional FMOs; therefore, while we did not include these mouse FMOs in this study, they may be of interest. Overall, after years of research focusing primarily on the classic role of FMOs in metabolizing environmental chemicals and therapeutic drugs, we have shown new roles for FMOs in regulating cellular stress resistance and metabolism, which provides insights into an exciting new area of FMO research.

## Experimental Procedures

### *C. elegans* Strain Maintenance and Paraquat Stress Assay

Standard procedures for *C. elegans* strain maintenance and handling were used. In detail, Wild-Type and FMO-2 OE strains were kept at 20°C in a temperature-controlled incubator on NGM (Nematode Growth Medium) with the food source of *Escherichia coli* OP50 seeded on top of NGM. For the paraquat stress assay, both strains were synchronized from eggs and grown to the fourth larvae stage (L4). For each replicate, 30 L4 worms of wild-type or FMO-2 OE were put on OP50-seeded NGM containing 5 mM paraquat. Three replicates of the same condition were included. Experimental animals were then scored every day and counted as dead when not responding to prodding under a dissection microscope.

### Chemicals, Antibodies, Plasmids and Primers

Cadmium was purchased from HAMPTON RESEARCH (HR2-715, 1.0 M), and diluted to 10 mg/ml as stock solution. Paraquat (Methyl viologen dichloride hydrate) was purchased from Sigma (856177-1g), and dissolved fresh each day as a stock solution of 1 M. Sodium Arsenite Solution was purchased from HACH (104732-100 mL, 5 g/L). Rotenone was purchased from Sigma (R8857), and dissolved in DMSO as a stock solution of 100 mM. The following antibodies were used: mouse monoclonal antibody against FMO2 (Proteintech 67019-1-Ig) and polyclonal antibodies against FMO1 (Invitrogen PA5-95285), FMO3 (Abcam ab126711), FMO4 (Invitrogen PA5-79276), FMO5 (Proteintech 13699-1-AP), JNKs (Cell Signaling Technology 9252), phospho-SAPK/JNK (Thr183/Tyr185) (Cell Signaling Technology 9251), ERK1/2 (Cell Signaling Technology 4695), phospho-p44/42 MAPK (Erk1/2) (Thr202/Tyr204) (Cell Signaling Technology 9101), p38 (Cell Signaling Technology 9212), phospho-p38 MAPK (Thr180/Tyr182) (Cell Signaling Technology 9211), and GAPDH (Cell Signaling Technology 2118).

Mouse FMO1 (Accession No. U87456), FMO2 (Accession No. AF184981), and FMO3 (Accession No. NM_008030) were cloned from mouse liver cDNA (ZYAGEN) and inserted into Gateway^TM^ pcDNA^TM^-DEST47 Vector (Invitrogen 12281010). Mouse FMO5 (Accession No. U90535) cDNA was cloned from mouse FMO5 ORF clone purchased from ORIGENE and then inserted into Gateway^TM^ pcDNA^TM^-DEST47 Vector (Invitrogen 12281010). Mouse FMO4 (Accession No. NM_144878) in pcDNA 3.1 (+) was purchased from GenScript. All constructs were confirmed by DNA sequencing. These FMOs constructs were then transfected into HEK293A or HepG2 cells using TransIT^®^-LT1 (Mirus MIR2300).

### Quantitative Real-Time PCR

Total RNA was extracted using RNeasy Mini Kit (QIAGEN) and 1 μg RNA was reverse transcribed to cDNA by Maxima^TM^ H Minus cDNA Synthesis Master Mix (Invitrogen). qPR-PCR was performed with 1 μg of cDNA and SYBR^TM^ Green PCR Master (Applied Biosystems). β-2-microglobulin was used as a housekeeping gene control for FMO mRNA level normalization. FMO mRNA expression in the FMO-OE cell line was compared to the empty vector pDEST control as fold change. The following qPCR primers were used to confirm the overexpression levels of FMOs in the stable FMO-OE cell lines: mFMO1 forward primer (5′-ACAGCCGACAGTATAAACATCCA-3′) and reverse primer (5′-CCCTCCAGTAGTGCTGAGGAACA-3′); mFMO2 forward primer (5′- AGTGGCCTAATCTCTCTGAAGT-3′) and reverse primer (5′-CATCGGGAAGTCACTGAAACAG-3′); mFMO3 forward primer (5′-ACTGGTGGTACACAAGGCAG-3′) and reverse primer (5′-ATGGTCCCATCCTCAAACACA-3′); mFMO4 forward primer (5′-GATTGGAGCTGGCGTAAGT G-3′) and reverse primer (5′-TGTCAGCAAACTTCCACAGTC-3′); mFMO5 forward primer (5′-GAGGGCTTGGAACCTGTCT G-3′) and reverse primer (5′-CACGGACTGGTAAATAC TGGC-3′).

### Cell Culture and Stress Resistance Assay

HEK293A and HepG2 cells were grown in Dulbecco’s modified Eagle’s medium (DMEM, GIBCO) supplemented with 10% fetal bovine serum, 100 U/ml penicillin, and 100 μg/ml streptomycin. 2 μg of FMO plasmid was transfected into HEK293A or HepG2 cells using TransIT^®^-LT1 Transfection Reagent (Mirus) according to the manufacturer’s instructions. 48 h after transfection, the cells were then cultured with G418 sulfate Geneticin^TM^ (Gibco10131035) at 500 μg/ml to obtain G418-resistant cell lines. The stably transfected cells were maintained in medium containing 500 μg/ml G418 throughout cultures. For stress resistance assay, cells were seeded to 96-well microplates with 25,000 trypsinized cells per well for HEK293A cells and 40,000 cells per well for HepG2 cells. Empty vector control cells and FMO-overexpressing cells were seeded in triplicate for each dose of stressor on the same plate. After 16–18 h overnight incubation in complete medium, the cells were incubated for 18–24 h in serum-free DMEM supplemented with 2% bovine serum albumin (BSA) as described previously (Murakami et al., 2003). For stress treatments, cells were exposed to indicated range of doses for 6 h (cadmium and paraquat stressors) or 24 h (rotenone and arsenite stressors) in 2% BSA supplemented DMEM, and then incubated in fresh 2% BSA supplemented DMEM without stressor for 18 h, followed by measurements of cell survival by Cell Proliferation Reagent WST-1 (Sigma 5015944001). For UV stress, the medium of the cells in the 96-well microplates were changed to 100 μL phosphate-buffered saline (PBS) and then irradiated with UV light for indicated range of intensity (μJ/cm^2^). After radiation, cells were incubated in 2% BSA supplemented DMEM for 18 h and followed by WST-1 cell survival measurements.

### Measurement of Mitochondrial Respiration and Glycolysis

Cells were seeded to Seahorse XF96 Cell Culture Microplates with 40,000 trypsinized cells per well for HEK293A cells or HepG2 cells. The mitochondrial respiration rate was measured by oxygen consumption rate (OCR) detected by the Agilent Seahorse XFe96 Analyzer at 37°C. Glycolysis was measured by protons extruded into the extracellular media that can be detected by the Agilent Seahorse XFe96 Analyzer as extracellular acidification rates (ECAR) at 37°C. For mitochondrial respiration measurements, the analyzer stepwise injects oligomycin (2 μM final concentration per well), Carbonyl cyanide-4 (trifluoromethoxy) phenylhydrazone (FCCP) (1 μM final concentration per well), and rotenone/antimycin A (0.5 μM final concentration per well). The first injection of oligomycin, the ATP synthase inhibitor, decreases OCR correlated to mitochondrial respiration associated with ATP production. The second injection of FCCP, an uncoupling agent that collapses the proton gradient and the mitochondrial membrane potential, frees the electron flow through the electron transport chain (ETC) so that the oxygen is maximally consumed by complex IV, generating maximal respiration. The difference between basal respiration and maximal respiration defines the spare respiratory capacity responding to energy demand. The final injection is mixture of rotenone, a complex I inhibitor, and antimycin A, a complex III inhibitor, which completely inhibits mitochondrial respiration and allows the calculation of non-mitochondrial respiration from processes outside the mitochondria. In the glycolysis measurements, the analyzer stepwise injects glucose (10 mM final concentration per well), oligomycin (2 μM final concentration per well), and 2-deoxy-glucose (2-DG) (50 mM final concentration per well). The cells were cultured in XF Glycolysis stress test assay medium without glucose before the injections. The first injection of glucose catabolizes it through glycolysis to pyruvate, producing ATP, NADH, water, and protons. The second injection of oligomycin, an ATP synthase inhibitor, inhibits mitochondrial ATP production and shifts the energy production to glycolysis, producing the cellular maximum glycolytic capacity. The difference between glycolysis and glycolytic capacity indicates the glycolytic reserve. The final injection of 2-DG, a glucose analog, inhibits the first step of glycolysis through competitive binding to glucose hexokinase, resulting collapse of ECAR produced in the glycolysis. The detailed procedure followed the manufacturer’s instructions in XFe96 Training Manual^1^.

### Metabolomics Assay

Sample preparation was performed by rinsing the cells with cold 150 mM ammonium acetate for less than 5 s. After removing the rinse buffer, cells were snap frozen directly in the plates by pouring liquid nitrogen into the plates, and then stored in −80°C until extraction. Metabolites were then identified by mass spectrometry. Specifically, metabolites were extracted from the cells by addition of 500 μL of ice-cold 9:1 methanol: chloroform. The resulting suspension was immediately transferred to tubes and probe sonicated for 10 s with a Branson 450 Sonicator. The resulting homogenates supernatant was then transferred to autosampler vials for analysis. Hydrophilic interaction liquid chromatography-electrospray ionization mass spectrometry (HILIC-LC-ESI -MS) analysis was performed in negative ion mode using an Agilent 1200 LC system coupled to an Agilent 6220 time-of-flight mass spectrometer. For chromatography, the Phenomenex Luna NH2 column was used with dimensions of 150 mm × 1.0 mm ID, 0.07 mL/min flow rate, and 10 μL injection volume, with LC gradient and MS parameters as previously described (Thonusin et al., 2017). The resulting untargeted metabolomics data were analyzed using MetaboAnalyst 4.0^2^ in the MS Peaks to Paths module. The resulting targeted metabolomics data were analyzed using MetaboAnalyst 4.0 in the Statistical Analysis module with median normalization, log transformation and auto scaling adjustments.

### Calculation of LD_50_ and Statistical Analyses

Mean survival rate from triplicates was used to determine the LD_50_ for each biological replicate of FMO-OE or empty vector control cell line by “Non-linear Regression” and “Dose-response-inhibition” after normalization, using GraphPad Prism 8.0.0. Some of the survival curves in response to stressors did not reach a survival rate under 50% at the highest dose of the stressor. In this situation, LD50 cannot be accurately calculated, therefore, we have listed them as greater than the highest dose we tested in the summarizing tables and showed the dose-dependent survival curves in the figures. Student *t*-test analysis was used to calculate *p*-values for comparisons between FMOs and empty vector control in stress assays, western blots, and seahorse assays. Log-rank test was used to derive *p*-value for survival curves comparison.

## Data Availability Statement

The raw data supporting the conclusions of this article will be made available by the authors, without undue reservation.

## Author Contributions

SL conceived the research. SL and SH designed the experiments, analyzed the data, and wrote the manuscript. SH performed the experiments. MH performed the sequence alignments. CD cloned FMOs genes and constructed FMOs plasmids. CE performed the metabolomics assay. All authors contributed to the article and approved the submitted version.

## Conflict of Interest

The authors declare that the research was conducted in the absence of any commercial or financial relationships that could be construed as a potential conflict of interest.

## Footnotes

1 www.agilent.com

2 http://metaboanalyst.ca

## Supplementary Material

Supplementary Figure 1 Amino acid sequence alignment and sequence identities among reconstructed ancestral mammalian FMO5, *C. elegans* FMO-2, and mouse FMO1–5. **(A)** Full length amino acid alignment of reconstructed ancestral mammalian FMO5 with *C. elegans* and mouse FMOs. Eight essential residues in the catalytic active site were denoted as red arrow heads. **(B)** The percent identity among reconstructed ancestral mammalian FMO5, *C. elegans* FMO-2, and mouse FMO1–5 based on alignment using Clustal Omega.

Supplementary Figure 2 Expression of FMO1–5 in HEK293A and HepG2 cells. FMO1–5 mRNA levels in HEK293A FMO-OE cells **(A)** and HepG2 FMO-OE cells **(B)** were examined by quantitative PCR. Relative ratios of FMOs mRNA levels between FMO-OE and empty vector control pDEST cells are shown.

Supplementary Figure 3 FMOs improve stress resistance to oxidative stress in HEK 293A and HepG2 cells. **(A,B)** FMO1–4 OE and control cells survival curves on paraquat stress in HEK293A and HepG2. HEK293A cells **(A)** or HepG2 cells **(B)** stably expressing FMO1–4 or empty vector pDEST were subjected to indicated increasing doses of paraquat. **(C,D)** FMO1–4 OE and control cells survival curves on cadmium stress in HEK 293A and HepG2. HEK293A cells **(C)** or HepG2 cells **(D)** stably expressing FMO1–4 or empty vector pDEST were subjected to indicated increasing doses of cadmium. Data represent mean ± SEM. ^∗^*p* < 0.05, ^∗∗^*p* < 0.01, and ^∗∗∗^*p* < 0.001.

Supplementary Figure 4 FMOs improve stress resistance to broader stressors in mammalian cells. **(A)** LD_50_ values of FMO1, FMO3, or FMO4 OE and control cells on arsenite stress in HEK293A. HEK293A cells stably expressing FMO or empty vector pDEST were subjected to indicated increasing doses of arsenite. **(B,C)** FMO1-4 OE and control cells survival curves on UV-radiation in HEK 293A and HepG2. HEK293A cells **(B)** or HepG2 cells **(C)** stably expressing FMO1-4 or empty vector pDEST were subjected to indicated increasing UV-radiation. Data represent mean ± SEM. ^∗^*p* < 0.05, ^∗∗^*p* < 0.01, and ^∗∗∗^*p* < 0.001.

Supplementary Figure 5 FMOs improve stress resistance to rotenone in HEK 293A and HepG2 cells. FMO1–5 OE cells and control cells survival curves on rotenone stress in HEK293A and HepG2. HEK293A cells **(A)** or HepG2 cells **(B)** stably expressing FMO1–5 or empty vector pDEST were subjected to indicated increasing doses of rotenone. Data represent mean ± SEM. ^∗^*p* < 0.05, ^∗∗^*p* < 0.01, and ^∗∗∗^*p* < 0.001.

Supplementary Figure 6 JNK kinase activity is increased in FMO-overexpressing cells under cadmium-induced oxidative stress. SAPKs levels and phosphorylation including JNK, p38, and ERK in FMO1–5 OE HEK293A cells **(A)** or HepG2 cells **(B)** and empty vector control cells after 10 μM Cadmium treatment for 4 h.

Supplementary Figures 7–15 FMOs regulate amino acid, carbohydrate, and energetic metabolic pathways. Untargeted metabolomics in FMO1–5 OE cells compared to empty vector control cells, and the significantly regulated metabolic pathways are enriched (see also Figure 6A). **(A)** Metabolic pathways regulated by FMO1 (Supplementary Figure 7A), FMO2 (Supplementary Figure 7B), FMO3 (Supplementary Figure 8A), FMO4 (Supplementary Figure 9A) or FMO5 (Supplementary Figure 10A) in HEK293A cells and FMO1 (Supplementary Figure 11A), FMO2 (Supplementary Figure 12A), FMO3 (Supplementary Figure 13A), FMO4 (Supplementary Figure 14A), or FMO5 (Supplementary Figure 15A) in HepG2 cells are ranked according to the *p*-values from pathway enrichment analyses. Red indicates the most significantly changed pathways with *p* < 0.001. **(B)** Metabolic pathways regulated by FMO2 (Supplementary Figure 7C), FMO3 (Supplementary Figure 8B), FMO4 (Supplementary Figure 9B), or FMO5 (Supplementary Figure 10B) in HEK293A cells and FMO1 (Supplementary Figure 11B), FMO2 (Supplementary Figure 12B), FMO3 (Supplementary Figure 13B), FMO4 (Supplementary Figure 14B), or FMO5 (Supplementary Figure 15B) in HepG2 cells are plotted by the enrichment factor (obtained by dividing “significant hits” by “expected hits” for each pathway) on the *x*-axis and −log of the *p*-value on the *y*-axis. Red indicates significantly changed pathways with *p* < 0.001. Shared significantly regulated metabolic pathways by more than 3 FMOs in FMO1–5 are indicated by bold text.

Supplementary Figures 16–24 Central carbon metabolism is regulated by FMOs. The abundance analyses of metabolites in central carbon metabolism in FMO-OE cells compared to empty vector control cells (see also Figure 6). **(A)** Principle Component Analysis (PCA) plot of the clusters of three replicates of FMO1-OE (Supplementary Figure 16A), FMO2-OE (Supplementary Figure 16B), FMO3-OE (Supplementary Figure 17A), FMO4-OE (Supplementary Figure 18A), or FMO5-OE (Supplementary Figure 19A) and empty vector control of HEK293A cells and FMO1-OE (Supplementary Figure 20A), FMO2-OE (Supplementary Figure 21A), FMO3-OE (Supplementary Figure 22A), FMO4-OE (Supplementary Figure 23A), or FMO5-OE (Supplementary Figure 24A) and empty vector control of HepG2 cells. **(B)** The levels of the top 25 changed metabolites between FMO-OE cells and empty vector control cells are shown in heat map (Supplementary Figures 16C, 17B–24B). **(C)** The levels of metabolites significantly regulated by FMOs are compared between FMO-OE cells and empty vector control pDEST cells (Supplementary Figures 16D, 17C–24C).

Supplementary Table 1 Significant pathways in all FMOs in HEK293A and HepG2 cells from the untargeted metabolomics analyses. “Metabolism pathways” shows the significant pathways. “Categories of pathways” shows the category that the metabolic pathway is classified into according to KEGG (Kyoto Encyclopedia of Genes and Genomes) PATHWAY Database. Numbers shown under each FMO of HEK293A or HepG2 cells are the values of Fisher’s exact test (FET) which is used to assess the significance of pathways from MetaboAnalyst 4.0 in the MS Peaks to Paths module. Only pathways with a significant FET < 0.05 are shown in this table.

Supplementary Tables 2–11 Significantly regulated metabolites by FMOs from the targeted metabolomics analyses. The significantly changed metabolites between FMO1–5 OE and empty vector control in HEK293A cells (Supplementary Tables 2–6) and HepG2 cells (Supplementary Tables 7–11) are shown. “*p* value” shows the *p*-value of each metabolite from MetaboAnalyst 4.0 in the Statistical Analysis module by *t*-test with a threshold of 0.05.
