# Supplementary material for: Flavin-Containing Monooxygenases Are Conserved Regulators of Stress Resistance and Metabolism
Source: Front Cell Dev Biol. 2021 Feb 12;9:630188. doi: 10.3389/fcell.2021.630188 (PMC7907451; doi:10.3389/fcell.2021.630188)
Supplement: Supplementary Figure 1 — Amino acid sequence alignment and sequence identities among reconstructed ancestral mammalian FMO5, C. elegans FMO-2, and mouse FMO1–5. (A) Full length amino acid alignment of reconstructed ancestral mammalian FMO5 with C. elegans and mouse FMOs. Eight essential residues in the catalytic active site were denoted as red arrow heads. (B) The percent identity among reconstructed ancestral mammalian FMO5, C. elegans FMO-2, and mouse FMO1–5 based on alignment using Clustal Omega. [file Data_Sheet_1.PDF]

A

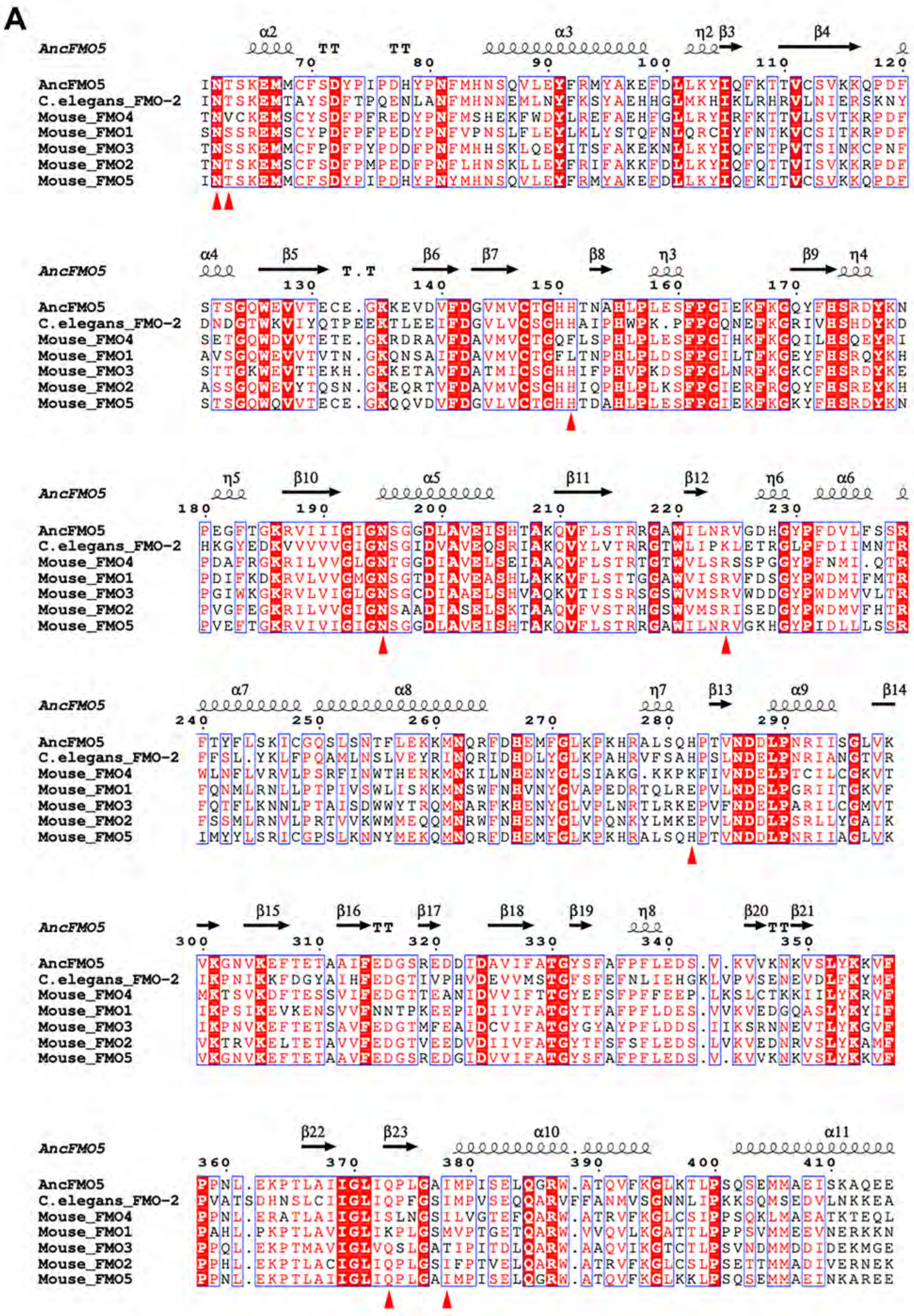

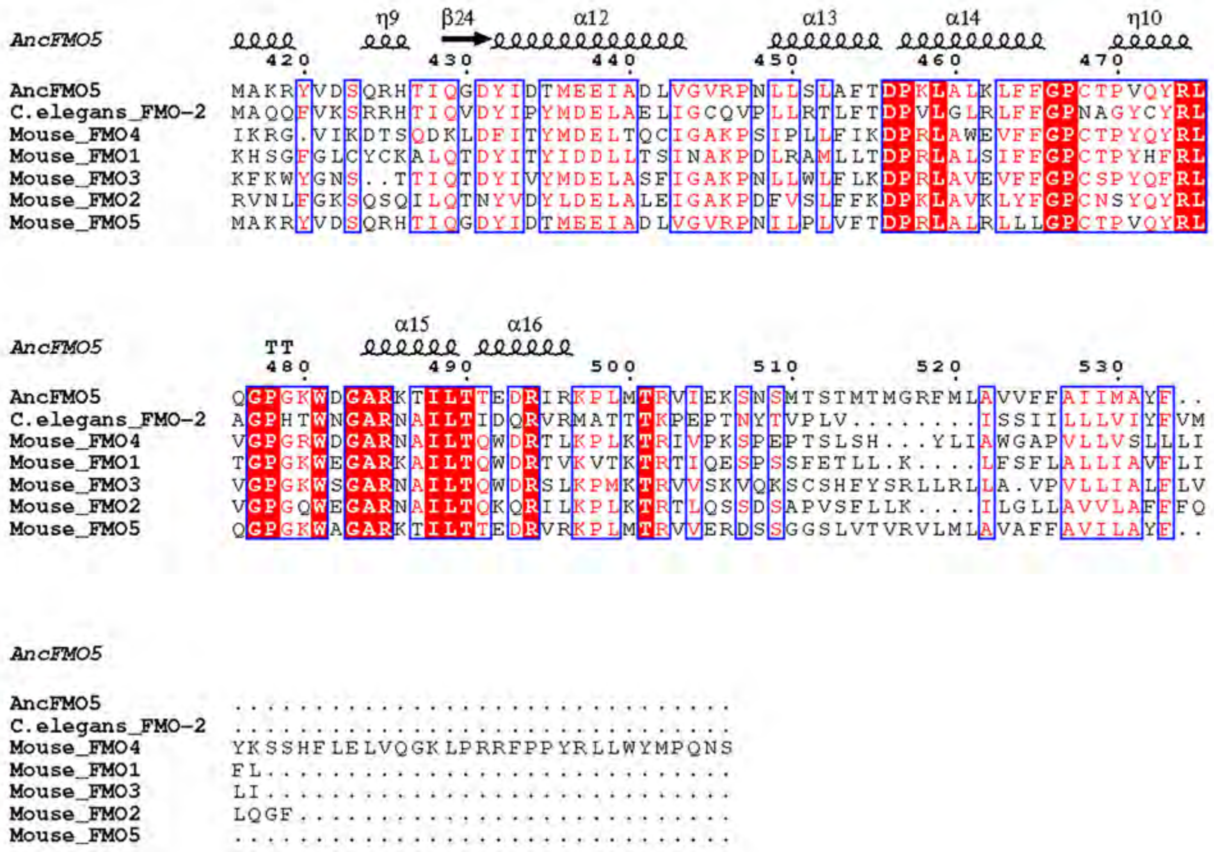

B

Percent Identity Matrix - created by Clustal2.1

1:	C.elegans_FMO-2	100.00	36.28	38.55	37.81	38.93	42.53	43.68
2:	Mouse_FMO4	36.28	100.00	49.62	50.57	52.83	48.86	50.19
3:	Mouse_FMO1	38.55	49.62	100.00	54.25	55.45	49.72	51.61
4:	Mouse_FMO3	37.81	50.57	54.25	100.00	56.42	54.06	55.01
5:	Mouse_FMO2	38.93	52.83	55.45	56.42	100.00	55.68	57.58
6:	Mouse_FMO5	42.53	48.86	49.72	54.06	55.68	100.00	88.37
7:	AncFMO5	43.68	50.19	51.61	55.01	57.58	88.37	100.00

Figure S1

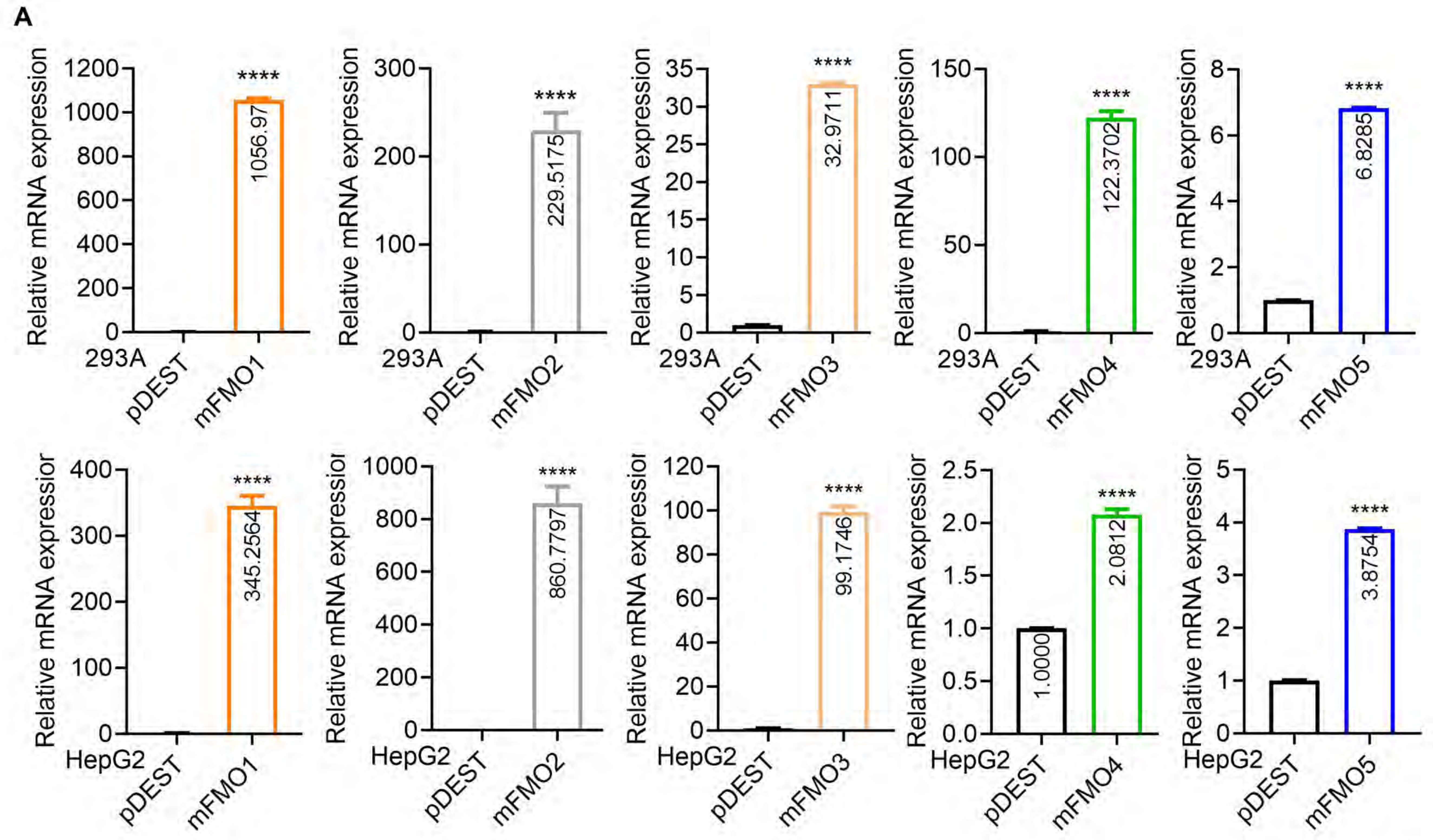
A
Relative mRNA expression
293A
pDEST
mFMO1
mFMO2
mFMO3
mFMO4
mFMO5
****
1056.97
229.5175
32.9711
122.3702
6.8285
HepG2
345.2564
860.7797
99.1746
1.0000
2.0812
3.8754

Figure S2

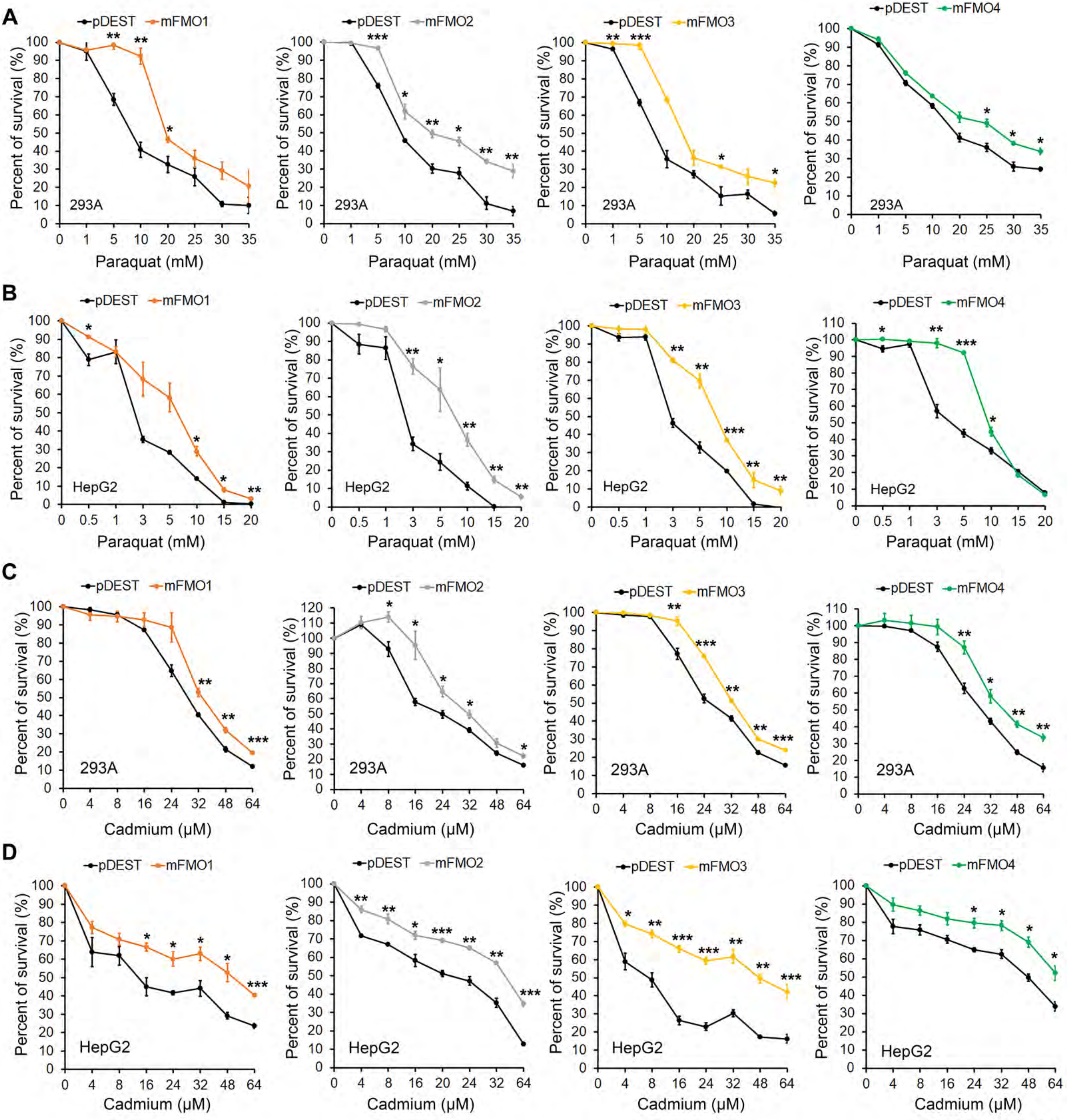
A
pDEST
mFMO1
mFMO2
mFMO3
mFMO4
Percent of survival (%)
Paraquat (mM)
293A
B
pDEST
mFMO1
mFMO2
mFMO3
mFMO4
Percent of survival (%)
Paraquat (mM)
HepG2
C
pDEST
mFMO1
mFMO2
mFMO3
mFMO4
Percent of survival (%)
Cadmium (μM)
293A
D
pDEST
mFMO1
mFMO2
mFMO3
mFMO4
Percent of survival (%)
Cadmium (μM)
HepG2

Figure S3

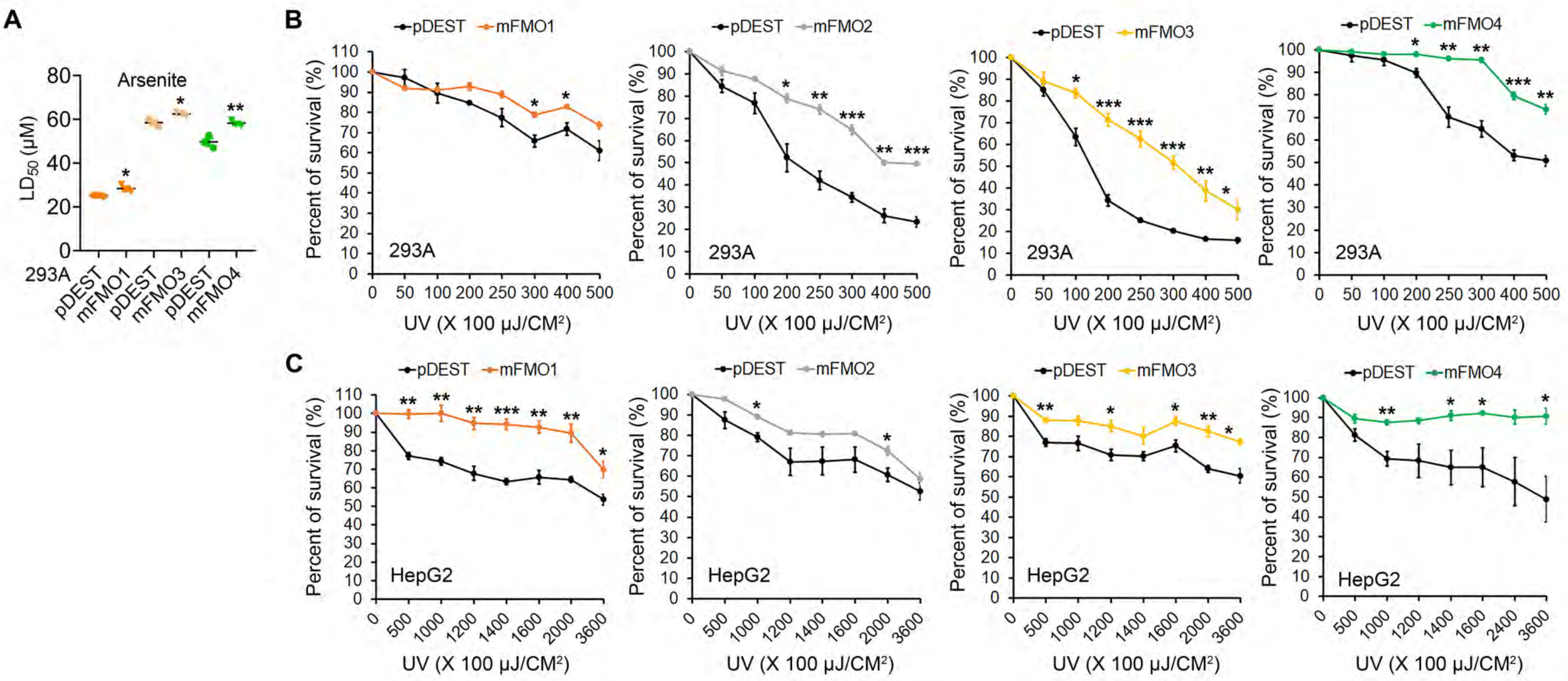
A
Arsenite
LD50 (μM)
293A
pDEST
mFMO1
pDEST
mFMO3
pDEST
mFMO4
B
pDEST
mFMO1
mFMO2
mFMO3
mFMO4
Percent of survival (%)
293A
UV (X 100 μJ/CM²)
C
HepG2

Figure S4

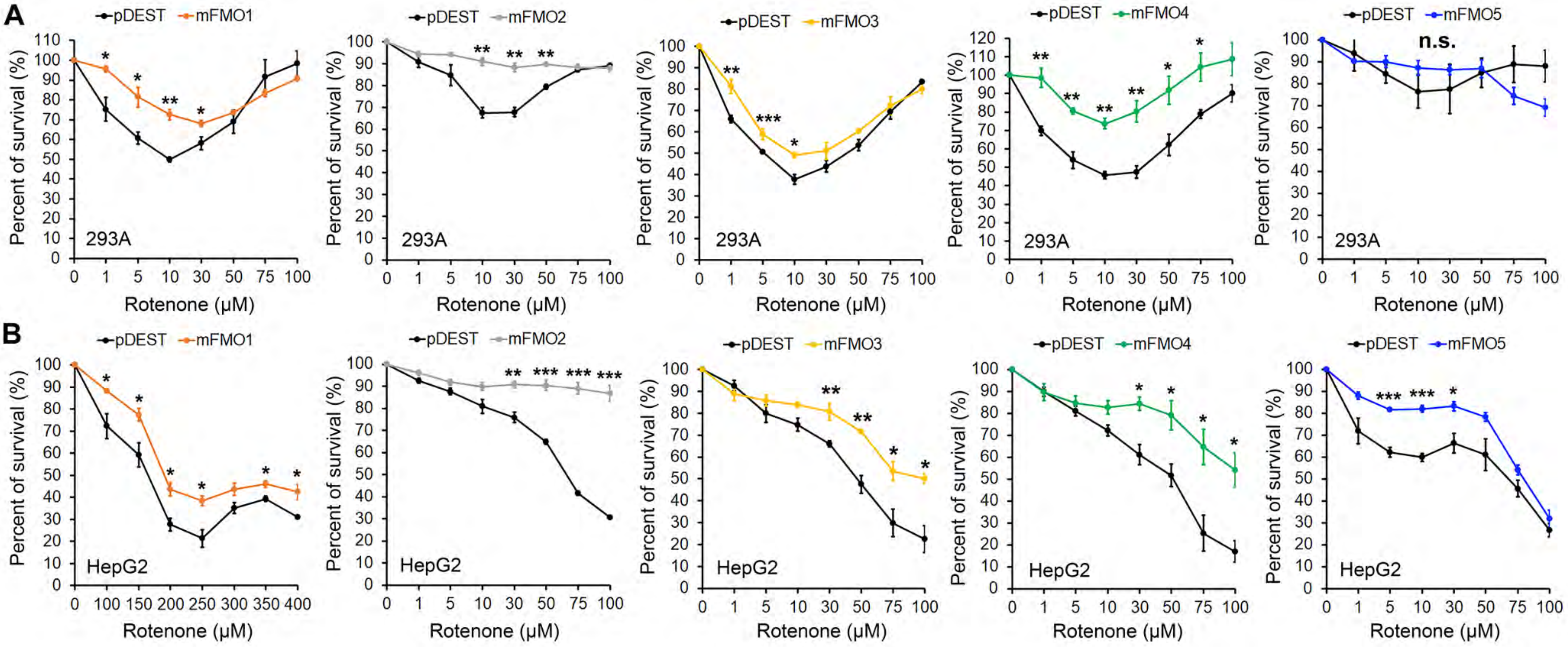
A
B
pDEST
mFMO1
mFMO2
mFMO3
mFMO4
mFMO5
Percent of survival (%)
Rotenone (μM)
293A
HepG2
n.s.
Figure S5

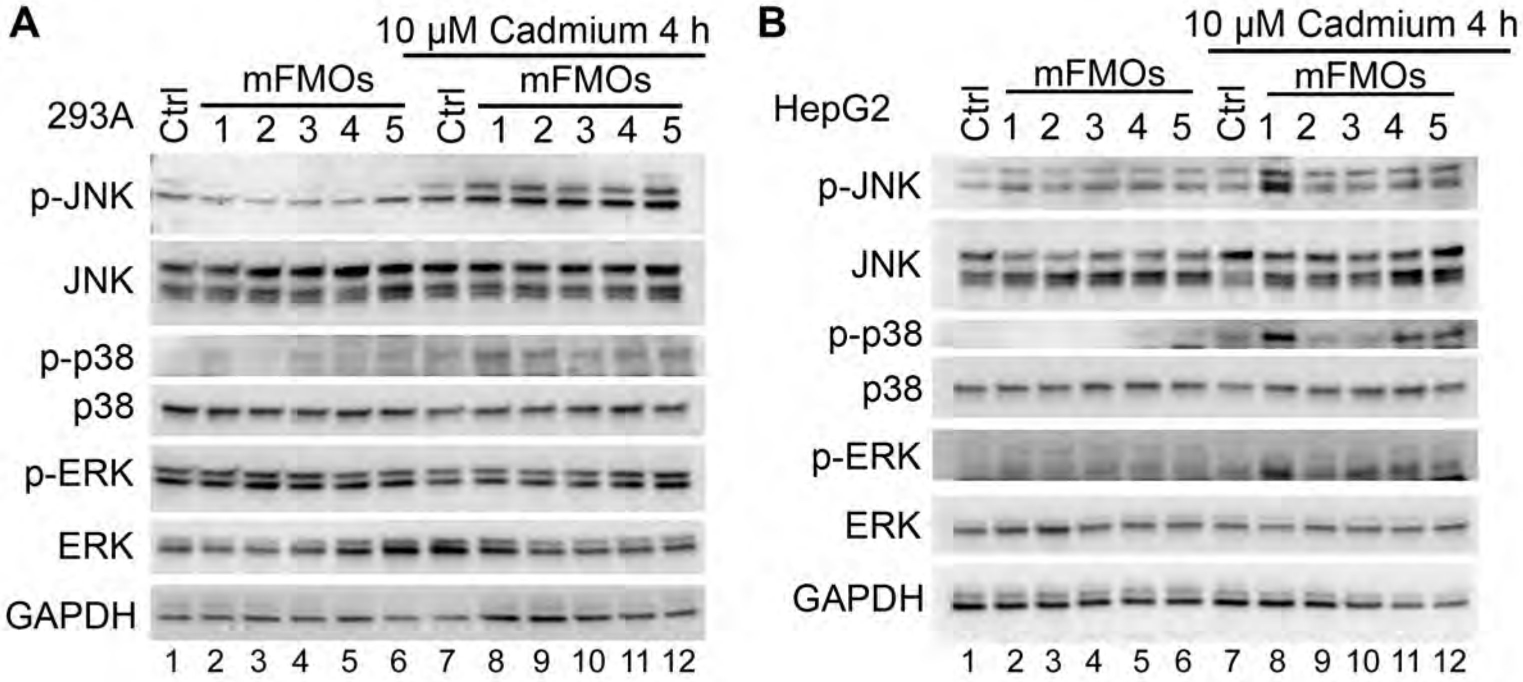
A
10 μM Cadmium 4 h
293A
Ctrl
mFMOs
1 2 3 4 5
Ctrl
mFMOs
1 2 3 4 5
p-JNK
JNK
p-p38
p38
p-ERK
ERK
GAPDH
1 2 3 4 5 6 7 8 9 10 11 12
B
10 μM Cadmium 4 h
HepG2
Ctrl
mFMOs
1 2 3 4 5
Ctrl
mFMOs
1 2 3 4 5
p-JNK
JNK
p-p38
p38
p-ERK
ERK
GAPDH
1 2 3 4 5 6 7 8 9 10 11 12

Figure S6

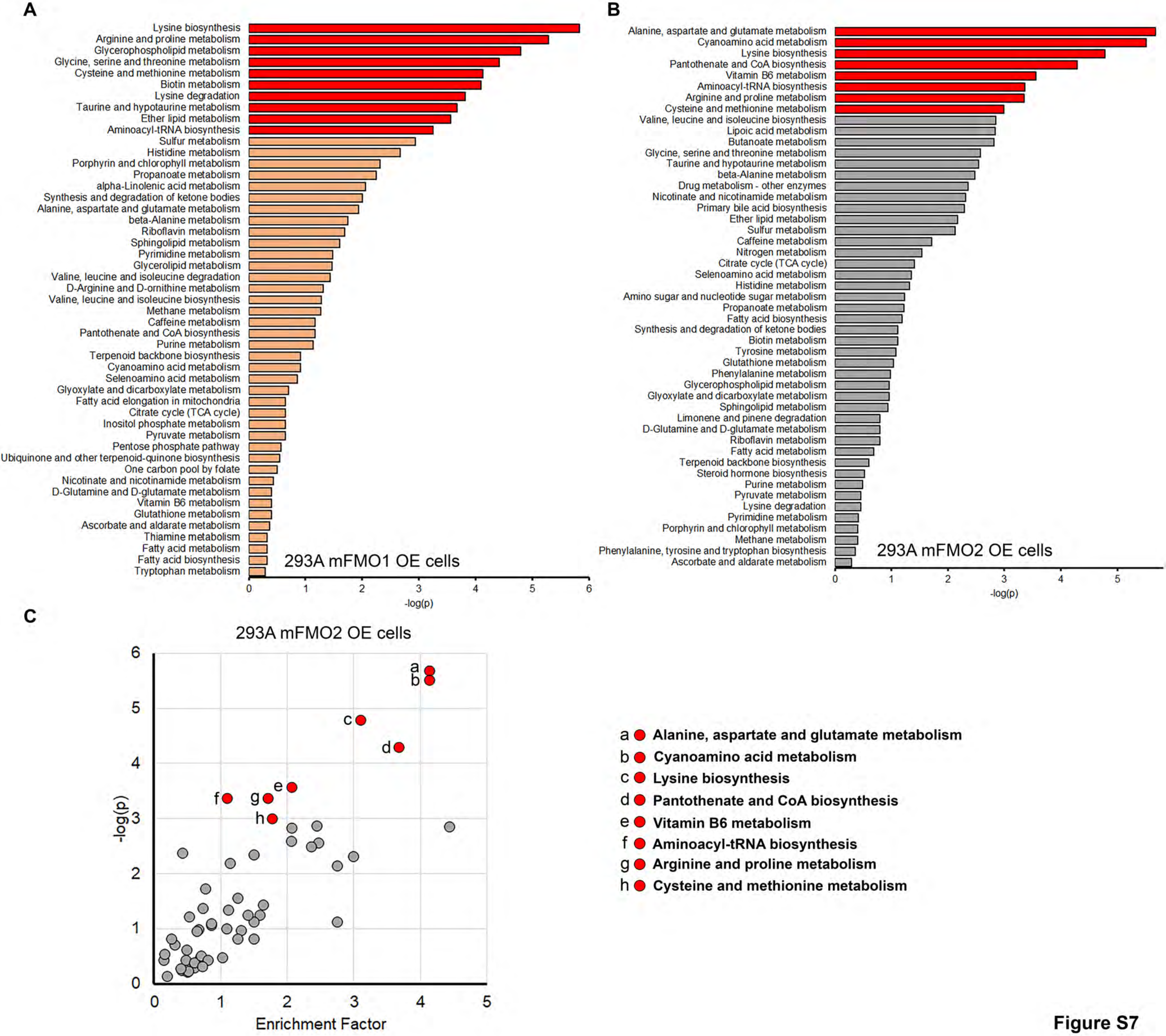

A
Lysine biosynthesis
Arginine and proline metabolism
Glycerophospholipid metabolism
Glycine, serine and threonine metabolism
Cysteine and methionine metabolism
Biotin metabolism
Lysine degradation
Taurine and hypotaurine metabolism
Ether lipid metabolism
Aminoacyl-tRNA biosynthesis
Sulfur metabolism
Histidine metabolism
Porphyrin and chlorophyll metabolism
Propanoate metabolism
alpha-Linolenic acid metabolism
Synthesis and degradation of ketone bodies
Alanine, aspartate and glutamate metabolism
beta-Alanine metabolism
Riboflavin metabolism
Sphingolipid metabolism
Pyrimidine metabolism
Glycerolipid metabolism
Valine, leucine and isoleucine degradation
D-Arginine and D-ornithine metabolism
Valine, leucine and isoleucine biosynthesis
Methane metabolism
Caffeine metabolism
Pantothenate and CoA biosynthesis
Purine metabolism
Terpenoid backbone biosynthesis
Cyanoamino acid metabolism
Selenoamino acid metabolism
Glyoxylate and dicarboxylate metabolism
Fatty acid elongation in mitochondria
Citrate cycle (TCA cycle)
Inositol phosphate metabolism
Pyruvate metabolism
Pentose phosphate pathway
Ubiquinone and other terpenoid-quinone biosynthesis
One carbon pool by folate
Nicotinate and nicotinamide metabolism
D-Glutamine and D-glutamate metabolism
Vitamin B6 metabolism
Glutathione metabolism
Ascorbate and aldarate metabolism
Thiamine metabolism
Fatty acid metabolism
Fatty acid biosynthesis
Tryptophan metabolism
293A mFMO1 OE cells
0 1 2 3 4 5 6
-log(p)
B
Alanine, aspartate and glutamate metabolism
Cyanoamino acid metabolism
Lysine biosynthesis
Pantothenate and CoA biosynthesis
Vitamin B6 metabolism
Aminoacyl-tRNA biosynthesis
Arginine and proline metabolism
Cysteine and methionine metabolism
Valine, leucine and isoleucine biosynthesis
Lipoic acid metabolism
Butanoate metabolism
Glycine, serine and threonine metabolism
Taurine and hypotaurine metabolism
beta-Alanine metabolism
Drug metabolism - other enzymes
Nicotinate and nicotinamide metabolism
Primary bile acid biosynthesis
Ether lipid metabolism
Sulfur metabolism
Caffeine metabolism
Nitrogen metabolism
Citrate cycle (TCA cycle)
Selenoamino acid metabolism
Histidine metabolism
Amino sugar and nucleotide sugar metabolism
Propanoate metabolism
Fatty acid biosynthesis
Synthesis and degradation of ketone bodies
Biotin metabolism
Tyrosine metabolism
Glutathione metabolism
Phenylalanine metabolism
Glycerophospholipid metabolism
Glyoxylate and dicarboxylate metabolism
Sphingolipid metabolism
Limonene and pinene degradation
D-Glutamine and D-glutamate metabolism
Riboflavin metabolism
Fatty acid metabolism
Terpenoid backbone biosynthesis
Steroid hormone biosynthesis
Purine metabolism
Pyruvate metabolism
Lysine degradation
Pyrimidine metabolism
Porphyrin and chlorophyll metabolism
Methane metabolism
Phenylalanine, tyrosine and tryptophan biosynthesis
Ascorbate and aldarate metabolism
293A mFMO2 OE cells
0 1 2 3 4 5
-log(p)
C
293A mFMO2 OE cells
0 1 2 3 4 5 6
-log(p)
0 1 2 3 4 5
Enrichment Factor
a b c d e f g h
a Alanine, aspartate and glutamate metabolism
b Cyanoamino acid metabolism
c Lysine biosynthesis
d Pantothenate and CoA biosynthesis
e Vitamin B6 metabolism
f Aminoacyl-tRNA biosynthesis
g Arginine and proline metabolism
h Cysteine and methionine metabolism

Figure S7

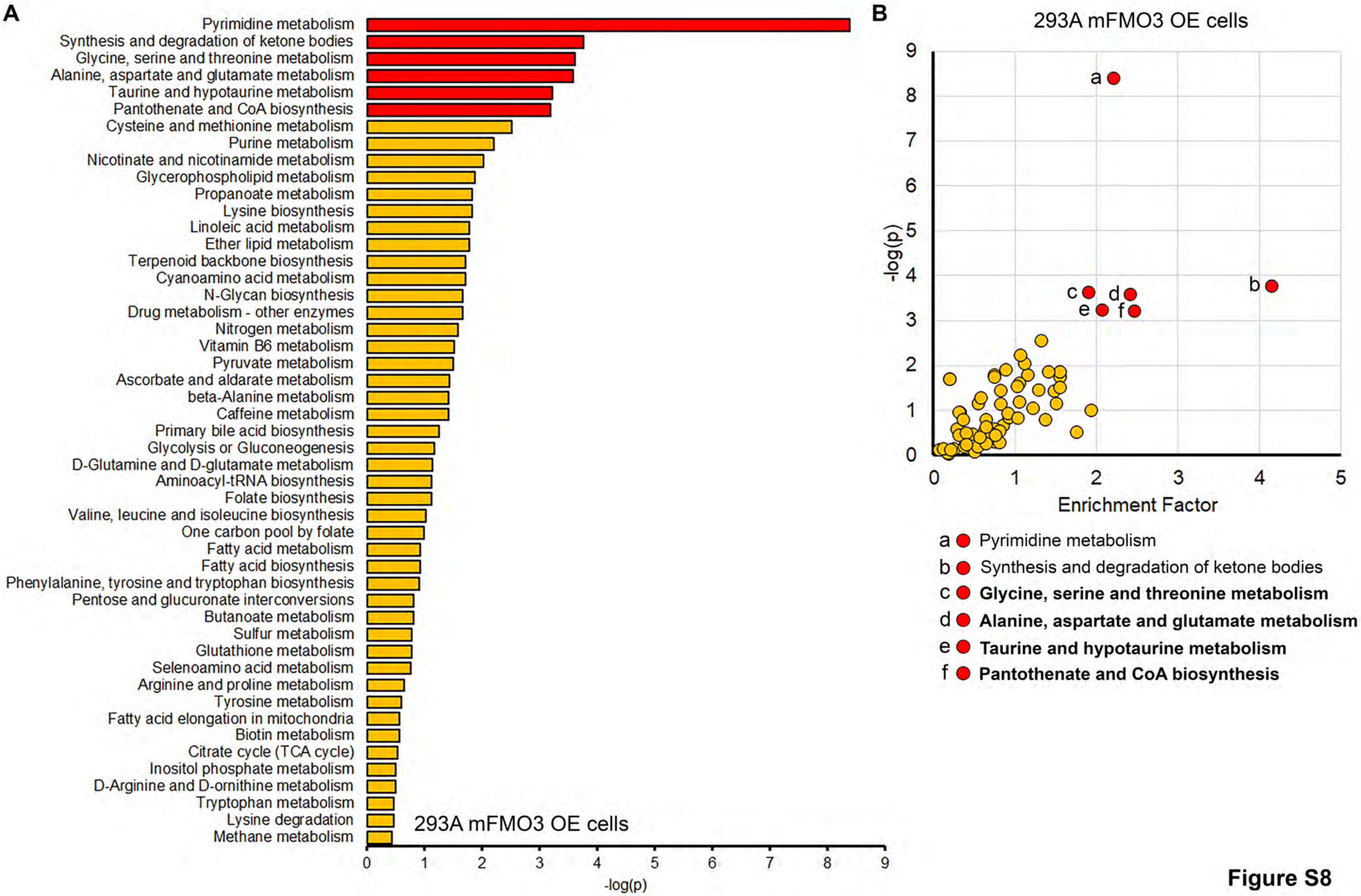
A
Pyrimidine metabolism
Synthesis and degradation of ketone bodies
Glycine, serine and threonine metabolism
Alanine, aspartate and glutamate metabolism
Taurine and hypotaurine metabolism
Pantothenate and CoA biosynthesis
Cysteine and methionine metabolism
Purine metabolism
Nicotinate and nicotinamide metabolism
Glycerophospholipid metabolism
Propanoate metabolism
Lysine biosynthesis
Linoleic acid metabolism
Ether lipid metabolism
Terpenoid backbone biosynthesis
Cyanoamino acid metabolism
N-Glycan biosynthesis
Drug metabolism - other enzymes
Nitrogen metabolism
Vitamin B6 metabolism
Pyruvate metabolism
Ascorbate and aldarate metabolism
beta-Alanine metabolism
Caffeine metabolism
Primary bile acid biosynthesis
Glycolysis or Gluconeogenesis
D-Glutamine and D-glutamate metabolism
Aminoacyl-tRNA biosynthesis
Folate biosynthesis
Valine, leucine and isoleucine biosynthesis
One carbon pool by folate
Fatty acid metabolism
Fatty acid biosynthesis
Phenylalanine, tyrosine and tryptophan biosynthesis
Pentose and glucuronate interconversions
Butanoate metabolism
Sulfur metabolism
Glutathione metabolism
Selenoamino acid metabolism
Arginine and proline metabolism
Tyrosine metabolism
Fatty acid elongation in mitochondria
Biotin metabolism
Citrate cycle (TCA cycle)
Inositol phosphate metabolism
D-Arginine and D-ornithine metabolism
Tryptophan metabolism
Lysine degradation
Methane metabolism
293A mFMO3 OE cells
0 1 2 3 4 5 6 7 8 9
-log(p)
B
293A mFMO3 OE cells
-log(p)
0 1 2 3 4 5 6 7 8 9
a b c d e f
0 1 2 3 4 5
Enrichment Factor
a Pyrimidine metabolism
b Synthesis and degradation of ketone bodies
c Glycine, serine and threonine metabolism
d Alanine, aspartate and glutamate metabolism
e Taurine and hypotaurine metabolism
f Pantothenate and CoA biosynthesis

Figure S8

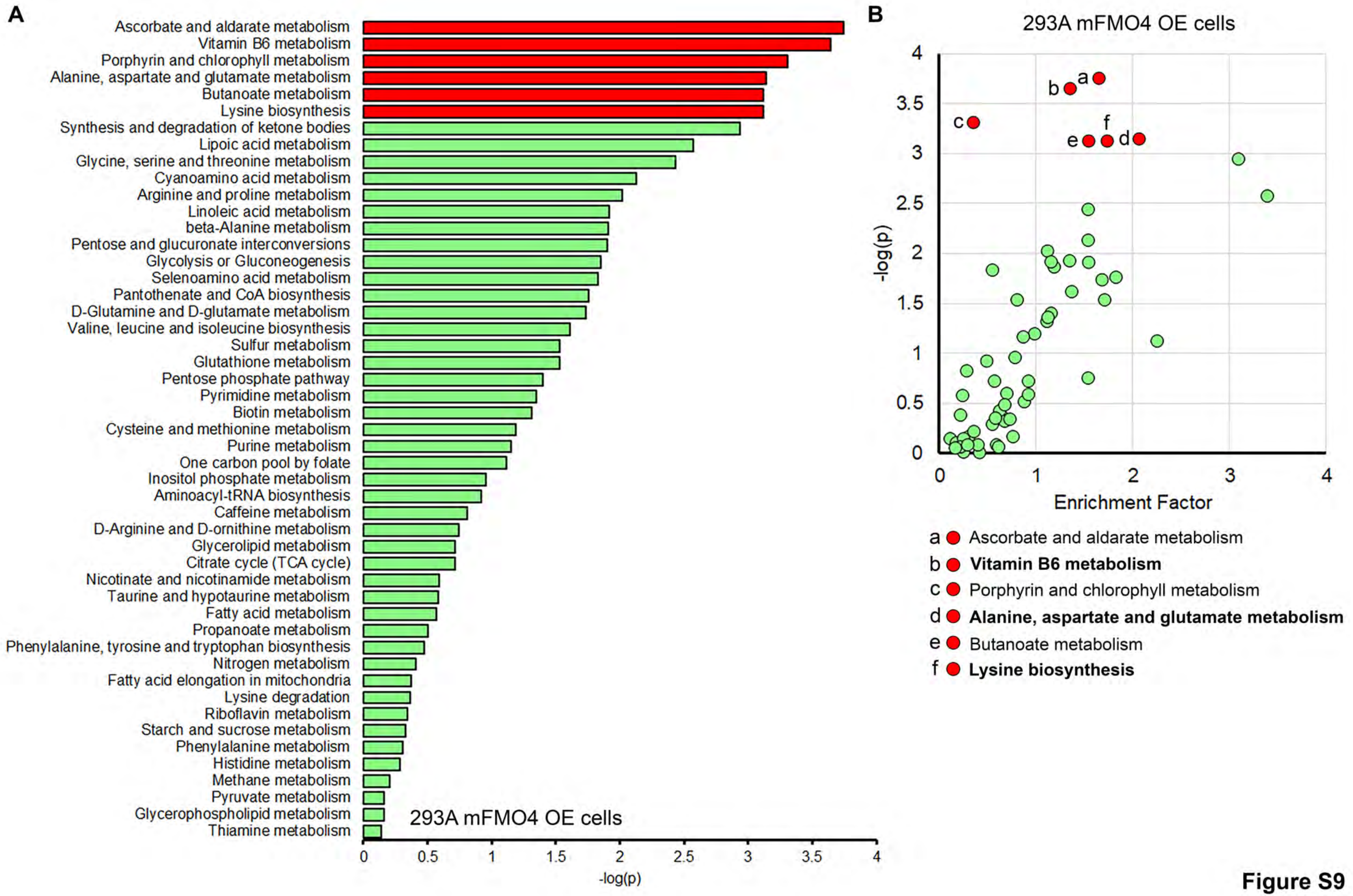
A
Ascorbate and aldarate metabolism
Vitamin B6 metabolism
Porphyrin and chlorophyll metabolism
Alanine, aspartate and glutamate metabolism
Butanoate metabolism
Lysine biosynthesis
Synthesis and degradation of ketone bodies
Lipoic acid metabolism
Glycine, serine and threonine metabolism
Cyanoamino acid metabolism
Arginine and proline metabolism
Linoleic acid metabolism
beta-Alanine metabolism
Pentose and glucuronate interconversions
Glycolysis or Gluconeogenesis
Selenoamino acid metabolism
Pantothenate and CoA biosynthesis
D-Glutamine and D-glutamate metabolism
Valine, leucine and isoleucine biosynthesis
Sulfur metabolism
Glutathione metabolism
Pentose phosphate pathway
Pyrimidine metabolism
Biotin metabolism
Cysteine and methionine metabolism
Purine metabolism
One carbon pool by folate
Inositol phosphate metabolism
Aminoacyl-tRNA biosynthesis
Caffeine metabolism
D-Arginine and D-ornithine metabolism
Glycerolipid metabolism
Citrate cycle (TCA cycle)
Nicotinate and nicotinamide metabolism
Taurine and hypotaurine metabolism
Fatty acid metabolism
Propanoate metabolism
Phenylalanine, tyrosine and tryptophan biosynthesis
Nitrogen metabolism
Fatty acid elongation in mitochondria
Lysine degradation
Riboflavin metabolism
Starch and sucrose metabolism
Phenylalanine metabolism
Histidine metabolism
Methane metabolism
Pyruvate metabolism
Glycerophospholipid metabolism
Thiamine metabolism
293A mFMO4 OE cells
0 0.5 1 1.5 2 2.5 3 3.5 4
-log(p)
B
293A mFMO4 OE cells
-log(p)
Enrichment Factor
a Ascorbate and aldarate metabolism
b Vitamin B6 metabolism
c Porphyrin and chlorophyll metabolism
d Alanine, aspartate and glutamate metabolism
e Butanoate metabolism
f Lysine biosynthesis

Figure S9

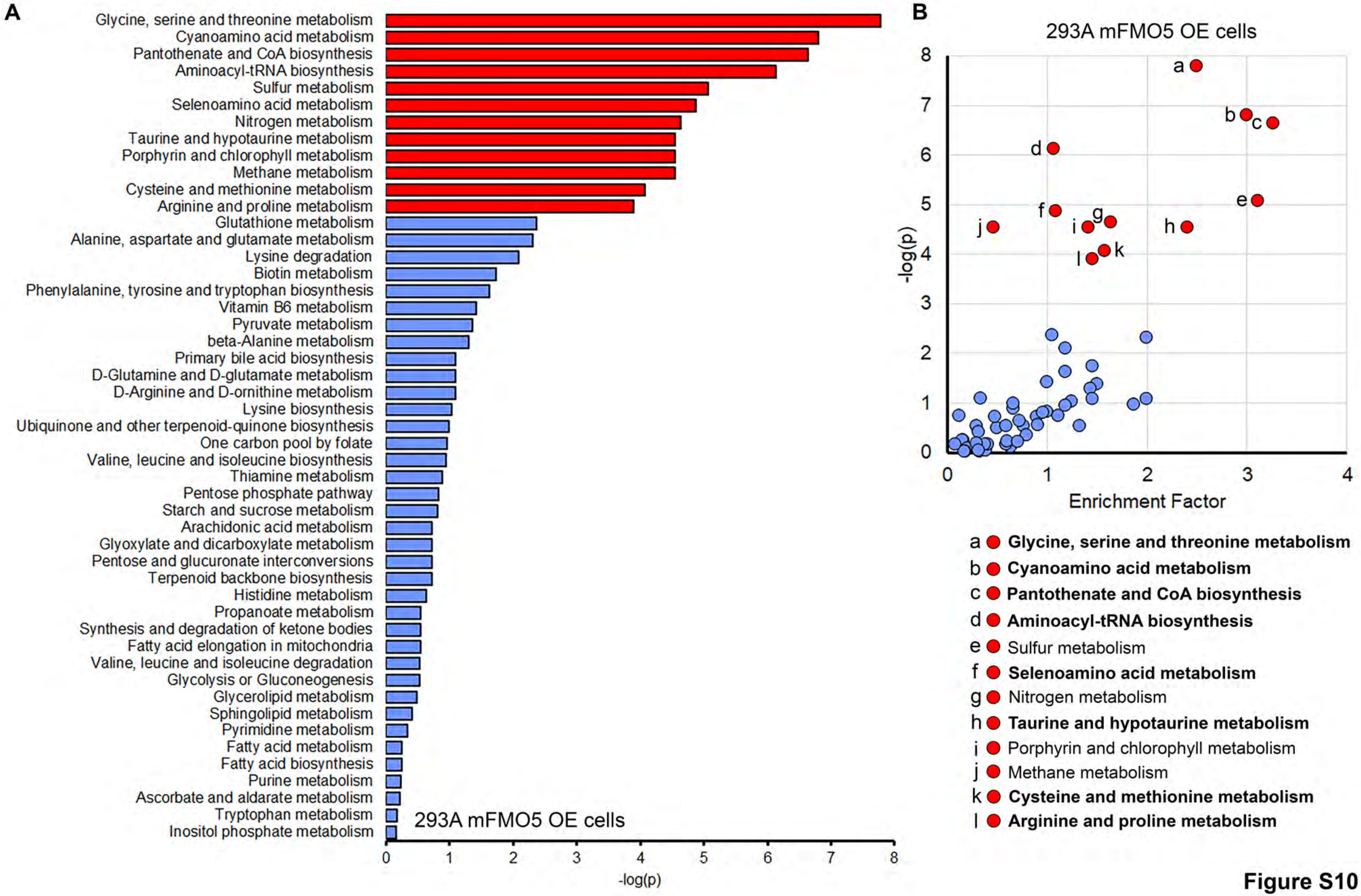
A
Glycine, serine and threonine metabolism
Cyanoamino acid metabolism
Pantothenate and CoA biosynthesis
Aminoacyl-tRNA biosynthesis
Sulfur metabolism
Selenoamino acid metabolism
Nitrogen metabolism
Taurine and hypotaurine metabolism
Porphyrin and chlorophyll metabolism
Methane metabolism
Cysteine and methionine metabolism
Arginine and proline metabolism
Glutathione metabolism
Alanine, aspartate and glutamate metabolism
Lysine degradation
Biotin metabolism
Phenylalanine, tyrosine and tryptophan biosynthesis
Vitamin B6 metabolism
Pyruvate metabolism
beta-Alanine metabolism
Primary bile acid biosynthesis
D-Glutamine and D-glutamate metabolism
D-Arginine and D-ornithine metabolism
Lysine biosynthesis
Ubiquinone and other terpenoid-quinone biosynthesis
One carbon pool by folate
Valine, leucine and isoleucine biosynthesis
Thiamine metabolism
Pentose phosphate pathway
Starch and sucrose metabolism
Arachidonic acid metabolism
Glyoxylate and dicarboxylate metabolism
Pentose and glucuronate interconversions
Terpenoid backbone biosynthesis
Histidine metabolism
Propanoate metabolism
Synthesis and degradation of ketone bodies
Fatty acid elongation in mitochondria
Valine, leucine and isoleucine degradation
Glycolysis or Gluconeogenesis
Glycerolipid metabolism
Sphingolipid metabolism
Pyrimidine metabolism
Fatty acid metabolism
Fatty acid biosynthesis
Purine metabolism
Ascorbate and aldarate metabolism
Tryptophan metabolism
Inositol phosphate metabolism
293A mFMO5 OE cells
0 1 2 3 4 5 6 7 8
-log(p)
B
293A mFMO5 OE cells
-log(p)
Enrichment Factor
0 1 2 3 4
0 1 2 3 4 5 6 7 8
a b c d e f g h i j k l
a Glycine, serine and threonine metabolism
b Cyanoamino acid metabolism
c Pantothenate and CoA biosynthesis
d Aminoacyl-tRNA biosynthesis
e Sulfur metabolism
f Selenoamino acid metabolism
g Nitrogen metabolism
h Taurine and hypotaurine metabolism
i Porphyrin and chlorophyll metabolism
j Methane metabolism
k Cysteine and methionine metabolism
l Arginine and proline metabolism

Figure S10

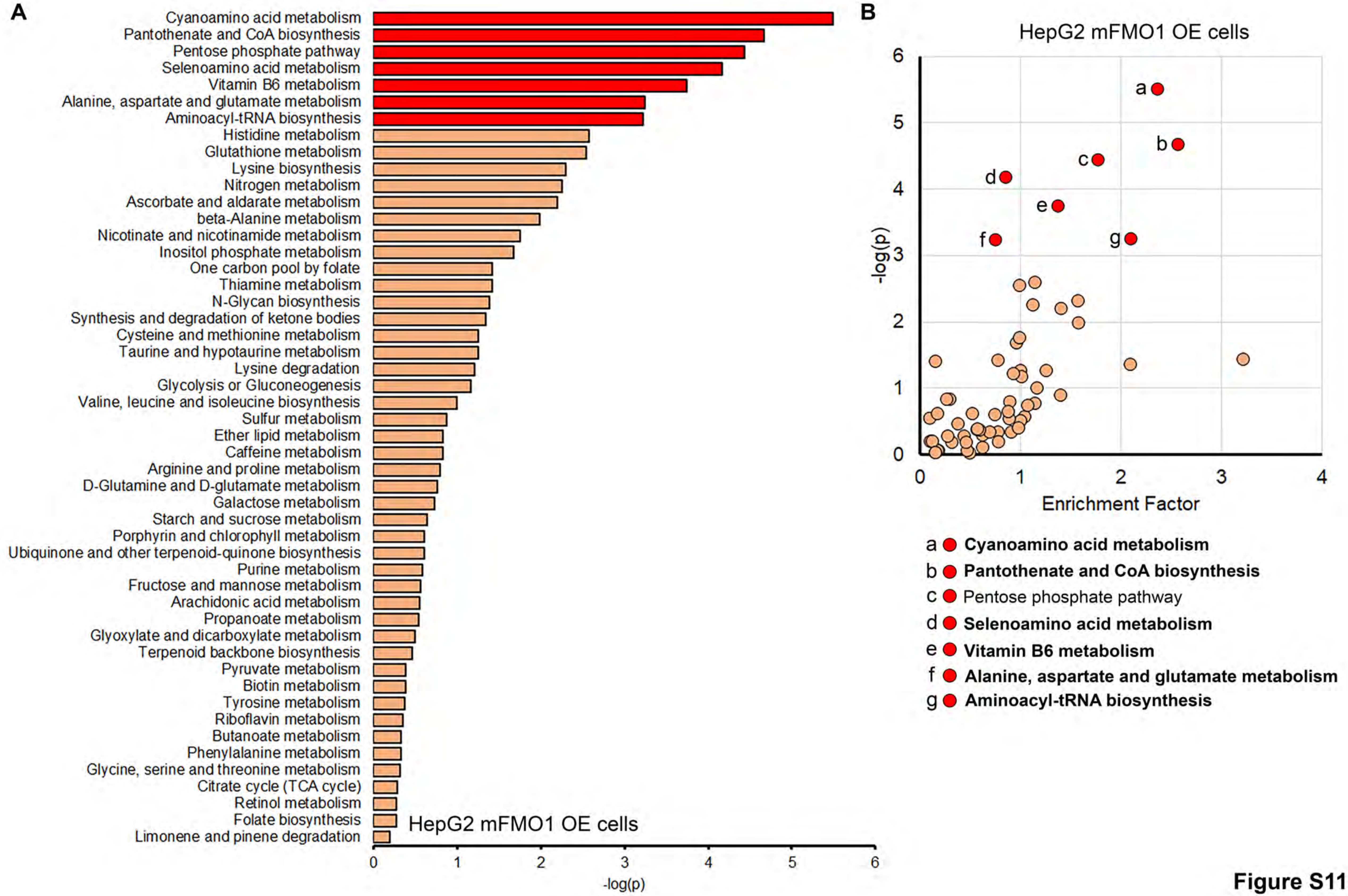
A
Cyanoamino acid metabolism
Pantothenate and CoA biosynthesis
Pentose phosphate pathway
Selenoamino acid metabolism
Vitamin B6 metabolism
Alanine, aspartate and glutamate metabolism
Aminoacyl-tRNA biosynthesis
Histidine metabolism
Glutathione metabolism
Lysine biosynthesis
Nitrogen metabolism
Ascorbate and aldarate metabolism
beta-Alanine metabolism
Nicotinate and nicotinamide metabolism
Inositol phosphate metabolism
One carbon pool by folate
Thiamine metabolism
N-Glycan biosynthesis
Synthesis and degradation of ketone bodies
Cysteine and methionine metabolism
Taurine and hypotaurine metabolism
Lysine degradation
Glycolysis or Gluconeogenesis
Valine, leucine and isoleucine biosynthesis
Sulfur metabolism
Ether lipid metabolism
Caffeine metabolism
Arginine and proline metabolism
D-Glutamine and D-glutamate metabolism
Galactose metabolism
Starch and sucrose metabolism
Porphyrin and chlorophyll metabolism
Ubiquinone and other terpenoid-quinone biosynthesis
Purine metabolism
Fructose and mannose metabolism
Arachidonic acid metabolism
Propanoate metabolism
Glyoxylate and dicarboxylate metabolism
Terpenoid backbone biosynthesis
Pyruvate metabolism
Biotin metabolism
Tyrosine metabolism
Riboflavin metabolism
Butanoate metabolism
Phenylalanine metabolism
Glycine, serine and threonine metabolism
Citrate cycle (TCA cycle)
Retinol metabolism
Folate biosynthesis
Limonene and pinene degradation
HepG2 mFMO1 OE cells
0 1 2 3 4 5 6
-log(p)
B
HepG2 mFMO1 OE cells
-log(p)
Enrichment Factor
a b c d e f g
a Cyanoamino acid metabolism
b Pantothenate and CoA biosynthesis
c Pentose phosphate pathway
d Selenoamino acid metabolism
e Vitamin B6 metabolism
f Alanine, aspartate and glutamate metabolism
g Aminoacyl-tRNA biosynthesis

Figure S11

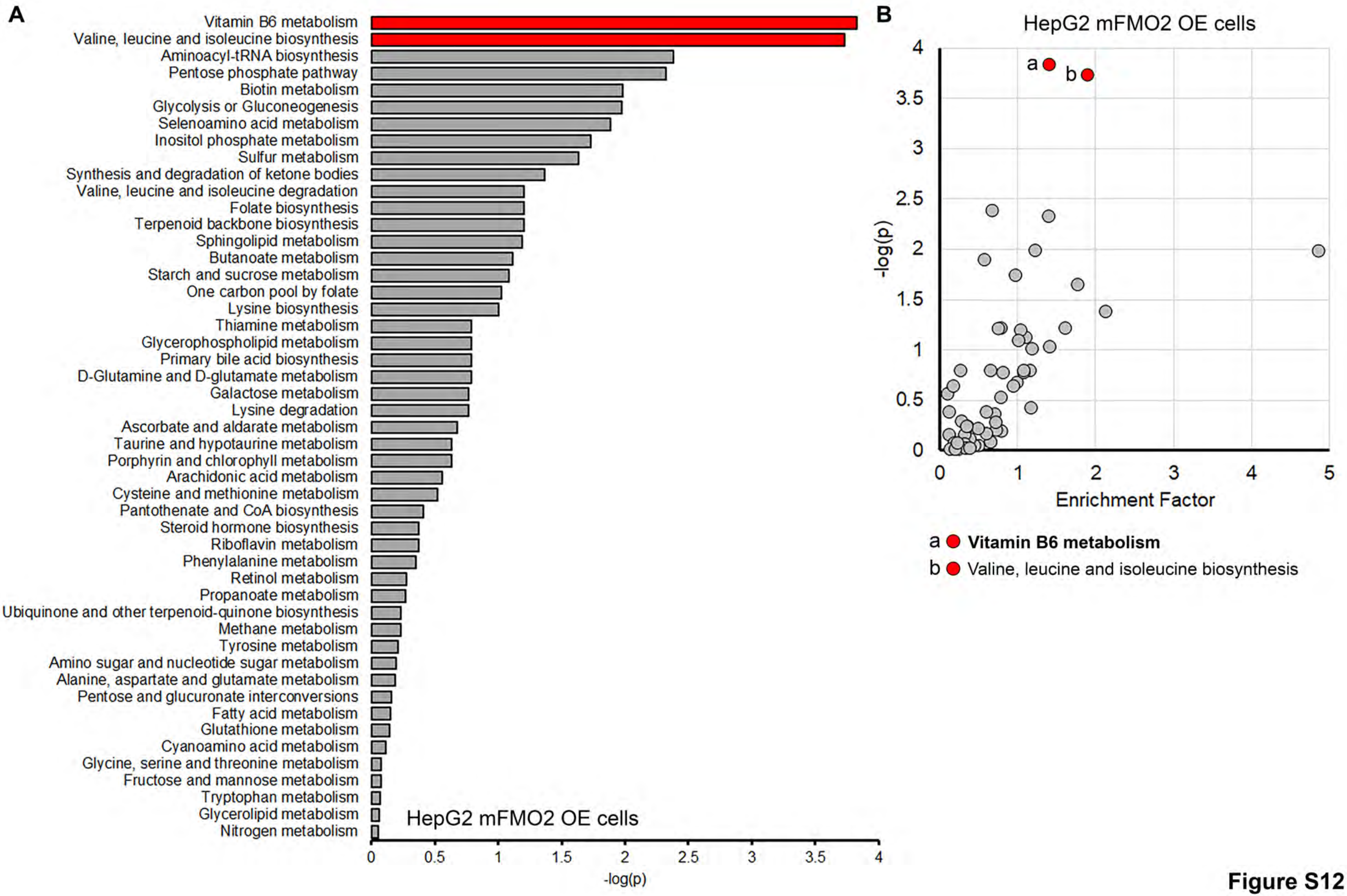
A
Vitamin B6 metabolism
Valine, leucine and isoleucine biosynthesis
Aminoacyl-tRNA biosynthesis
Pentose phosphate pathway
Biotin metabolism
Glycolysis or Gluconeogenesis
Selenoamino acid metabolism
Inositol phosphate metabolism
Sulfur metabolism
Synthesis and degradation of ketone bodies
Valine, leucine and isoleucine degradation
Folate biosynthesis
Terpenoid backbone biosynthesis
Sphingolipid metabolism
Butanoate metabolism
Starch and sucrose metabolism
One carbon pool by folate
Lysine biosynthesis
Thiamine metabolism
Glycerophospholipid metabolism
Primary bile acid biosynthesis
D-Glutamine and D-glutamate metabolism
Galactose metabolism
Lysine degradation
Ascorbate and aldarate metabolism
Taurine and hypotaurine metabolism
Porphyrin and chlorophyll metabolism
Arachidonic acid metabolism
Cysteine and methionine metabolism
Pantothenate and CoA biosynthesis
Steroid hormone biosynthesis
Riboflavin metabolism
Phenylalanine metabolism
Retinol metabolism
Propanoate metabolism
Ubiquinone and other terpenoid-quinone biosynthesis
Methane metabolism
Tyrosine metabolism
Amino sugar and nucleotide sugar metabolism
Alanine, aspartate and glutamate metabolism
Pentose and glucuronate interconversions
Fatty acid metabolism
Glutathione metabolism
Cyanoamino acid metabolism
Glycine, serine and threonine metabolism
Fructose and mannose metabolism
Tryptophan metabolism
Glycerolipid metabolism
Nitrogen metabolism
HepG2 mFMO2 OE cells
0 0.5 1 1.5 2 2.5 3 3.5 4
-log(p)
B
HepG2 mFMO2 OE cells
-log(p)
0 0.5 1 1.5 2 2.5 3 3.5 4
0 1 2 3 4 5
Enrichment Factor
a
b
a Vitamin B6 metabolism
b Valine, leucine and isoleucine biosynthesis

Figure S12

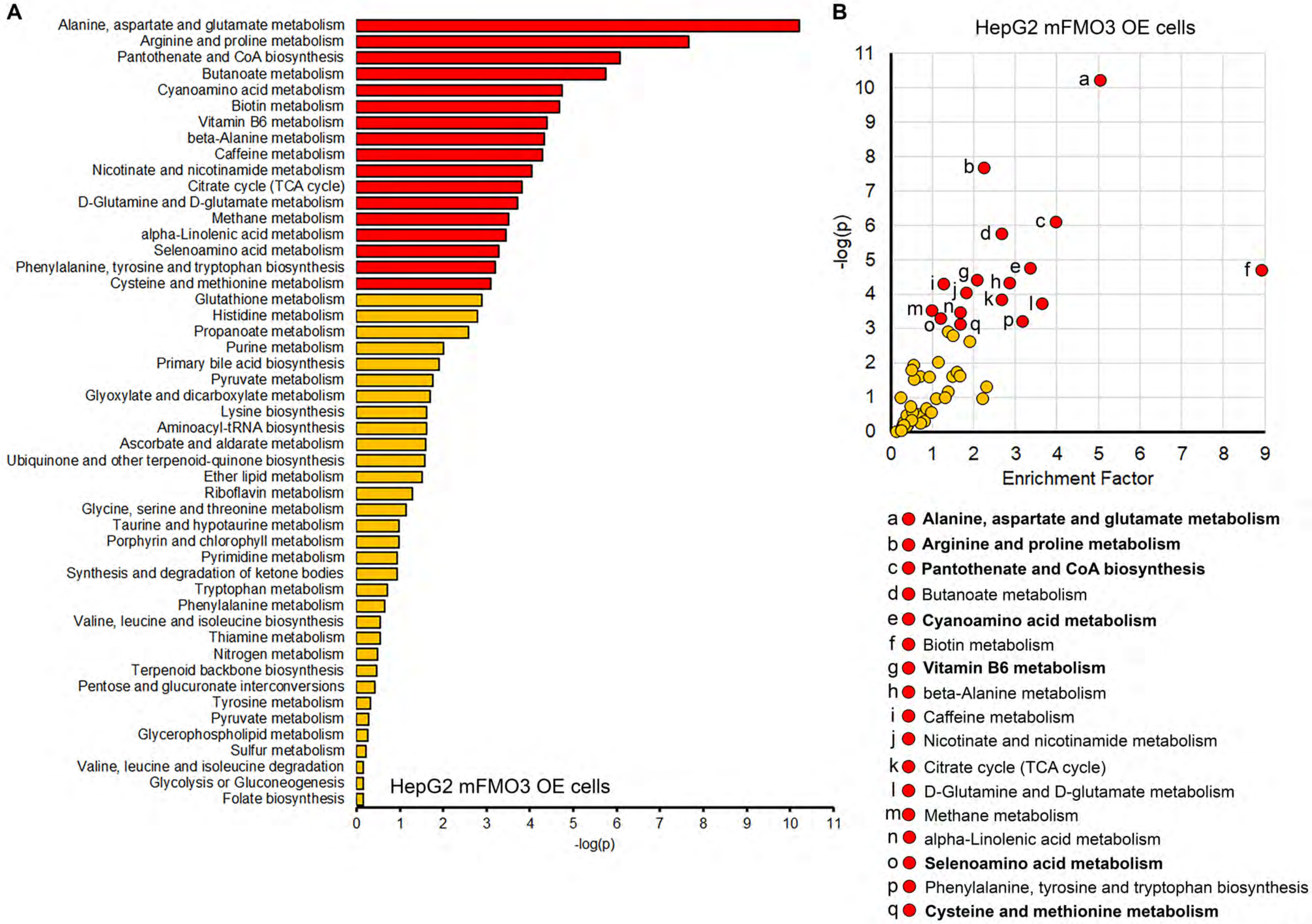
A
Alanine, aspartate and glutamate metabolism
Arginine and proline metabolism
Pantothenate and CoA biosynthesis
Butanoate metabolism
Cyanoamino acid metabolism
Biotin metabolism
Vitamin B6 metabolism
beta-Alanine metabolism
Caffeine metabolism
Nicotinate and nicotinamide metabolism
Citrate cycle (TCA cycle)
D-Glutamine and D-glutamate metabolism
Methane metabolism
alpha-Linolenic acid metabolism
Selenoamino acid metabolism
Phenylalanine, tyrosine and tryptophan biosynthesis
Cysteine and methionine metabolism
Glutathione metabolism
Histidine metabolism
Propanoate metabolism
Purine metabolism
Primary bile acid biosynthesis
Pyruvate metabolism
Glyoxylate and dicarboxylate metabolism
Lysine biosynthesis
Aminoacyl-tRNA biosynthesis
Ascorbate and aldarate metabolism
Ubiquinone and other terpenoid-quinone biosynthesis
Ether lipid metabolism
Riboflavin metabolism
Glycine, serine and threonine metabolism
Taurine and hypotaurine metabolism
Porphyrin and chlorophyll metabolism
Pyrimidine metabolism
Synthesis and degradation of ketone bodies
Tryptophan metabolism
Phenylalanine metabolism
Valine, leucine and isoleucine biosynthesis
Thiamine metabolism
Nitrogen metabolism
Terpenoid backbone biosynthesis
Pentose and glucuronate interconversions
Tyrosine metabolism
Pyruvate metabolism
Glycerophospholipid metabolism
Sulfur metabolism
Valine, leucine and isoleucine degradation
Glycolysis or Gluconeogenesis
Folate biosynthesis
HepG2 mFMO3 OE cells
0 1 2 3 4 5 6 7 8 9 10 11
-log(p)
B
HepG2 mFMO3 OE cells
-log(p)
0 1 2 3 4 5 6 7 8 9 10 11
0 1 2 3 4 5 6 7 8 9
Enrichment Factor
a b c d e f g h i j k l m n o p q
a Alanine, aspartate and glutamate metabolism
b Arginine and proline metabolism
c Pantothenate and CoA biosynthesis
d Butanoate metabolism
e Cyanoamino acid metabolism
f Biotin metabolism
g Vitamin B6 metabolism
h beta-Alanine metabolism
i Caffeine metabolism
j Nicotinate and nicotinamide metabolism
k Citrate cycle (TCA cycle)
l D-Glutamine and D-glutamate metabolism
m Methane metabolism
n alpha-Linolenic acid metabolism
o Selenoamino acid metabolism
p Phenylalanine, tyrosine and tryptophan biosynthesis
q Cysteine and methionine metabolism

Figure S13

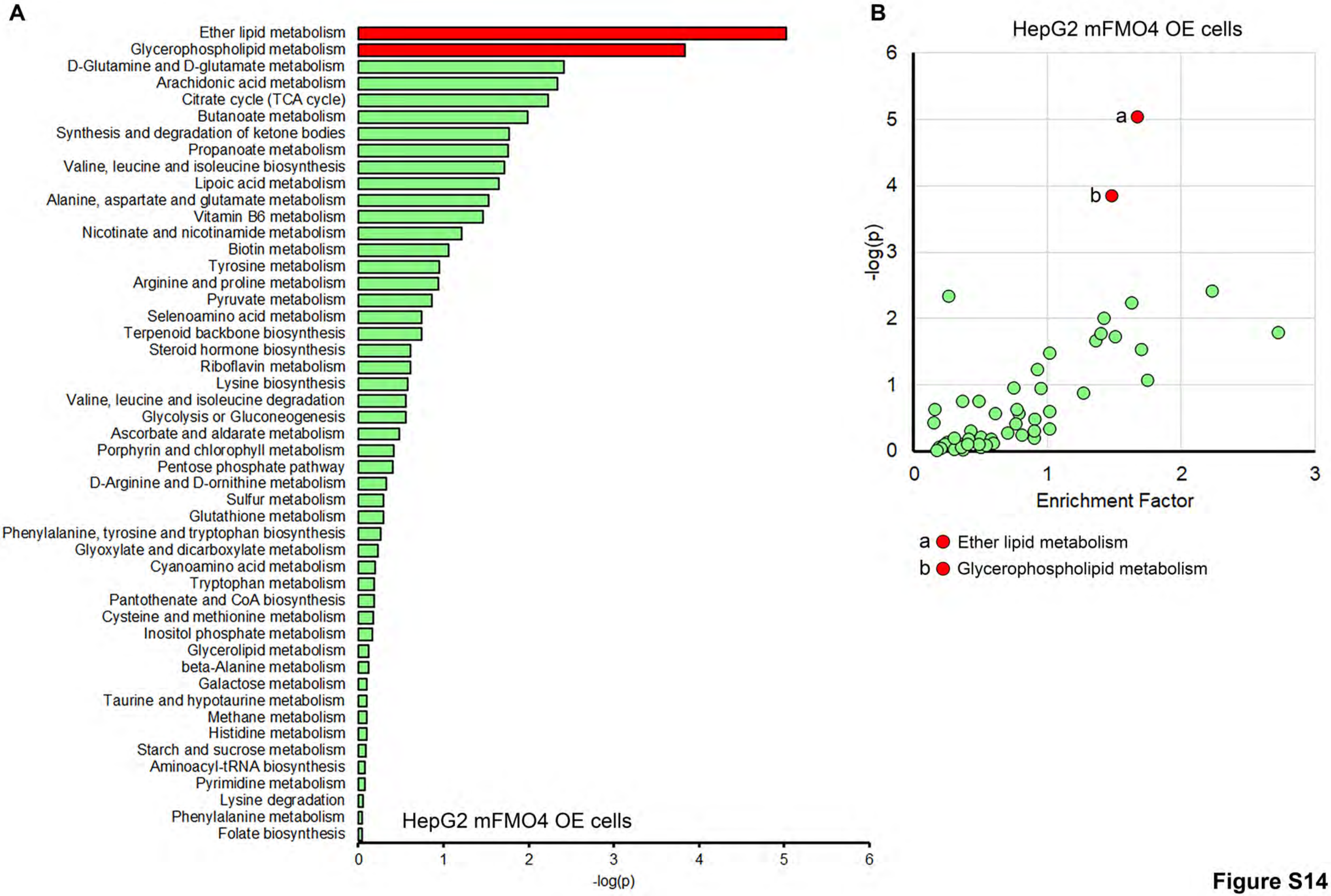
A
Ether lipid metabolism
Glycerophospholipid metabolism
D-Glutamine and D-glutamate metabolism
Arachidonic acid metabolism
Citrate cycle (TCA cycle)
Butanoate metabolism
Synthesis and degradation of ketone bodies
Propanoate metabolism
Valine, leucine and isoleucine biosynthesis
Lipoic acid metabolism
Alanine, aspartate and glutamate metabolism
Vitamin B6 metabolism
Nicotinate and nicotinamide metabolism
Biotin metabolism
Tyrosine metabolism
Arginine and proline metabolism
Pyruvate metabolism
Selenoamino acid metabolism
Terpenoid backbone biosynthesis
Steroid hormone biosynthesis
Riboflavin metabolism
Lysine biosynthesis
Valine, leucine and isoleucine degradation
Glycolysis or Gluconeogenesis
Ascorbate and aldarate metabolism
Porphyrin and chlorophyll metabolism
Pentose phosphate pathway
D-Arginine and D-ornithine metabolism
Sulfur metabolism
Glutathione metabolism
Phenylalanine, tyrosine and tryptophan biosynthesis
Glyoxylate and dicarboxylate metabolism
Cyanoamino acid metabolism
Tryptophan metabolism
Pantothenate and CoA biosynthesis
Cysteine and methionine metabolism
Inositol phosphate metabolism
Glycerolipid metabolism
beta-Alanine metabolism
Galactose metabolism
Taurine and hypotaurine metabolism
Methane metabolism
Histidine metabolism
Starch and sucrose metabolism
Aminoacyl-tRNA biosynthesis
Pyrimidine metabolism
Lysine degradation
Phenylalanine metabolism
Folate biosynthesis
HepG2 mFMO4 OE cells
0 1 2 3 4 5 6
-log(p)
B
HepG2 mFMO4 OE cells
-log(p)
6 5 4 3 2 1 0
a
b
0 1 2 3
Enrichment Factor
a Ether lipid metabolism
b Glycerophospholipid metabolism

Figure S14

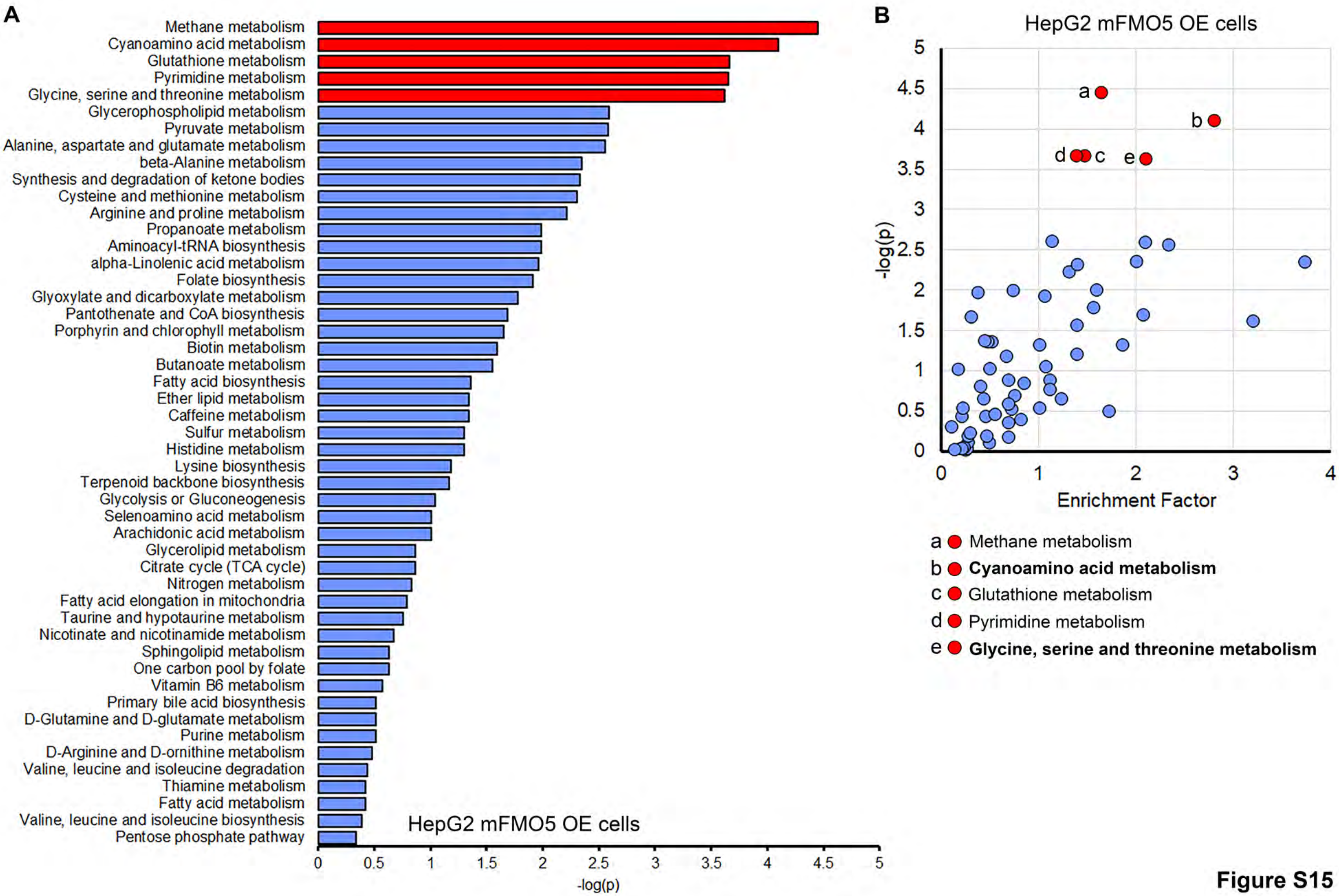
A
Methane metabolism
Cyanoamino acid metabolism
Glutathione metabolism
Pyrimidine metabolism
Glycine, serine and threonine metabolism
Glycerophospholipid metabolism
Pyruvate metabolism
Alanine, aspartate and glutamate metabolism
beta-Alanine metabolism
Synthesis and degradation of ketone bodies
Cysteine and methionine metabolism
Arginine and proline metabolism
Propanoate metabolism
Aminoacyl-tRNA biosynthesis
alpha-Linolenic acid metabolism
Folate biosynthesis
Glyoxylate and dicarboxylate metabolism
Pantothenate and CoA biosynthesis
Porphyrin and chlorophyll metabolism
Biotin metabolism
Butanoate metabolism
Fatty acid biosynthesis
Ether lipid metabolism
Caffeine metabolism
Sulfur metabolism
Histidine metabolism
Lysine biosynthesis
Terpenoid backbone biosynthesis
Glycolysis or Gluconeogenesis
Selenoamino acid metabolism
Arachidonic acid metabolism
Glycerolipid metabolism
Citrate cycle (TCA cycle)
Nitrogen metabolism
Fatty acid elongation in mitochondria
Taurine and hypotaurine metabolism
Nicotinate and nicotinamide metabolism
Sphingolipid metabolism
One carbon pool by folate
Vitamin B6 metabolism
Primary bile acid biosynthesis
D-Glutamine and D-glutamate metabolism
Purine metabolism
D-Arginine and D-ornithine metabolism
Valine, leucine and isoleucine degradation
Thiamine metabolism
Fatty acid metabolism
Valine, leucine and isoleucine biosynthesis
Pentose phosphate pathway
HepG2 mFMO5 OE cells
0 0.5 1 1.5 2 2.5 3 3.5 4 4.5 5
-log(p)
B
HepG2 mFMO5 OE cells
5 4.5 4 3.5 3 2.5 2 1.5 1 0.5 0
-log(p)
a b c d e
0 1 2 3 4
Enrichment Factor
a Methane metabolism
b Cyanoamino acid metabolism
c Glutathione metabolism
d Pyrimidine metabolism
e Glycine, serine and threonine metabolism

Figure S15

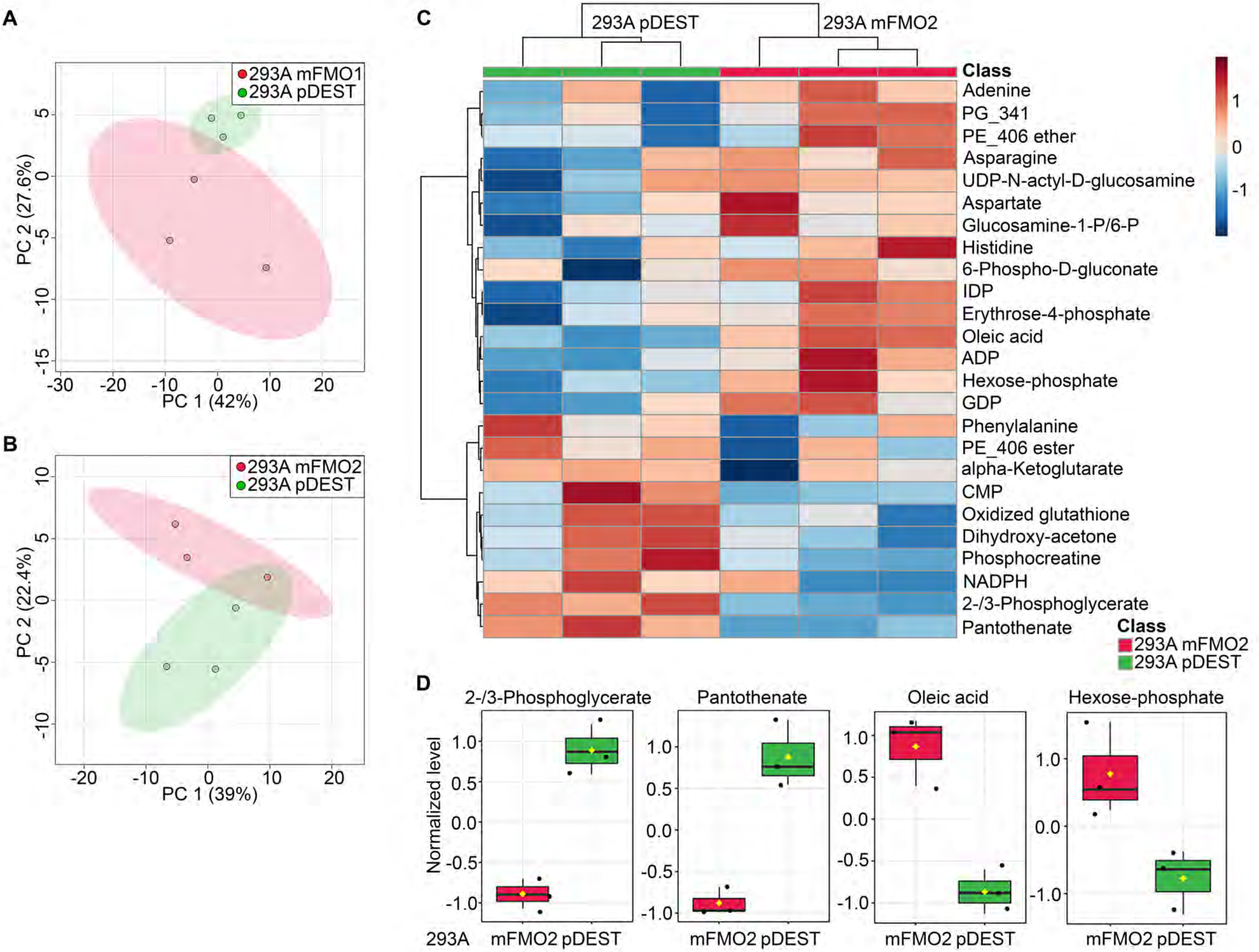
A
293A mFMO1
293A pDEST
PC 2 (27.6%)
PC 1 (42%)
B
293A mFMO2
293A pDEST
PC 2 (22.4%)
PC 1 (39%)
C
293A pDEST
293A mFMO2
Class
Adenine
PG_341
PE_406 ether
Asparagine
UDP-N-actyl-D-glucosamine
Aspartate
Glucosamine-1-P/6-P
Histidine
6-Phospho-D-gluconate
IDP
Erythrose-4-phosphate
Oleic acid
ADP
Hexose-phosphate
GDP
Phenylalanine
PE_406 ester
alpha-Ketoglutarate
CMP
Oxidized glutathione
Dihydroxy-acetone
Phosphocreatine
NADPH
2-/3-Phosphoglycerate
Pantothenate
Class
293A mFMO2
293A pDEST
D
2-/3-Phosphoglycerate
Pantothenate
Oleic acid
Hexose-phosphate
Normalized level
293A
mFMO2 pDEST
mFMO2 pDEST
mFMO2 pDEST
mFMO2 pDEST

Figure S16

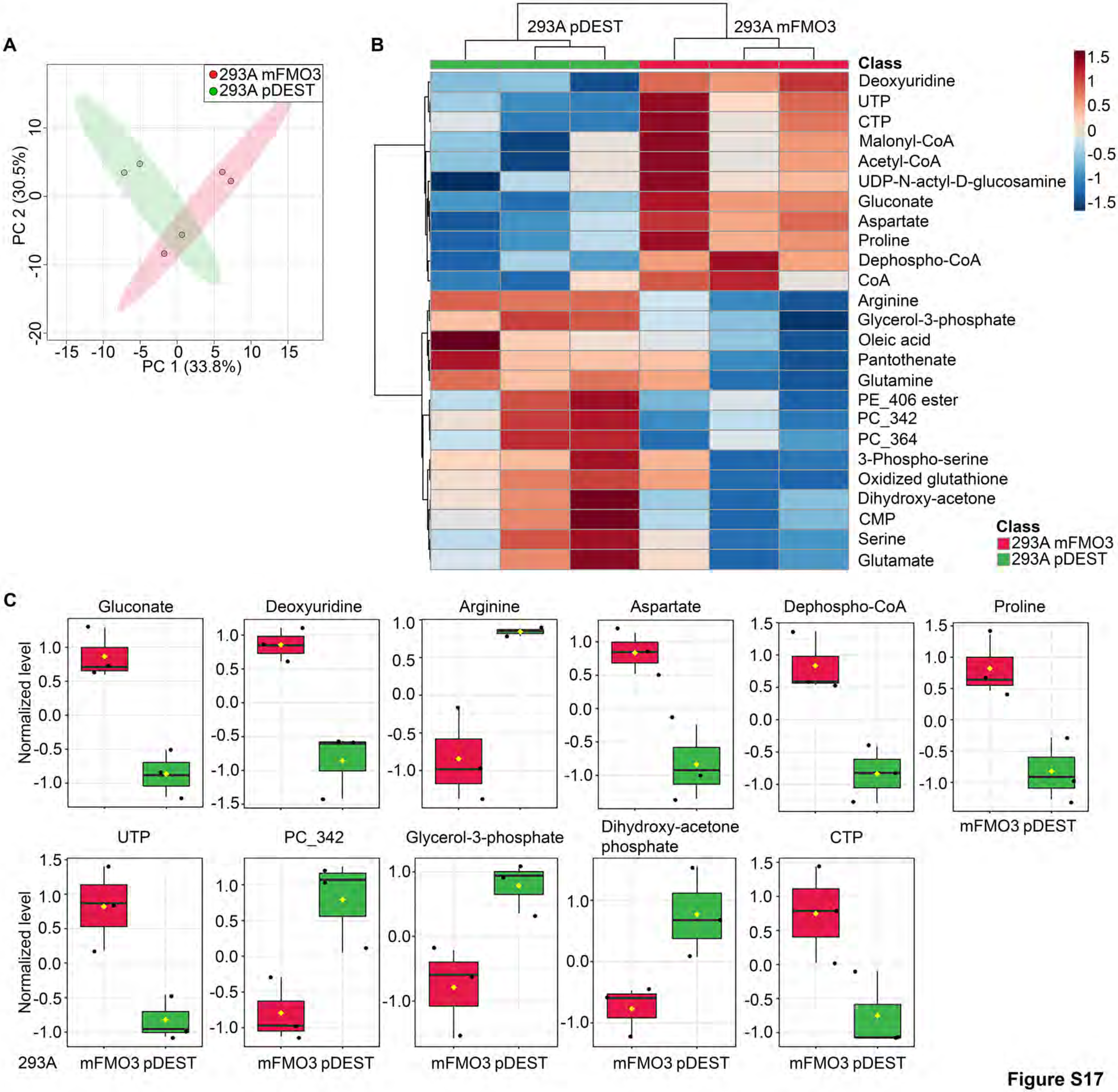
A
293A mFMO3
293A pDEST
PC 2 (30.5%)
PC 1 (33.8%)
B
293A pDEST
293A mFMO3
Class
Deoxyuridine
UTP
CTP
Malonyl-CoA
Acetyl-CoA
UDP-N-actyl-D-glucosamine
Gluconate
Aspartate
Proline
Dephospho-CoA
CoA
Arginine
Glycerol-3-phosphate
Oleic acid
Pantothenate
Glutamine
PE_406 ester
PC_342
PC_364
3-Phospho-serine
Oxidized glutathione
Dihydroxy-acetone
CMP
Serine
Glutamate
Class
293A mFMO3
293A pDEST
C
Gluconate
Deoxyuridine
Arginine
Aspartate
Dephospho-CoA
Proline
Normalized level
UTP
PC_342
Glycerol-3-phosphate
Dihydroxy-acetone phosphate
CTP
293A
mFMO3 pDEST

Figure S17

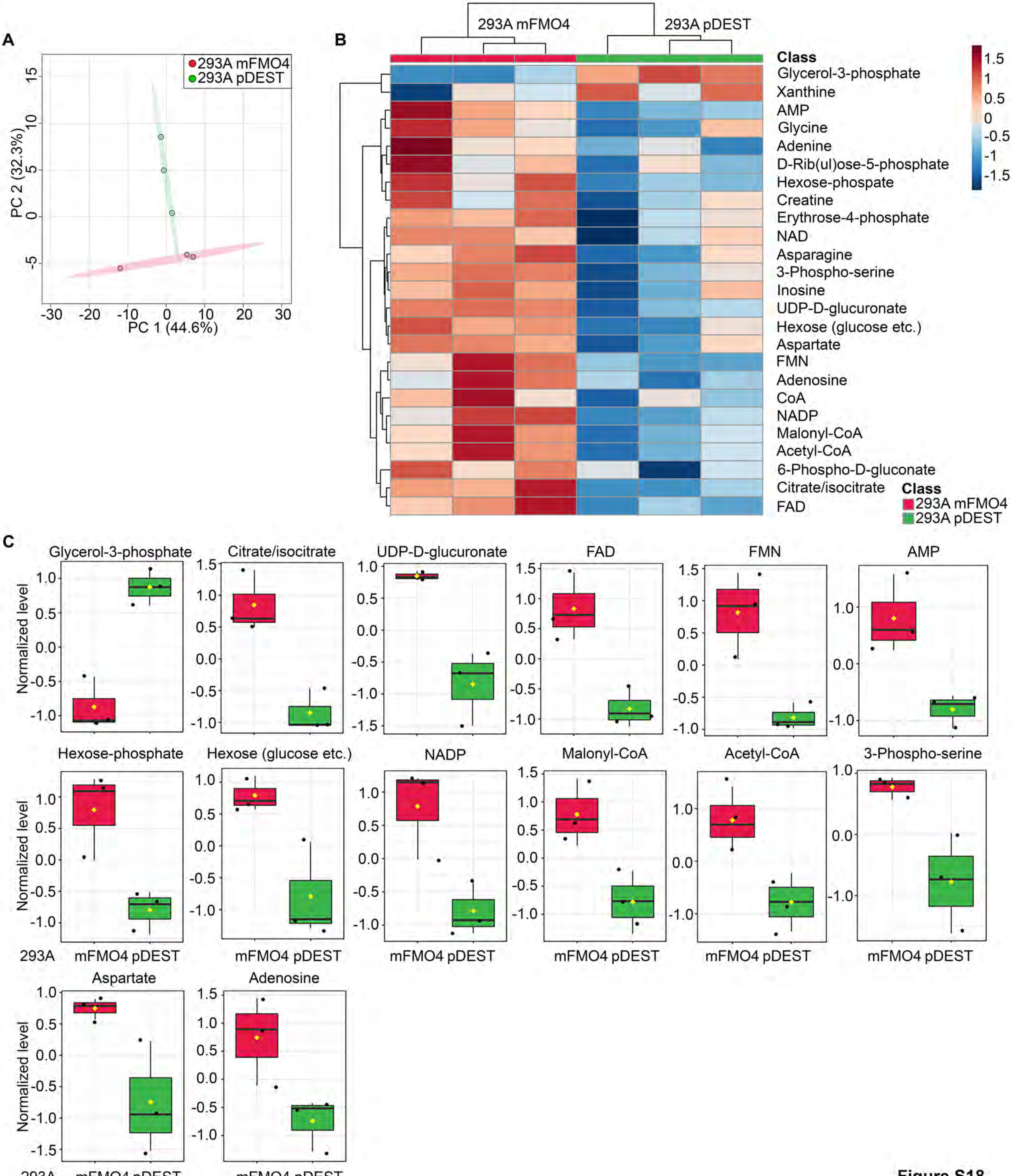
A
293A mFMO4
293A pDEST
PC 1 (44.6%)
PC 2 (32.3%)
B
293A mFMO4
293A pDEST
Class
Glycerol-3-phosphate
Xanthine
AMP
Glycine
Adenine
D-Rib(ul)ose-5-phosphate
Hexose-phospate
Creatine
Erythrose-4-phosphate
NAD
Asparagine
3-Phospho-serine
Inosine
UDP-D-glucuronate
Hexose (glucose etc.)
Aspartate
FMN
Adenosine
CoA
NADP
Malonyl-CoA
Acetyl-CoA
6-Phospho-D-gluconate
Citrate/isocitrate
FAD
C
Glycerol-3-phosphate
Citrate/isocitrate
UDP-D-glucuronate
FAD
FMN
AMP
Hexose-phosphate
Hexose (glucose etc.)
NADP
Malonyl-CoA
Acetyl-CoA
3-Phospho-serine
Aspartate
Adenosine
Normalized level
293A mFMO4 pDEST

Figure S18

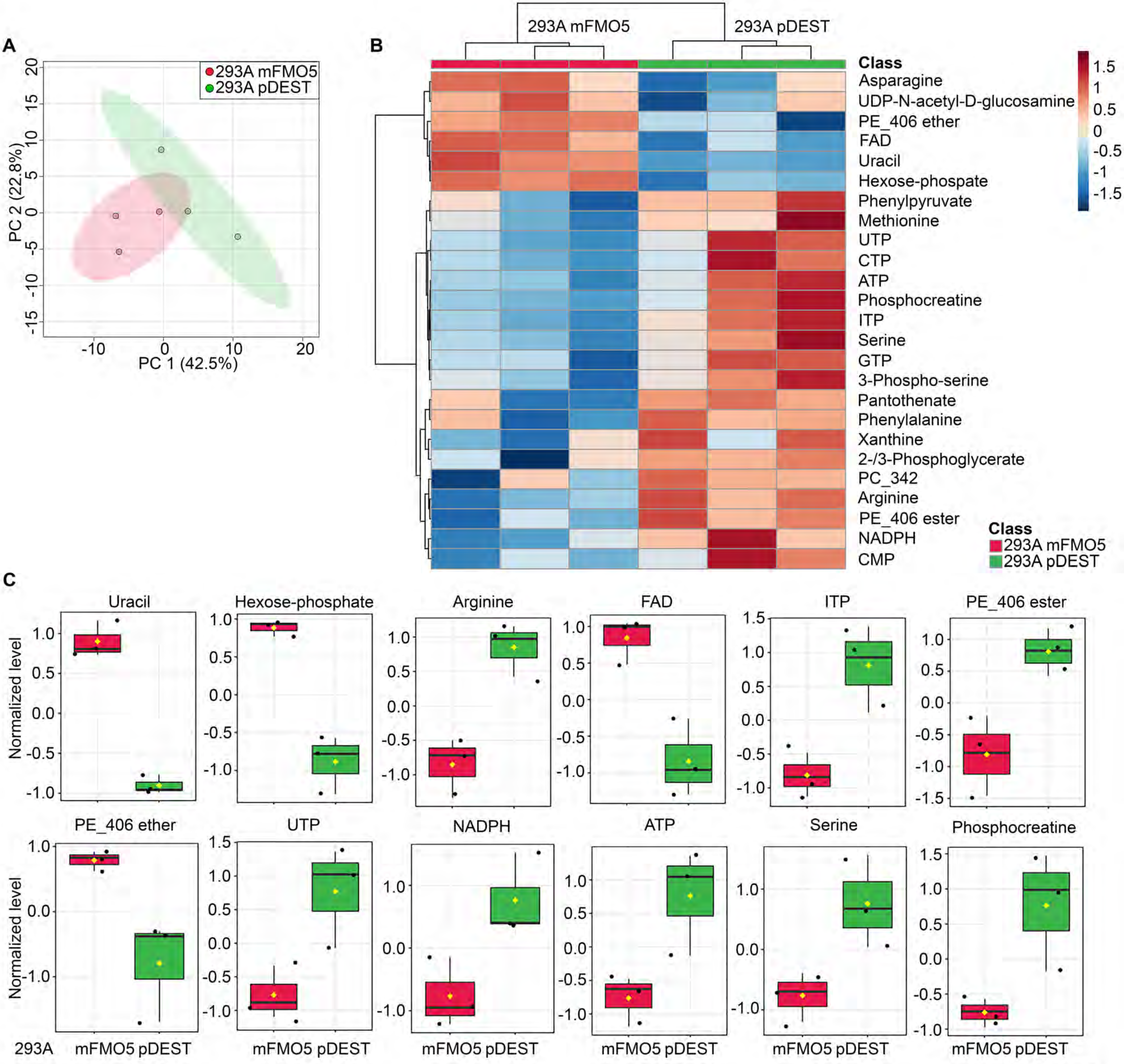
A
B
C
293A mFMO5
293A pDEST
PC 1 (42.5%)
PC 2 (22.8%)
Class
Asparagine
UDP-N-acetyl-D-glucosamine
PE_406 ether
FAD
Uracil
Hexose-phospate
Phenylpyruvate
Methionine
UTP
CTP
ATP
Phosphocreatine
ITP
Serine
GTP
3-Phospho-serine
Pantothenate
Phenylalanine
Xanthine
2-/3-Phosphoglycerate
PC_342
Arginine
PE_406 ester
NADPH
CMP
Uracil
Hexose-phosphate
Arginine
FAD
ITP
PE_406 ester
PE_406 ether
UTP
NADPH
ATP
Serine
Phosphocreatine
Normalized level
293A
mFMO5 pDEST

Figure S19

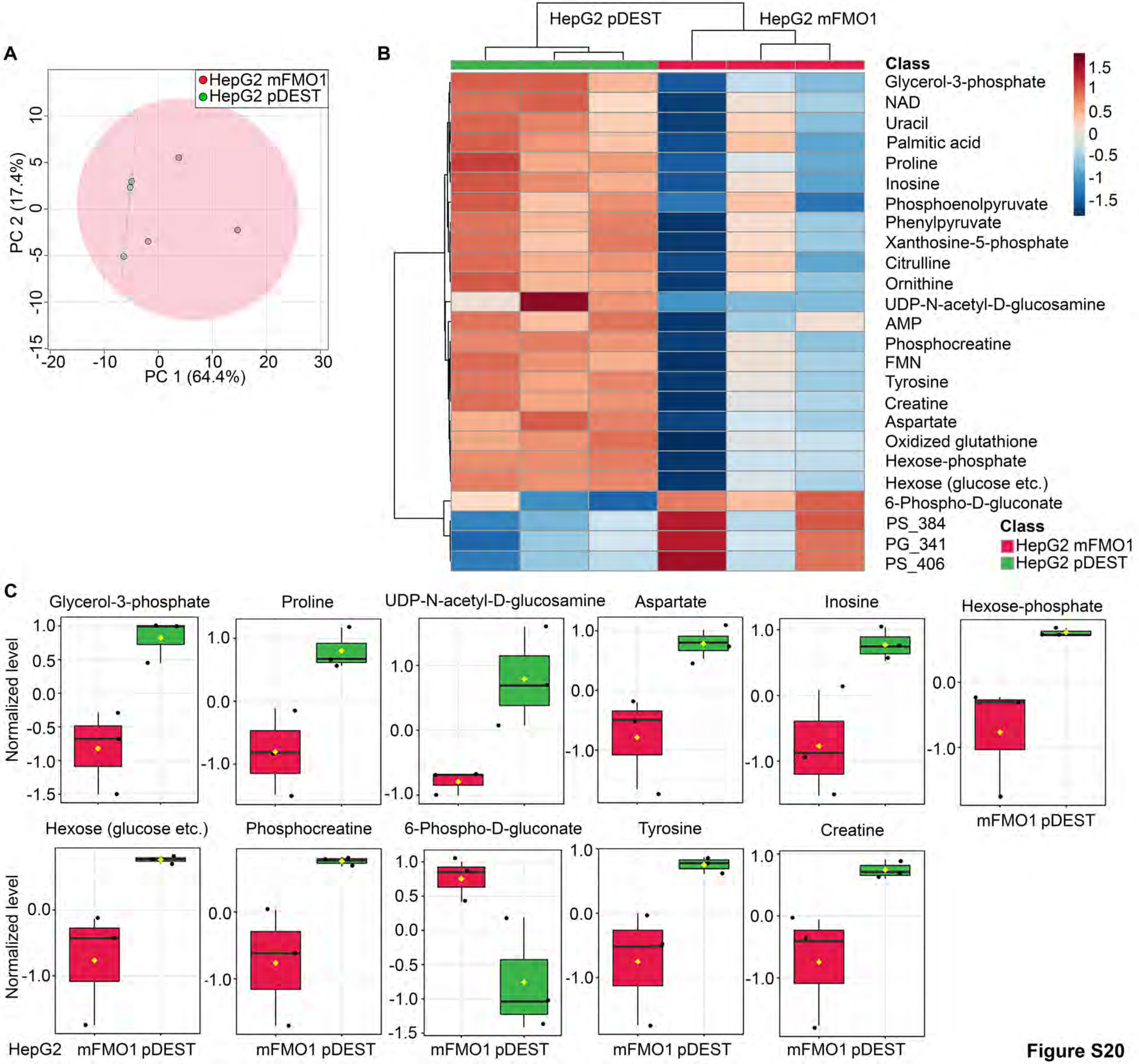

A
HepG2 mFMO1
HepG2 pDEST
PC 2 (17.4%)
PC 1 (64.4%)
B
HepG2 pDEST
HepG2 mFMO1
Class
Glycerol-3-phosphate
NAD
Uracil
Palmitic acid
Proline
Inosine
Phosphoenolpyruvate
Phenylpyruvate
Xanthosine-5-phosphate
Citrulline
Ornithine
UDP-N-acetyl-D-glucosamine
AMP
Phosphocreatine
FMN
Tyrosine
Creatine
Aspartate
Oxidized glutathione
Hexose-phosphate
Hexose (glucose etc.)
6-Phospho-D-gluconate
PS_384
PG_341
PS_406
Class
HepG2 mFMO1
HepG2 pDEST
C
Glycerol-3-phosphate
Proline
UDP-N-acetyl-D-glucosamine
Aspartate
Inosine
Hexose-phosphate
Normalized level
Hexose (glucose etc.)
Phosphocreatine
6-Phospho-D-gluconate
Tyrosine
Creatine
HepG2 mFMO1 pDEST

Figure S20

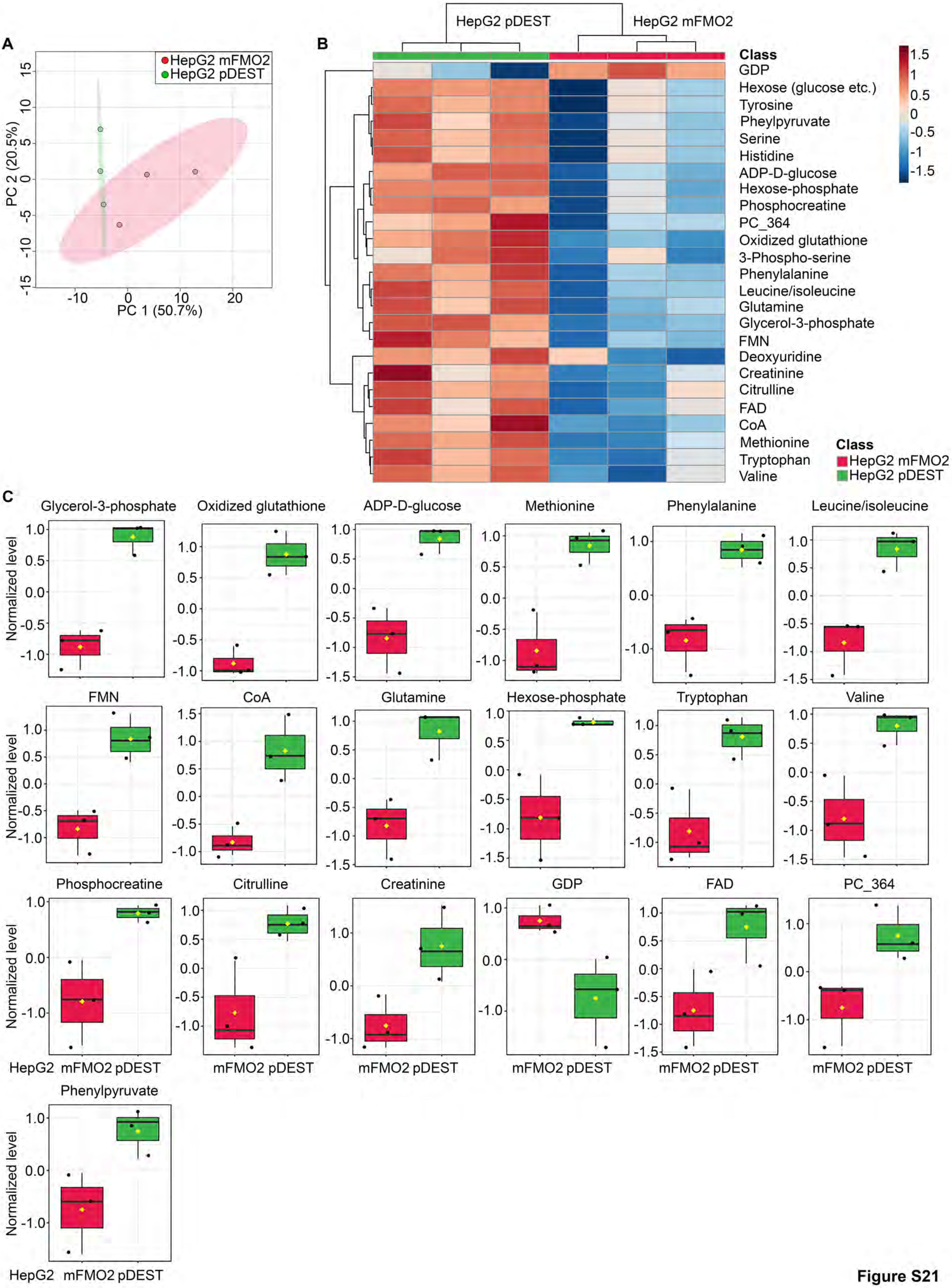

A
HepG2 mFMO2
HepG2 pDEST
PC 2 (20.5%)
PC 1 (50.7%)
B
HepG2 pDEST
HepG2 mFMO2
Class
GDP
Hexose (glucose etc.)
Tyrosine
Pheylpyruvate
Serine
Histidine
ADP-D-glucose
Hexose-phosphate
Phosphocreatine
PC_364
Oxidized glutathione
3-Phospho-serine
Phenylalanine
Leucine/isoleucine
Glutamine
Glycerol-3-phosphate
FMN
Deoxyuridine
Creatinine
Citrulline
FAD
CoA
Methionine
Tryptophan
Valine
Class
HepG2 mFMO2
HepG2 pDEST
C
Glycerol-3-phosphate
Oxidized glutathione
ADP-D-glucose
Methionine
Phenylalanine
Leucine/isoleucine
FMN
CoA
Glutamine
Hexose-phosphate
Tryptophan
Valine
Phosphocreatine
Citrulline
Creatinine
GDP
FAD
PC_364
Phenylpyruvate
Normalized level
HepG2 mFMO2 pDEST

Figure S21

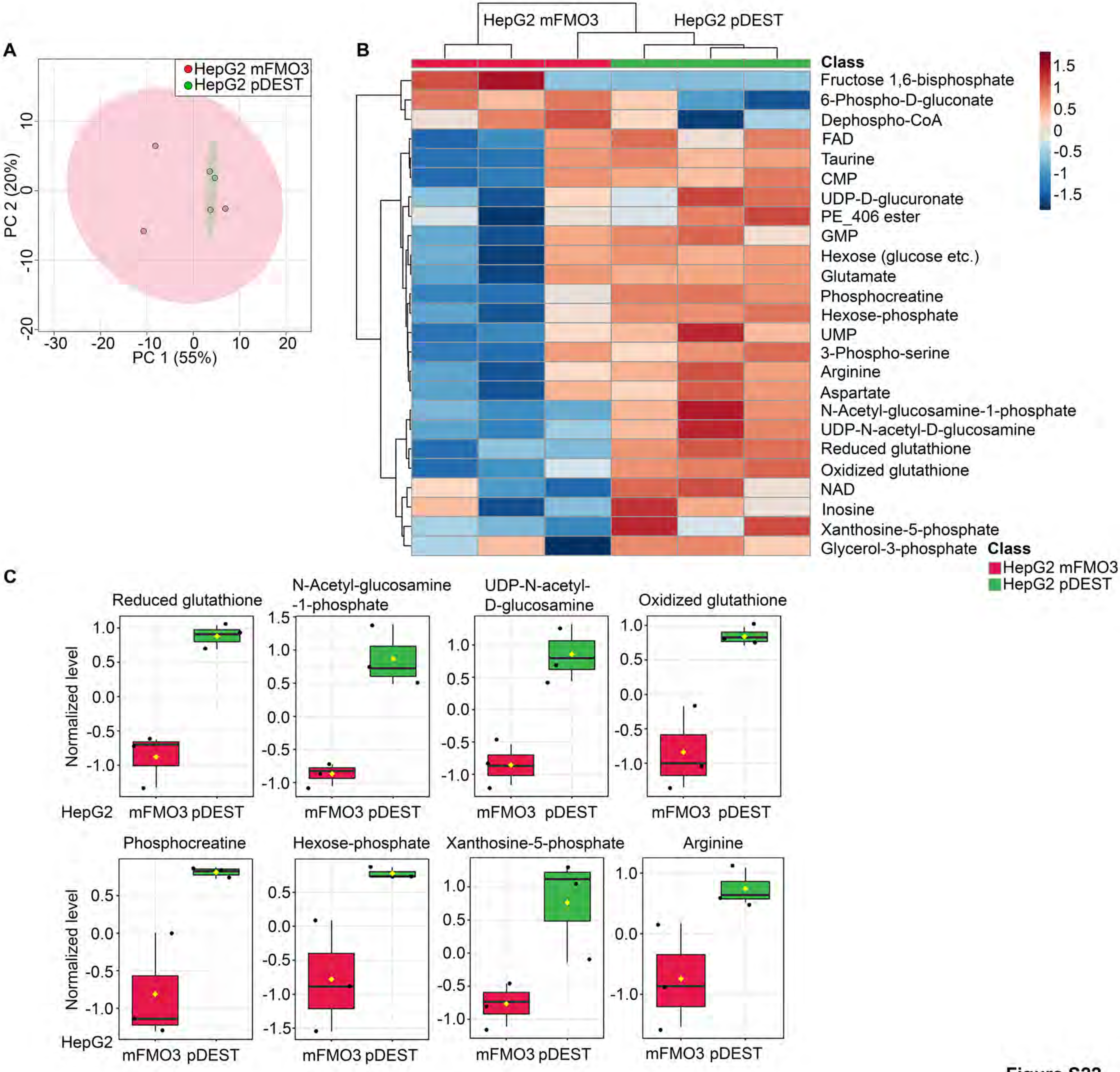
A
HepG2 mFMO3
HepG2 pDEST
PC 2 (20%)
PC 1 (55%)
-30 -20 -10 0 10 20
B
HepG2 mFMO3
HepG2 pDEST
Class
Fructose 1,6-bisphosphate
6-Phospho-D-gluconate
Dephospho-CoA
FAD
Taurine
CMP
UDP-D-glucuronate
PE_406 ester
GMP
Hexose (glucose etc.)
Glutamate
Phosphocreatine
Hexose-phosphate
UMP
3-Phospho-serine
Arginine
Aspartate
N-Acetyl-glucosamine-1-phosphate
UDP-N-acetyl-D-glucosamine
Reduced glutathione
Oxidized glutathione
NAD
Inosine
Xanthosine-5-phosphate
Glycerol-3-phosphate
1.5 1 0.5 0 -0.5 -1 -1.5
Class
HepG2 mFMO3
HepG2 pDEST
C
Reduced glutathione
N-Acetyl-glucosamine -1-phosphate
UDP-N-acetyl- D-glucosamine
Oxidized glutathione
Normalized level
HepG2 mFMO3 pDEST
Phosphocreatine
Hexose-phosphate
Xanthosine-5-phosphate
Arginine
Normalized level
HepG2 mFMO3 pDEST

Figure S22

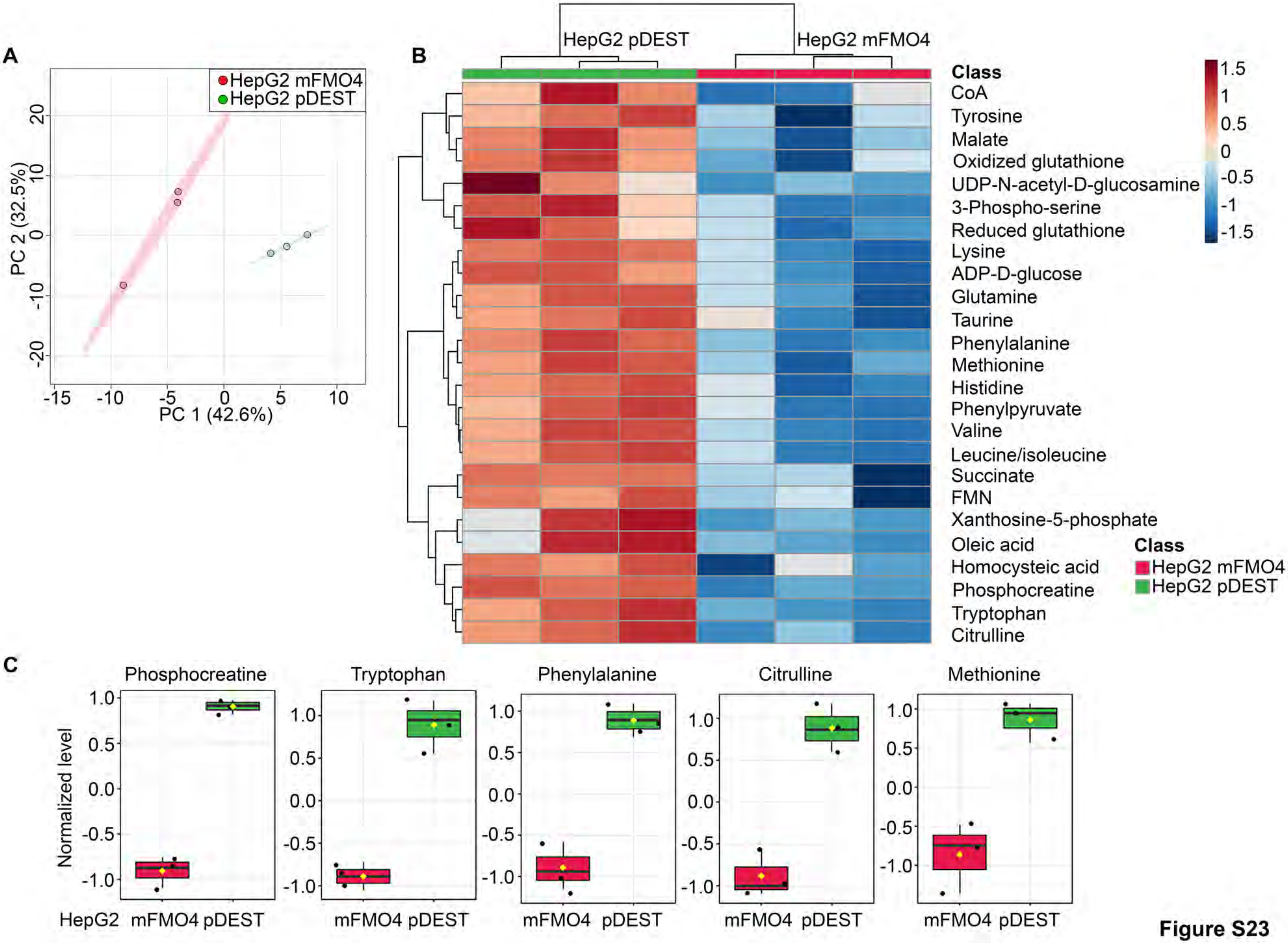

A
HepG2 mFMO4
HepG2 pDEST
PC 2 (32.5%)
PC 1 (42.6%)
B
HepG2 pDEST
HepG2 mFMO4
Class
CoA
Tyrosine
Malate
Oxidized glutathione
UDP-N-acetyl-D-glucosamine
3-Phospho-serine
Reduced glutathione
Lysine
ADP-D-glucose
Glutamine
Taurine
Phenylalanine
Methionine
Histidine
Phenylpyruvate
Valine
Leucine/isoleucine
Succinate
FMN
Xanthosine-5-phosphate
Oleic acid
Homocysteic acid
Phosphocreatine
Tryptophan
Citrulline
Class
HepG2 mFMO4
HepG2 pDEST
C
Phosphocreatine
Tryptophan
Phenylalanine
Citrulline
Methionine
Normalized level
HepG2 mFMO4 pDEST
mFMO4 pDEST
mFMO4 pDEST
mFMO4 pDEST
mFMO4 pDEST

Figure S23

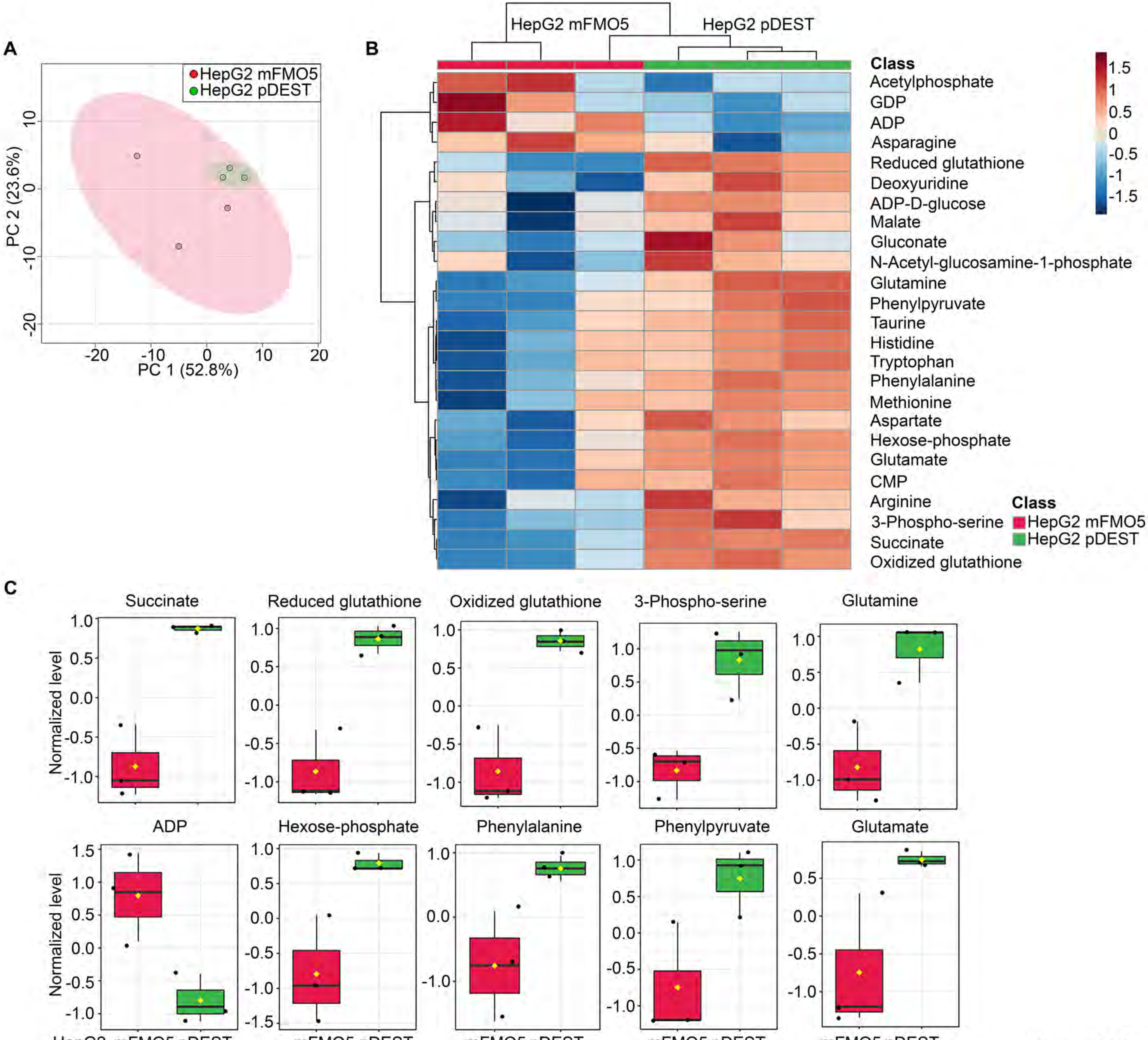
A
HepG2 mFMO5
HepG2 pDEST
PC 2 (23.6%)
PC 1 (52.8%)
B
HepG2 mFMO5
HepG2 pDEST
Class
Acetylphosphate
GDP
ADP
Asparagine
Reduced glutathione
Deoxyuridine
ADP-D-glucose
Malate
Gluconate
N-Acetyl-glucosamine-1-phosphate
Glutamine
Phenylpyruvate
Taurine
Histidine
Tryptophan
Phenylalanine
Methionine
Aspartate
Hexose-phosphate
Glutamate
CMP
Arginine
3-Phospho-serine
Succinate
Oxidized glutathione
Class
HepG2 mFMO5
HepG2 pDEST
C
Succinate
Reduced glutathione
Oxidized glutathione
3-Phospho-serine
Glutamine
ADP
Hexose-phosphate
Phenylalanine
Phenylpyruvate
Glutamate
Normalized level
HepG2 mFMO5 pDEST

Figure S24

Table S1. Significant pathways in all FMOs in HEK293A and HepG2 cells from the untargeted metabolomics analyses.

Categories of pathways	Metabolism pathways	HEK293A					HepG2				
		mFMO1	mFMO2	mFMO3	mFMO4	mFMO5	mFMO1	mFMO2	mFMO3	mFMO4	mFMO5
Amino acid metabolism	Lysine biosynthesis	0.002956	0.008387		0.044312						
Amino acid metabolism	Arginine and proline metabolism	0.005105	0.034889			0.020218			0.000471		
Amino acid metabolism	Glycine, serine and threonine metabolism	0.012043		0.027189		0.000416					0.026819
Lipid metabolism	Glycerophospholipid metabolism	0.008284								0.021601	
Amino acid metabolism	Cysteine and methionine metabolism	0.016078	0.05032			0.016976			0.045		
Metabolism of other amino acids	Taurine and hypotaurine metabolism	0.025568		0.040214		0.010637					
Protein biosynthesis	Aminoacyl-tRNA biosynthesis	0.038892	0.03475			0.002182	0.039728				
Metabolism of cofactors and vitamins	Biotin metabolism	0.016772							0.009331		
Lipid metabolism	Ether lipid metabolism	0.02853								0.00654	
Energy metabolism	Methane metabolism					0.010637			0.029844		0.011707
Metabolism of cofactors and vitamins	Pantothenate and CoA biosynthesis		0.013761	0.041069		0.001303	0.009442		0.002305		
Amino acid metabolism	Alanine, aspartate and glutamate metabolism		0.003407	0.027879	0.043404		0.039215		3.67E-05		
Metabolism of other amino acids	Cyanoamino acid metabolism		0.004053			0.001106	0.004114		0.008785		0.016647
Metabolism of cofactors and vitamins	Vitamin B6 metabolism		0.028366		0.026143		0.023693	0.021733	0.012328		
Nucleotide metabolism	Pyrimidine metabolism			0.000229							0.025852
Lipid metabolism	Synthesis and degradation of ketone bodies			0.023147							
Carbohydrate metabolism	Butanoate metabolism				0.044303				0.003186		
Metabolism of cofactors and vitamins	Porphyrin and chlorophyll metabolism				0.036707	0.010637					
Metabolism of other amino acids	Selenoamino acid metabolism					0.007628	0.015502		0.037942		
Biosynthesis of other secondary metabolites	Caffeine metabolism								0.013709		
Metabolism of other amino acids	Glutathione metabolism										0.025714
Amino acid metabolism	Lysine degradation	0.021951									
Energy metabolism	Sulfur metabolism					0.006266					
Carbohydrate metabolism	Ascorbate and aldarate metabolism				0.023666						
Energy metabolism	Nitrogen metabolism					0.009692					
Carbohydrate metabolism	Pentose phosphate pathway						0.011895				
Amino acid metabolism	Valine, leucine and isoleucine biosynthesis							0.024007			
Metabolism of other amino acids	beta-Alanine metabolism								0.013308		
Metabolism of cofactors and vitamins	Nicotinate and nicotinamide metabolism								0.017597		
Carbohydrate metabolism	Citrate cycle (TCA cycle)								0.021803		
Metabolism of other amino acids	D-Glutamine and D-glutamate metabolism								0.024314		
Amino acid metabolism	Phenylalanine, tyrosine and tryptophan biosynthesis								0.041047		
Lipid metabolism	alpha-Linolenic acid metabolism								0.031818		

Table S2. Significantly changed metabolites between FMO1-OE and control in HEK293A cells

	t.stat	p.value	negative log(p.value)	FDR
Uracil	17.931	5.69E-05	4.2452	0.005742
NADPH	-5.8281	0.004318	2.3647	0.19666
alpha-Ketoglutarate	5.3613	0.005841	2.2335	0.19666
Hexose-phosphate	4.5541	0.010385	1.9836	0.26223
FAD	4.1538	0.014217	1.8472	0.28717
Succinate	-3.8273	0.018661	1.7291	0.31413
3-Phospho-serine	-3.5006	0.024882	1.6041	0.35902
UTP	-3.2973	0.030009	1.5227	0.37887
CTP	-3.0523	0.037942	1.4209	0.38965
Pantothenate	-2.9954	0.040123	1.3966	0.38965

Table S3. Significantly changed metabolites between FMO2-OE and control in HEK293A cells

	t.stat	p.value	negative log(p.value)	FDR
2-/3-Phosphoglycerate	-8.538	0.001033	2.986	0.10431
Pantothenate	-6.939	0.002265	2.6449	0.11439
Oleic acid	6.1224	0.003605	2.4431	0.12137
Hexose-phosphate	3.2103	0.032579	1.4871	0.82262

Table S4. Significantly changed metabolites between FMO3-OE and control in HEK293A cells

	t.stat	p.value	negative log(p.value)	FDR
Gluconate	5.9189	0.004081	2.3892	0.21092
Deoxyuridine	5.4614	0.005465	2.2624	0.21092
Arginine	-4.8104	0.008583	2.0664	0.21092
Aspartate	4.5414	0.010486	1.9794	0.21092
Dephospho-CoA	4.5199	0.010659	1.9723	0.21092
Proline	4.1335	0.014453	1.84	0.21092
UTP	4.1195	0.014619	1.8351	0.21092
PC_342	-3.4896	0.025132	1.5998	0.30008
Glycerol-3-phosphate	-3.4216	0.02674	1.5728	0.30008
Dihydroxy-acetone phosphate	-3.1168	0.035637	1.4481	0.35994
CTP	2.8704	0.045451	1.3425	0.41733

Table S5. Significantly changed metabolites between FMO4-OE and control in HEK293A cells

	t.stat	p.value	negative log(p.value)	FDR
Glycerol-3-phosphate	-6.5708	0.002776	2.5566	0.25272
Citrate/isocitrate	5.0217	0.007376	2.1322	0.25272
UDP-D-glucuronate	4.997	0.007507	2.1246	0.25272
FAD	4.4448	0.011292	1.9472	0.2766
FMN	3.9914	0.016247	1.7892	0.2766
AMP	3.6888	0.02104	1.677	0.2766
Hexose-phosphate	3.5536	0.023722	1.6249	0.2766
Hexose glucose etc.	3.477	0.025422	1.5948	0.2766
NADP	3.3749	0.027917	1.5541	0.2766
Malonyl-CoA	3.2632	0.030987	1.5088	0.2766
Acetyl-CoA	3.2631	0.030989	1.5088	0.2766
3-Phospho-serine	3.2012	0.032863	1.4833	0.2766
Aspartate	2.8516	0.046322	1.3342	0.34023
Adenosine	2.7959	0.049016	1.3097	0.34023

Table S6. Significantly changed metabolites between FMO5-OE and control in HEK293A cells

	t.stat	p.value	negative log(p.value)	FDR
Uracil	12.07	0.00027	3.5683	0.027289
Hexose-phosphate	7.734	0.001505	2.8224	0.076015
Arginine	-5.148	0.006754	2.1704	0.22011
FAD	4.7891	0.008717	2.0596	0.22011
ITP	-3.9014	0.017521	1.7564	0.30684
PE_406 ester	-3.8548	0.018228	1.7393	0.30684
PE_406 ether	3.42	0.02678	1.5722	0.31754
UTP	-3.1507	0.034489	1.4623	0.31754
NADPH	-3.0861	0.036714	1.4352	0.31754
ATP	-3.0368	0.038523	1.4143	0.31754
Serine	-3.0352	0.038585	1.4136	0.31754
Phosphocreatine	-3.0291	0.038817	1.411	0.31754

Table S7. Significantly changed metabolites between FMO1-OE and control in HepG2 cells

	t.stat	p.value	negative log(p.value)	FDR
Glycerol-3-phosphate	-4.1104	0.01473	1.8319	0.26227
Proline	-3.6918	0.02099	1.6781	0.26227
UDP-N-acetyl-D-glucosamine	-3.4597	0.02583	1.588	0.26227
Aspartate	-3.3497	0.02858	1.544	0.26227
Inosine	-3.1309	0.03515	1.454	0.26227
Hexose-phosphate	-3.0844	0.03677	1.4345	0.26227
Hexose glucose etc.	-3.0726	0.0372	1.4295	0.26227
Phosphocreatine	-3.0127	0.03944	1.404	0.26227
6-Phospho-D-gluconate	2.9335	0.04266	1.37	0.26227
Tyrosine	-2.9063	0.04384	1.3581	0.26227
Creatine	-2.8011	0.04876	1.312	0.26227

Table S8. Significantly changed metabolites between FMO2-OE and control in HepG2 cells

	t.stat	p.value	negative log(p.value)	FDR
Glycerol-3-phosphate	-7.3379	0.00184	2.7361	0.10788
Oxidized glutathione	-7.0483	0.00214	2.6703	0.10788
ADP-D-glucose	-4.8669	0.00824	2.0842	0.1439
Methionine	-4.8416	0.00839	2.0762	0.1439
Phenylalanine	-4.8308	0.00846	2.0728	0.1439
Leucine/isoleucine	-4.6761	0.00947	2.0234	0.1439
FMN	-4.6076	0.00997	2.0012	0.1439
CoA	-4.3114	0.01253	1.902	0.15822
Glutamine	-4.1566	0.01418	1.8482	0.15918
Hexose-phosphate	-3.848	0.01833	1.7367	0.1735
Tryptophan	-3.8127	0.0189	1.7236	0.1735
Valine	-3.6255	0.02225	1.6527	0.18726
Phosphocreatine	-3.4805	0.02534	1.5962	0.19688
Citrulline	-3.128	0.03526	1.4528	0.24776
Creatinine	-2.907	0.04381	1.3584	0.24776
GDP	2.8912	0.04451	1.3516	0.24776
FAD	-2.8905	0.04454	1.3512	0.24776
PC_364	-2.8703	0.04546	1.3424	0.24776
Phenylpyruvate	-2.8455	0.04661	1.3315	0.24776

Table S9. Significantly changed metabolites between FMO3-OE and control in HepG2 cells

	t.stat	p.value	negative log(p.value)	FDR
Reduced glutathione	-7.1992	0.001973	2.7049	0.18814
N-Acetyl-glucosamine-1-phosphate	-6.0022	0.003877	2.4115	0.18814
UDP-N-acetyl-D-glucosamine	-5.4277	0.005588	2.2527	0.18814
Oxidized glutathione	-4.6651	0.009553	2.0199	0.24121
Phosphocreatine	-3.898	0.017572	1.7552	0.35495
Hexose-phosphate	-3.2787	0.030538	1.5152	0.51406
Xanthosine-5-phosphate	-3.0724	0.037206	1.4294	0.53683
Arginine	-2.8181	0.047919	1.3195	0.60497

Table S10. Significantly changed metabolites between FMO4-OE and control in HepG2 cells

	t.stat	p.value	negative log(p.value)	FDR
Phosphocreatine	-16.081	8.75E-05	4.0582	0.008832
Tryptophan	-8.8043	0.000918	3.0371	0.032105
Phenylalanine	-8.7177	0.000954	3.0206	0.032105
Citrulline	-7.4791	0.001709	2.7673	0.043148
Methionine	-5.7203	0.004622	2.3352	0.077593

Table S11. Significantly changed metabolites between FMO5-OE and control in HepG2 cells

	t.stat	p.value	negative log(p.value)	FDR
Succinate	-6.4728	0.002935	2.5323	0.18685
Reduced glutathione	-5.8531	0.004251	2.3715	0.18685
Oxidized glutathione	-5.4381	0.00555	2.2557	0.18685
3-Phospho-serine	-4.4365	0.011364	1.9445	0.28695
Glutamine	-4.07	0.015223	1.8175	0.3075
ADP	3.5722	0.02333	1.6321	0.35339
Hexose-phosphate	-3.5181	0.024492	1.611	0.35339
Phenylalanine	-2.9744	0.040966	1.3876	0.40912
Phenylpyruvate	-2.8497	0.04641	1.3334	0.40912
Glutamate	-2.8264	0.047519	1.3231	0.40912
